# A Phase 2 Randomized Trial of a Rifapentine plus Moxifloxacin-Based Regimen for Treatment of Pulmonary Tuberculosis

**DOI:** 10.1371/journal.pone.0154778

**Published:** 2016-05-09

**Authors:** Marcus B. Conde, Fernanda C. Q. Mello, Rafael Silva Duarte, Solange C. Cavalcante, Valeria Rolla, Margareth Dalcolmo, Carla Loredo, Betina Durovni, Derek T. Armstrong, Anne Efron, Grace L. Barnes, Mark A. Marzinke, Radojka M. Savic, Kelly E. Dooley, Silvia Cohn, Lawrence H. Moulton, Richard E. Chaisson, Susan E. Dorman

**Affiliations:** 1 Instituto de Doenças do Tórax, Universidade Federal do Rio de Janeiro, Rio de Janeiro, Brazil; 2 Instituto de Microbiologia Paulo de Góes, Universidade Federal do Rio de Janeiro, Rio de Janeiro, Brazil; 3 Municipal Health Secretariat, Rio de Janeiro, Brazil; 4 Instituto Nacional de Infectologia Evandro Chagas, Fiocruz, Rio de Janeiro, Brazil; 5 Centro de Referência Hélio Fraga, Ministério da Saúde, Fiocruz Rio de Janeiro, Brazil; 6 Department of Medicine, Johns Hopkins University School of Medicine, Baltimore, Maryland, United States of America; 7 University of California San Francisco, San Francisco, California, United States of America; 8 Department of International Health, Johns Hopkins Bloomberg School of Public Health, Baltimore, Maryland, United States of America; University of Cape Town, SOUTH AFRICA

## Abstract

**Background:**

The combination of rifapentine and moxifloxacin administered daily with other anti-tuberculosis drugs is highly active in mouse models of tuberculosis chemotherapy. The objective of this phase 2 clinical trial was to determine the bactericidal activity, safety, and tolerability of a regimen comprised of rifapentine, moxifloxacin, isoniazid, and pyrazinamide administered daily during the first 8 weeks of pulmonary tuberculosis treatment.

**Methods:**

Adults with sputum smear-positive pulmonary tuberculosis were randomized to receive either rifapentine (approximately 7.5 mg/kg) plus moxifloxacin (investigational arm), or rifampin (approximately 10 mg/kg) plus ethambutol (control) daily for 8 weeks, along with isoniazid and pyrazinamide. The primary endpoint was sputum culture status at completion of 8 weeks of treatment.

**Results:**

121 participants (56% of accrual target) were enrolled. At completion of 8 weeks of treatment, negative cultures using Löwenstein-Jensen (LJ) medium occurred in 47/60 (78%) participants in the investigational arm vs. 43/51 (84%, p = 0.47) in the control arm; negative cultures using liquid medium occurred in 37/47 (79%) in the investigational arm vs. 27/41 (66%, p = 0.23) in the control arm. Time to stable culture conversion was shorter for the investigational arm vs. the control arm using liquid culture medium (p = 0.03), but there was no difference using LJ medium. Median rifapentine area under the concentration-time curve (AUC_0-24_) was 313 mcg*h/mL, similar to recent studies of rifapentine dosed at 450–600 mg daily. Median moxifloxacin AUC_0-24_ was 28.0 mcg*h/mL, much lower than in trials where rifapentine was given only intermittently with moxifloxacin. The proportion of participants discontinuing assigned treatment for reasons other than microbiological ineligibility was higher in the investigational arm vs. the control arm (11/62 [18%] vs. 3/59 [5%], p = 0.04) although the proportions of grade 3 or higher adverse events were similar (5/62 [8%] in the investigational arm vs. 6/59 [10%, p = 0.76] in the control arm).

**Conclusion:**

For intensive phase daily tuberculosis treatment in combination with isoniazid and pyrazinamide, a regimen containing moxifloxacin plus low dose rifapentine was at least as bactericidal as the control regimen containing ethambutol plus standard dose rifampin.

**Trial Registration:**

www.ClinicalTrials.gov NCT00728507

## Introduction

Shortening treatment of drug-susceptible pulmonary tuberculosis to less than six months is a public health priority. Shorter treatment durations might increase treatment completion and cure rates, reduce the burden of overstretched tuberculosis clinics, and possibly decrease transmission [1]. Strategies for increasing regimen activity in order to shorten treatment duration include the development of new drugs and the optimization of the use of existing drugs.

Rifamycins, such as rifampin, are key sterilizing agents during tuberculosis treatment, but recommended doses of rifampin are at the low end of the dose-response curve [2–9]. Therefore, optimizing rifamycin exposure is a strategy for optimizing regimen potency and shortening treatment duration. Replacement of ethambutol with a more active drug is another strategy to increase regimen potency and decrease treatment duration, since ethambutol has relatively weak anti-tuberculosis activity and its main role is to prevent acquired drug resistance.

We conducted a phase 2 trial to assess the bactericidal activity of an intensive phase regimen that combined both of these strategies for increasing regimen potency (S1 File). The investigational regimen replaced rifampin with rifapentine and also replaced ethambutol with moxifloxacin. Rifapentine is a ring-substituted rifamycin that has potent anti-tuberculosis activity in vitro and when administered daily in animal models [10–17]. Moxifloxacin, a fluoroquinolone, has potent anti-tuberculosis activity in vitro and in animal models, is an important component of the treatment of multidrug-resistant tuberculosis, and augments regimen bactericidal activity when used in place of ethambutol during the initial phase of treatment for drug-susceptible tuberculosis [11–24].

## Methods

### Study Population

Participants were enrolled at three sites in Rio de Janeiro, Brazil. Inclusion criteria were age ≥18 years, suspected pulmonary tuberculosis with a sputum smear that was positive for acid fast bacilli, liver and renal chemistries within specified ranges around normal, and HIV testing. Exclusionary criteria were pregnancy, more than 7 days of anti-tuberculosis treatment within the preceding 6 months, more than 7 days of fluoroquinolones within the preceding 3 months, and for HIV-infected participants a CD4 lymphocyte count <350 cells/mm^3^ and/or planned antiretroviral therapy during the first 8 weeks of tuberculosis treatment. Enrolled participants were excluded and study treatment was stopped if baseline sputum cultures either were negative for *M*. *tuberculosis* or grew *M*. *tuberculosis* resistant to isoniazid and/or rifampin (late exclusions). Participants provided individual written informed consent. This study was approved by the National Committee for Ethics in Research in Brazil, and by institutional review boards at each Brazilian site and Johns Hopkins Medicine. This trial is registered as NCT00728507 at www.ClinicalTrials.gov. (URL https://clinicaltrials.gov/ct2/show/NCT00728507?term=Dorman&rank=5)

### Design, Intervention, and Evaluations

Participants were randomly assigned in blocks to the investigational arm or the control arm; randomization was stratified by enrollment site and by the presence of cavitation on baseline chest radiograph. The investigational regimen was comprised of rifapentine, moxifloxacin, isoniazid, and pyrazinamide. The control arm (standard treatment) was comprised of rifampin, ethambutol, isoniazid, and pyrazinamide. Individuals in both arms also received pyridoxine (vitamin B6). Medications were administered once daily, 7 days per week, for 8 weeks. The rifapentine dose was approximately 7.5 mg/kg (300 mg for individuals weighing 45 kg or less, and 450 mg for all others), and the rifampin dose was approximately 10 mg/kg (450 mg for individuals weighing 45 kg or less, and 600 mg for all others). The moxifloxacin dose was 400 mg. Doses of isoniazid, pyrazinamide, ethambutol, and pyridoxine were in accordance with published guidelines [25]. Study medicines were administered by directly observed therapy on five days per week, and on weekends participants took self-administered pre-packaged medicines. The investigational regimen was typically administered with food (bread plus cheese) in order to maximize rifapentine bioavailability, whereas the control regimen was typically administered without food because food delays rifampin absorption [26,27]. The study was open-label; microbiologists did not have access to information about treatment assignment. After completing the 8-week study treatment, participants continued tuberculosis treatment with a conventional continuation phase regimen, typically isoniazid plus rifampin 600 mg daily for four additional months.

Sputum was collected at baseline, weekly for 8 weeks (two sputa were collected at week 8), and during continuation phase treatment at weeks 12, 16, 20, and 26. Sputa were processed using conventional N-acetyl-L-cysteine-NaOH methods and cultured using Löwenstein-Jensen (LJ) solid medium and BACTEC Mycobacterial Growth Indicator Tube (MGIT, Becton Dickinson and Co., Franklin Lakes, NJ) liquid medium with the MGIT automated system. The first approximately 10% of participants enrolled did not have MGIT cultures performed because capacity was not available at the study laboratory. For each participant the baseline *M*. *tuberculosis* isolate was tested for susceptibility to isoniazid and rifampin using the MGIT system; isolates were not tested for susceptibility to moxifloxacin. Information on symptoms, and blood for alanine aminotransferase, bilirubin, creatinine, and complete blood count were collected at baseline and at weeks 2, 4, 6, 8, and 12.

Blood was drawn for population pharmacokinetic (PK) analysis in the investigational arm only. Sampling was performed approximately three weeks after initiation of treatment. PK samples for moxifloxacin and rifapentine were collected pre-dose, 45 minutes, 1.5 hours, 4 hours, and 24 hours after dosing. The liquid chromatography-tandem mass spectrometry (LC-MS/MS) method used to quantify rifapentine was described previously [28]. The analytical range was 50 to 80,000 ng/mL. Moxifloxacin was isolated via protein precipitation and the analyte was separated using an Acquity BEH C18 1.7 um (2.1 x 50 mm) column (Waters Corporation), on an Acquity UPLC system (Waters) interfaced with an API 5500 QTrap (SCIEX). Moxifloxacin concentrations were determined from a standard curve that measured the peak area ratios of known concentrations of drug to an isotopically labeled internal standard. The analytical measuring range of the moxifloxacin assay was 10 to 5000 ng/mL.

### Data Analysis and Statistical Considerations

The primary efficacy endpoint was sputum culture status assessed using LJ media at completion of 8 weeks of treatment. Results from LJ and MGIT media were analyzed separately. Time to stable culture conversion was a secondary efficacy endpoint. Stable culture conversion was defined as having two consecutive sputum specimens culture negative for *M*. *tuberculosis*, with no subsequent culture that was positive. The primary tolerability endpoint was discontinuation of assigned treatment during the first eight weeks. Safety parameters included the frequency and severity of adverse events.

For efficacy analyses, the primary analysis population was the modified intention-to-treat (MITT) population that included participants with growth of *M*. *tuberculosis* that was susceptible to isoniazid and rifampin in a baseline culture; participants with week 8 cultures that were missing or contaminated were considered to be positive for *M*. *tuberculosis* (i.e. missing = failure). An additional exploratory analysis was performed in which, for participants with week 8 cultures that were missing or contaminated, the most recent prior determinate result was carried forward. A per-protocol population was comprised of MITT population participants who, in addition, completed assigned study intensive phase treatment within 56–70 calendar days and had an end-of-intensive phase culture that was evaluable (i.e. not missing or contaminated). The intention-to-treat (ITT) analysis population was comprised of all randomized participants and was used for tolerability and safety analyses. Proportions were compared using the Fisher exact test. Time to culture conversion was plotted by the Kaplan-Meier method and survival curves were compared using the log-rank test. Comparisons of median times to stable culture conversion were performed using the Wilcoxon two sample rank sum test for survival data.

This was designed as a superiority study. The null hypothesis was that there would be no difference between investigational and control arms in the proportion of subjects whose sputum obtained at completion of 8 weeks of treatment was negative for *M*. *tuberculosis* on LJ culture. Based on prior data from the study laboratory we estimated that, at completion of 8 weeks of treatment, 63% of subjects in the control arm would have culture negative sputum. We reasoned that an absolute increase of 20% over the standard regimen might allow overall treatment shortening on the basis of prior trials showing that the addition of pyrazinamide to regimens including isoniazid and rifampin increased week 8 culture conversion by an average of about 13% and allowed reduction of total treatment duration by 3 months [29]. Therefore we postulated that the dual substitutions in the investigational arm would improve regimen activity such that 83% of subjects were culture negative. To detect an increase in culture conversion from 63% to 83% with a two-sided test at the 0.05 level with 80% power required 86 subjects/arm. We increased the calculated sample size by approximately 20% to account for microbiological late exclusions, and therefore the enrollment target was 108/arm (216 total).

Population PK models for rifapentine and moxifloxacin were developed using nonlinear mixed effects modeling, NONMEM software, as previously described [28]. From these models, individual post-hoc Bayesian estimates of maximum concentration (C_max_) and area under the concentration-time curve over 24 hours (AUC_0-24_) were computed.

## Results

### Study Population

**Fig 1:**
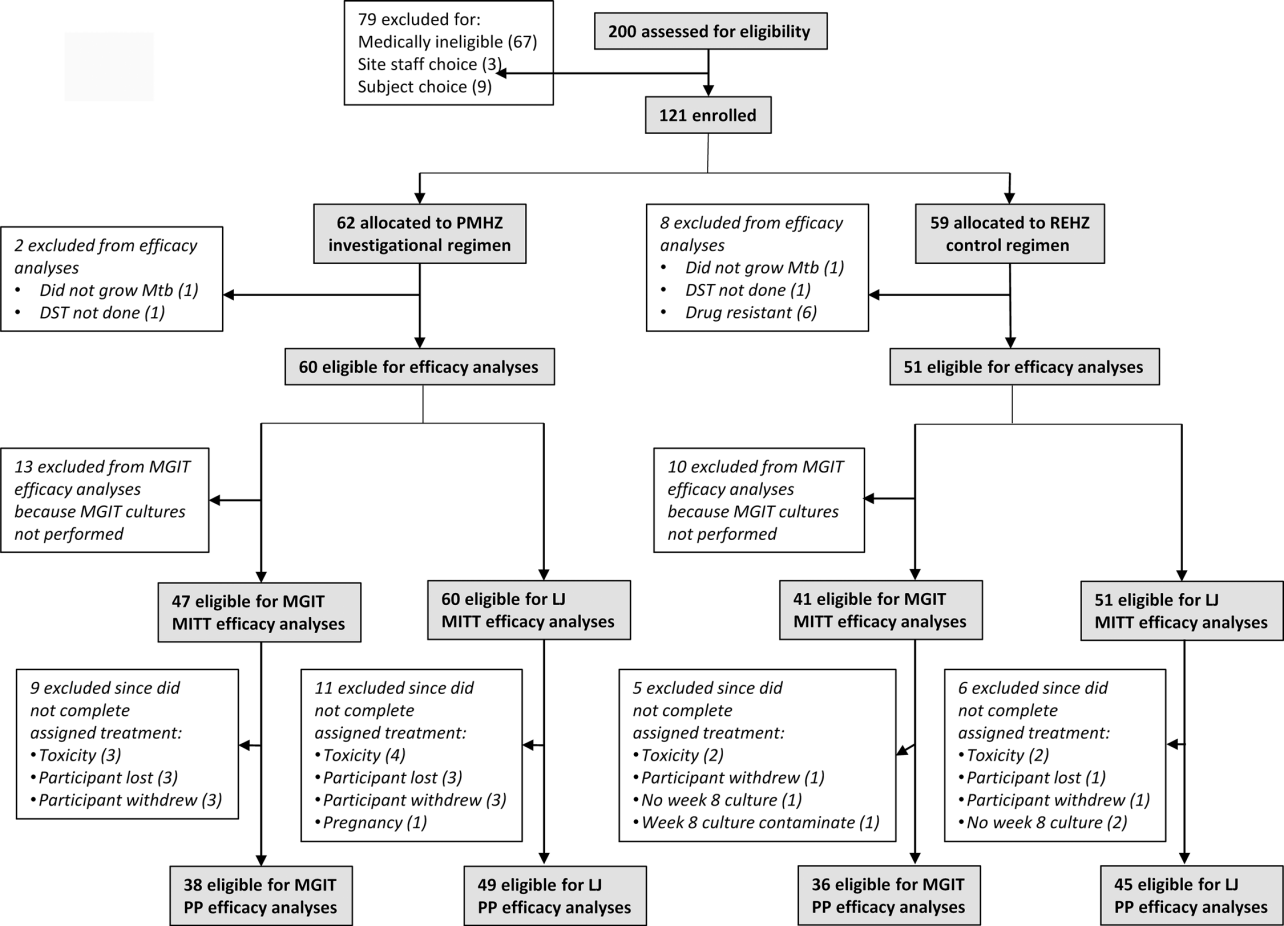
Enrollment and disposition of study participants. Abbreviations: PMHZ, investigational regimen comprised of rifapentine/moxifloxacin/isoniazid/pyrazinamide; REHZ, control regimen comprised of rifampin/ethambutol/isoniazid/pyrazinamide; LJ, Löwenstein-Jensen culture; MGIT, Mycobacterial Growth Indicator Tube culture; MITT, modified intention-to-treat analysis population; PP, per-protocol analysis population; DST, drug susceptibility testing; Mtb, *Mycobacterium tuberculosis*.

Between November 2009 and August 2013, 121 participants (56% of the accrual target of 216) were enrolled (S2 File). Enrollment was slower than expected due to regulatory delays, temporary closure of a recruitment site due to military activity, closure of the main clinic site due to building demolition, and expiration of funding. Accrual was stopped before meeting enrollment targets. The ITT analysis population was comprised of 62 participants allocated to the investigational arm and 59 participants allocated to the control arm (Fig 1). Two participants in the investigational arm and 8 participants in the control arm were classified as early exclusions for microbiological ineligibility. MGIT cultures were not performed for 23 participants (13 in the investigational arm and 10 in the control arm) enrolled early in the trial, and therefore these individuals were excluded from MGIT efficacy analyses.

Table 1 shows participant characteristics at enrollment. Among 121 participants enrolled, median age was 32 years, 83 (69%) were male, and 92 (76%) had cavitary lesions on chest X-ray. All participants were HIV-negative. Approximately one-quarter of participants received some TB treatment prior to their first dose of study treatment, but there was no difference between study arms. Based on body weight at enrollment, during study treatment in the investigational arm 60/62 (97%) of participants received rifapentine 450 mg and 2/62 (3%) received rifapentine 300 mg; in the control arm 55/59 (93%) received rifampin 600 mg and 4/59 (7%) received rifampin 450 mg.

**Table 1:** Baseline characteristics of participants in the intention-to-treat analysis population. Abbreviations: AST, aspartate aminotransferase; IQR, inter-quartile range.

Characteristic	Overall n = 121	Investigational Arm PMHZ (n = 62)n = 62	Control ArmArm REHZ (n = 59)
Median age, years (IQR)	32 (24,47)	33 (25, 48)	30 (24, 45)
Male, n (%)	83 (69)	47 (76)	36 (61)
Current or former cigarette smoking, n (%)	60 (50)	30 (48)	30 (51)
HIV-positive, n (%)	0	0	0
Median body mass index, kg/m^2^ (IQR)	20 (19,23)	20 (19,22)	20 (19,23)
Baseline smear grade 2 or higher, n (%)	78 (64)	40 (65)	38 (64)
Cavitation on chest X-ray at enrollment, n (%)	92 (76)	47 (76)	45 (76)
AST > upper limit of normal, n (%)	18 (15)	10 (16)	8 (14)
Bilirubin > upper limit of normal, n (%)	1 (0.8)	0	1 (1.7)
Median Karnofsky score (IQR)	90 (80, 90)	90 (80, 90)	90 (80, 90)
Tuberculosis treatment initiated prior to study drugs, n (%)	31 (26)	17 (27)	14 (24)
Median number of days of pre-study tuberculosis treatment (IQR)	0	0	0

### Efficacy Endpoints

For the MITT analysis population in which missing results were classified as positive cultures, the proportions of participants with negative cultures at completion of intensive phase study treatment were 47/60 (78.3%) in the investigational arm vs. 43/51 (84.3%, p = 0.47) in the control arm using LJ culture, and 37/47 (78.7%) in the investigational arm vs. 27/41 (65.9%, p = 0.23) in the control arm using MGIT culture (Table 2). When the last determinate result was carried forward, proportions with negative cultures at completion of intensive phase treatment were 53/60 (88.3%) in the investigational arm vs. 46/51 (90.2%, p = 1.00) in the control arm using LJ culture, and 40/47 (85.1%) in the investigational arm vs. 29/41 (70.7%, p = 0.12) in the control arm using MGIT culture.

**Table 2:** Proportions of participants with negative cultures at completion of intensive phase treatment. Abbreviation: CI, confidence interval; MGIT, Mycobacterial Growth Indicator Tube.

Culture Medium for Endpoint Assessment	Investigational Arm PMHZ	Control Arm REHZ	Difference in % (95% CI)	P value
*Modified intention-to-treat analysis population*: *missing week 8 result classified as culture positive*				
Löwenstein-Jensen solid culture	47/60 (78.3%)	43/51 (84.3%)	-6.0 (-19.9, 10.3)	0.47
MGIT liquid culture	37/47 (78.7%)	27/41 (65.9%)	12.9 (-7.8, 31.7)	0.23
*Modified intention-to-treat analysis population*: *if week 8 result missing*, *then last result carried forward*				
Löwenstein-Jensen solid culture	53/60 (88.3%)	46/51 (90.2%)	-1.9 (-12.6, 10.7)	1.00
MGIT liquid culture	40/47 (85.1%)	29/41 (70.7%)	14.4 (-5.0, 30.5)	0.12
*Per-protocol analysis population*				
Löwenstein-Jensen solid culture	46/49 (93.9%)	41/45 (91.1%)	2.8(-8.1, 12.0)	0.71
MGIT liquid culture	36/38 (94.7%)	26/36 (72.2%)	22.5(3.0, 31.4)	0.01

**Fig 2:**
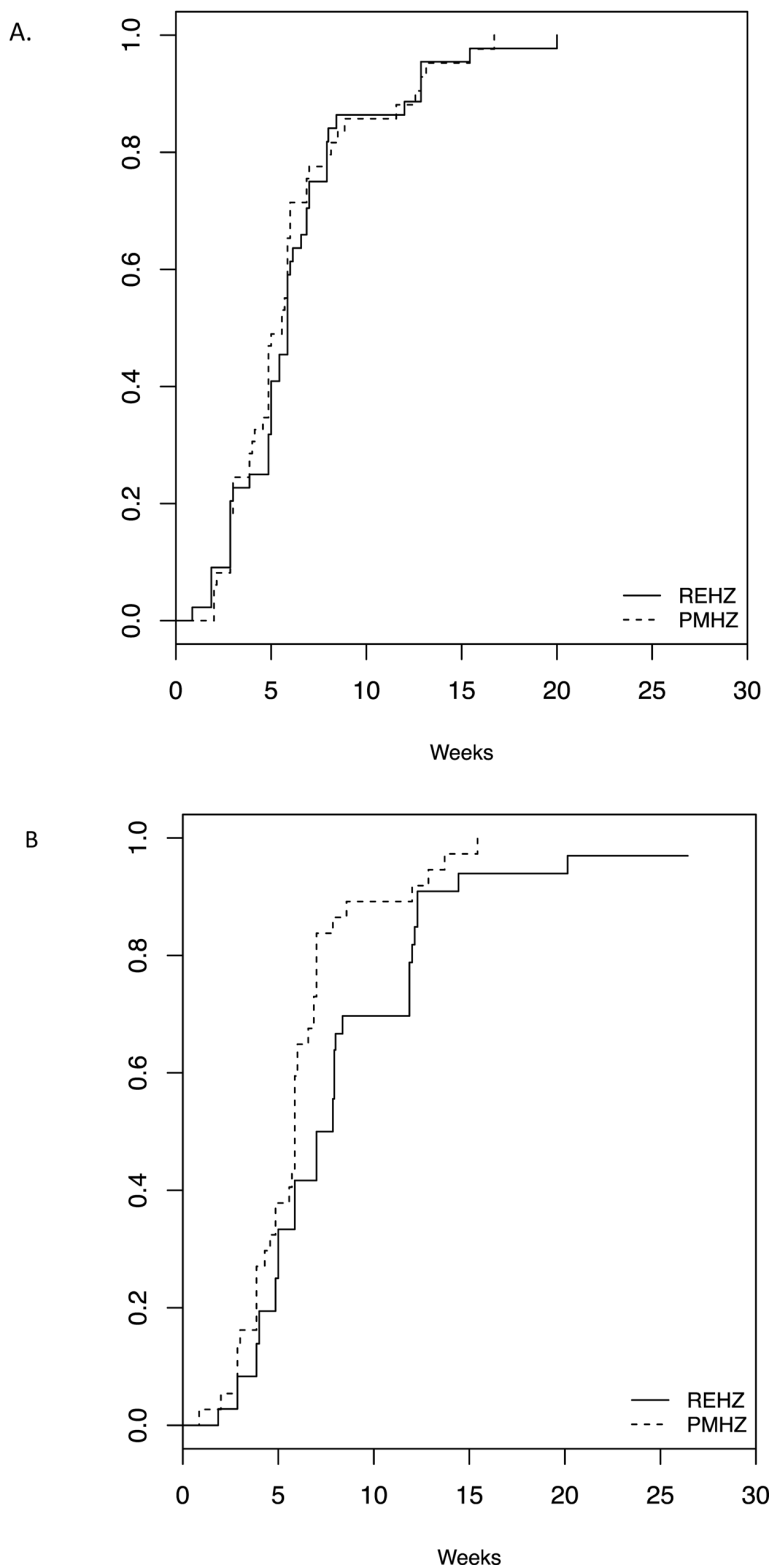
Kaplan-Meier time to stable culture conversion, per-protocol analysis population. Solid lines, control REHZ regimen comprised of rifampin/ethambutol/isoniazid/pyrazinamide. Dashed lines, investigational PMHZ regimen comprised of rifapentine/moxifloxacin/isoniazid/pyrazinamide. (A) Löwenstein-Jensen (LJ) solid culture medium (p = 0.67 for comparing equality of the survival curves), and (B) Mycobacterial Growth Indicator Tube (MGIT) liquid culture medium (p = 0.03).

For the per-protocol analysis population, the proportions of participants with negative cultures at completion of intensive phase treatment were 46/49 (93.9%) in the investigational arm vs. 41/45 (91.1%, p = 0.71) in the control arm using LJ culture, and 36/38 (94.7%) in the investigational arm vs. 26/36 (72.2%, p = 0.01) in the control arm using MGIT culture (Table 2). The time to stable culture conversion was significantly shorter (median 5.9 [95% CI 4.9, 6.9] weeks vs 7.4 [5.9, 8.4] weeks, p = 0.03) for the investigational arm when MGIT liquid medium was used, but there was no difference using LJ medium (median 5.6 [4.9, 6.0] weeks for the investigational arm vs. 5.9 [5.0, 6.9] weeks for the control arm, p = 0.67, Fig 2).

### Tolerability and Safety

Among enrolled participants 12/62 (19%) in the investigational arm vs. 9/59 (15%, p = 0.63) in the control arm permanently discontinued assigned intensive phase treatment (Table 3). Higher proportions in the investigational arm (11/62 [18%]) than in the control arm (3/59 [5%], p = 0.04) discontinued treatment due to toxicity, default, or participant withdrawal from the study. There were no deaths within 70 days of treatment assignment. There were no differences in proportions of subjects with grade 3 or higher adverse events in the investigational arm (5/62 [8%]) vs. 6/59 the control arm (6/59 [10%], p = 0.76). Grade 3 or higher hepatotoxicity occurred in 0/62 participants in the investigational arm vs. 3/59 (5%, p = 0.11) in the control arm. Two participants among 62 in the investigational arm vs. 0/59 in the control arm had grade 3 flu-like syndrome, and one participant among 62 in the investigational arm vs 0/59 in the control group had grade 3 rash (Table 3)

**Table 3:** Discontinuation of assigned treatment during the intensive phase, and adverse events within the first 70 days after the initial dose of study drugs. Notes: ^1^: The study regimen was discontinued in both participants. In one participant the re-introduction of isoniazid replicated the flu-like symptoms experienced while on the study regimen. In the other participant the re-introduction of rifapentine or pyrazinamide replicated the flu-like symptoms experienced while on the study regimen. ^2^: The study regimen was discontinued. The rash recurred with re-introduction of isoniazid.

	Investigational Arm PMHZ (n = 62)	Control Arm REHZ (n = 59)	P value
**Regimen permanently discontinued, n (%)**	**12 (19%)**	**9 (15%)**	**0.63**
Regimen permanently discontinued based solely on microbiological late exclusion, n (%)	1 (2%)	6 (10%)	0.06
Regimen permanently discontinued for reasons other than microbiological late exclusion, n (%)	11 (%)	3 (%)	0.04
**Discontinuation reasons other than microbiological late exclusion**			
Toxicity, n (%)	4 (6%)	2 (3%)	0.68
Participant lost/default, n (%)	3 (5%)	0	0.24
Participant withdrew from study, n (%)	3 (5%)	1 (2%)	0.62
Pregnancy	1 (2%)	0	1.0
**Grade 3 or higher adverse event, n (%)**	**5 (8%)**	**6 (10%)**	**0.76**
**Descriptions of grade 3 or higher adverse events**			
Hepatotoxicity, n (%)	0	3 (5%)	0.11
Flu-like syndrome, n (%)	2 (3%)^1^	0	**0.50**
Change in visual refraction, n (%)	2 (3%)	1 (2%)	1.0
Rash, n (%)	1 (2%)^2^	0	1.0
Anemia, n (%)	0	1 (2%)	0.49
Nausea, n (%)	0	1 (2%)	0.49

### Population pharmacokinetics

PK parameter estimates for rifapentine and moxifloxacin are shown in Table 4. For rifapentine the median AUC_0-24_ was 313 (95% CI 103–823) mcg*h/mL and the median C_max_ was 12.1 (95% CI 6.3–21.6) mcg/mL. For moxifloxacin the median AUC_0-24_ was 28.0 (95% CI 19.9–64.2) mcg*h/mL and the median C_max_ was 2.5 (95% CI 1.6–3.0) mcg/mL.

**Table 4:** Average rifapentine and moxifloxacin pharmacokinetic parameter estimates in the current study compared to other recent studies using rifapentine alone or given together with moxifloxacin. Abbreviations: AUC_0-24_ = area under the concentration-time curve over 24 hours; C_max_ = maximum concentration; TBTC = Tuberculosis Trials Consortium.

	Rifapentine			Moxifloxacin		
	Dose	AUC_0-24_ (mcg*h/mL)	C_max_ (mcg/mL)	Dose	AUC_0-24_ (mcg*h/mL)	C_max_ (mcg/mL)
Current Study	300–450 mg daily	314	12.1	400 mg daily	28.0	2.5
TBTC [30,36]	450 mg daily	285	13.0	None	-	-
TBTC [30,36]	600 mg daily	320	15.5	None	-	-
Rifaquin [34]	None	-	-	400 mg once	50.8	3.8
Rifaquin [34]	1200 mg once weekly	Not reported	Not reported	400 mg once weekly	46.2	2.9
Rifaquin [34]	900 mg twice weekly	Not reported	Not reported	400 mg twice weekly	45.3	2.8
Healthy volunteers [35]	None	-	-	400 mg daily	41.9	4.0
Healthy volunteers [35]	900 mg three times weekly	410*	21.2	400 mg daily	34.4	3.3

* AUC_0-48_ (mcg*h/mL)

## Discussion

In this phase 2 tuberculosis clinical trial, an investigational intensive phase regimen comprised of daily low-dose rifapentine plus moxifloxacin in addition to isoniazid and pyrazinamide was not less bactericidal than the control regimen as assessed using sputum culture-based surrogate endpoints, and there was a possible trend towards superiority of the investigational regimen when MGIT liquid medium was used for endpoint measurement. However, in this study that was stopped prematurely for operational reasons, the small sample size limits the conclusions that can be made. Subsequent to the inception of this study, a multicenter dose-ranging phase 2 trial incorporating rifapentine doses up to 1500 mg daily showed a direct relationship between rifapentine exposure and bactericidal activity as assessed using sputum culture-based endpoints during the first 8 weeks of pulmonary tuberculosis treatment [30]. In a single-site study conducted in South Africa, rifapentine 450 mg daily was not as bactericidal as rifapentine 600 mg daily or rifampin 600 mg daily when administered for 8 weeks with isoniazid, pyrazinamide, and ethambutol [31]. Therefore, it is now clear that the daily rifapentine doses of 300 or 450 mg (approximately 7.5 mg/kg) used in the present study were suboptimal.

In our study, there are two potential explanations for the apparent retained bactericidal activity of the investigational regimen in the face of suboptimal rifamycin dose. The first is that giving rifapentine with food resulted in daily exposures that were similar to those achieved with higher doses without food in other studies (Table 4) and that daily dosing seven days per week improved bactericidal activity compared to weekday-only dosing. Secondly, moxifloxacin might have contributed substantially to the investigational regimen’s activity. Two previous phase 2 trials have demonstrated that the substitution of moxifloxacin for ethambutol improves 8-week culture conversion [22,23]. The phase 3 REMoxTB trial showed that the substitution of moxifloxacin for ethambutol produced a more rapid initial decline in sputum bacterial burden, but was not sufficiently sterilizing to successfully shorten the overall duration of treatment from six to four months while maintaining acceptable cure rates [24]. Whether the apparent enhanced bactericidal activity of moxifloxacin-containing regimens, when paired with optimized sterilizing activity conferred by optimized doses and exposures of rifamycins, is sufficient to meaningfully reduce treatment duration is an important unanswered question. The RIFAQUIN trial found that a continuation phase regimen comprised of once-weekly moxifloxacin 400 mg and rifapentine 1200 mg administered for four months was non-inferior to a conventional daily continuation phase regimen comprised of daily isoniazid and rifampin 600 mg, thereby providing some indirect support for this idea [32]. Moxifloxacin has been shown to accumulate in the cellular fraction of *M*. *tuberculosis* granulomas, and this property may be important for its observed clinical activity [33]. In our study, moxifloxacin exposures were significantly lower than in other studies in which rifapentine was co-administered less frequently (Table 4)—this is consistent with previous studies demonstrating that thrice-weekly rifapentine administration resulted in a 17% decrease in moxifloxacin exposure whereas once-weekly rifapentine administration resulted in a decrease in moxifloxacin exposure of only about 8% [34,35]. The relationship between moxifloxacin exposures and microbiologic activity, though, remains poorly defined, so it is unclear how potential reductions in moxifloxacin exposures resulting from daily co-treatment with rifapentine, a potent inducer of metabolizing enzymes, affected antibacterial activity.

With respect to tolerability of the investigational regimen, in our study a higher proportion of participants discontinued the investigational regimen compared to the standard control regimen for reasons other than microbiological ineligibility. The majority of excess discontinuations in the investigational arm were due to participant loss/default or participant withdrawal. Review of participants’ records did not uncover any trends or previously unrecognized toxicity, but nevertheless the reason for the higher discontinuation rate in the investigation arm is unclear. With respect to safety, the overall proportion of participants with clinically meaningful adverse events was low and compatible with other recent studies. Interestingly, in our study grade 3 or higher hepatotoxicity occurred only in the control arm (3 participants, vs. none in the investigational arm), and grade 3 or higher drug hypersensitivity events (i.e. rash or flu-like syndrome) occurred only in the investigational arm (3 participants, vs. none in the control arm). These trends have not been observed in other studies of mostly daily rifapentine for tuberculosis treatment [30,31,36]. However, in a recent large trial of treatments for latent tuberculosis infection, clinically significant systemic drug reactions including flu-like syndrome and cutaneous reactions were more common in participants receiving once-weekly rifapentine plus isoniazid than in participants receiving daily isoniazid [37].

There are several important limitations to our study. First and foremost, we were not able to accrue the target sample size within the funding period, thereby diminishing power to detect potential differences in the activity of the two regimens. Contributors to study delay included challenges with procurement of rifapentine as well as with procurement of MGIT liquid culture supplies and associated instrument repairs, challenges related to oversight by numerous regulatory bodies and ethics boards, and interruption of recruitment in the face of unforeseeable issues including military pacification of the favela in which one study clinic was based and closure of another study facility due to structural instability of an adjacent building. These challenges underscore the needs for continued research capacity-building in tuberculosis-endemic settings, and for innovations in tuberculosis trials design and surrogate markers to increase the efficiency with which potent, safe new regimens can be identified. Second, the ‘dual-substitution’ approach does not allow for definitive assessment of the contributions of each of the individual drug substitutions to antimicrobial activity and/or toxicity. While acknowledging this limitation, it is at the same time useful to reflect on the extended timeline required for assessment of single substitutions under the conventional drug development paradigm, versus the shorter timeline if the focus is on identifying *regimens* of interest. An additional limitation is that MGIT liquid cultures could not be performed for all participants. However, this is unlikely to be an important source of bias since interruption of MGIT cultures occurred during a discrete time period and affected both treatment arms.

## Supporting Information

S1 File Study Protocol. The study was conducted in accordance with the protocol.
(PDF)

S2 File CONSORT Checklist. CONSORT 2010 checklist of information to include when reporting a randomised trial.
(PDF)
