# Supplementary material for: A Phase 2 Randomized Trial of a Rifapentine plus Moxifloxacin-Based Regimen for Treatment of Pulmonary Tuberculosis
Source: PLoS One. 2016 May 9;11(5):e0154778. doi: 10.1371/journal.pone.0154778 (PMC4861335; doi:10.1371/journal.pone.0154778)
Supplement: S1 File — The study was conducted in accordance with the protocol. (PDF) [file pone.0154778.s001.pdf]

A Phase II Randomized, Open-Label Trial of a Rifapentine Plus Moxifloxacin-Based Regimen for Intensive Phase Treatment of Smear-Positive Pulmonary Tuberculosis

Principal Investigator: Susan E. Dorman, MD

Version Number: 10.0

7 September 2011

STATEMENT OF COMPLIANCE

This trial will be conducted with human subject's oversight from the following Institutional Review Boards (IRBs):

Johns Hopkins Medical Institutions (Federalwide Assurance [FWA] 00005752)

IRB of the Federal University of Rio de Janeiro (FWA 00000377)

IRB of the Municipal Health Secretary of Rio de Janeiro (FWA 00010761)

IRB of Oswaldo Cruz Foundation, Sergio Arouca School of Public Health (FWA 00000389)

National Commission on Research Ethics (CONEP), Brazil

The study will be carried out in accordance with Good Clinical Practice (GCP) as required by the following:

- United States (US) Code of Federal Regulations (CFR) applicable to clinical studies (45 CFR Part 46; 21 CFR Part 50, 21 CFR Part 56, and 21 CFR Part 312)
- ICH E6; 62 Federal Register 25691 (1997)
- National Institutes of Health (NIH) Clinical Terms of Award
- Brazilian National Concil of Health Resolutions 196/96 and 251/97.

All key personnel (all individuals responsible for the design and conduct of this study) have completed Human Subjects Protection Training.

SIGNATURE PAGE

The signature below constitutes the approval of this protocol and the attachments, and provides the necessary assurances that this trial will be conducted according to all stipulations of the protocol, including all statements regarding confidentiality, and according to local legal and regulatory requirements and applicable United States (US) and Brazil federal regulations and International Conference on Harmonisation (ICH) guidelines.

Principal Investigator
Signed: ______________________ Date: ____________

Susan E. Dorman, MD
Assistant Professor of Medicine and International Health
Johns Hopkins University

Co-Principal Investigator:
Signed: ______________________ Date: ____________

Richard E. Chaisson, MD
Professor of Medicine and International Health
Johns Hopkins University

Principal Investigator, Federal University of Rio de Janeiro:
Signed: ______________________ Date: ____________

Marcus B. Conde, MD
Associate Professor of Medicine
Federal University of Rio de Janeiro

Investigator, Municipal Health Secretary of Rio de Janeiro:
Signed: ______________________ Date: ____________

Solange Cavalcante, MD
Superintendent of the Primary Health Care Sub-Secretary
Health Secretariat of Rio de Janeiro city, Brazil

Investigator, Oswaldo Cruz Foundation,:
Signed: ______________________ Date: ____________

Margareth Dalcolmo, MD
Professor of Medicine
Oswaldo Cruz Foundation

TABLE OF CONTENTS

Page
Statement of Compliance i
Signature Page ii
Table of Contents iii
List of Abbreviations vi
Protocol Summary viii
1 Key Roles 1
2 Background Information and Scientific Rationale 5
2.1 Background Information 5
2.1.1 Tuberculosis as a Global Health Problem 5
2.1.2 Tuberculosis in Rio de Janeiro, Brazil 5
2.1.3 Need for New Treatment Regimens for Tuberculosis 5
2.1.4 Preclinical Studies of Activity of Moxifloxacin Against *Mycobacterium tuberculosis* 7
2.1.5 Clinical Experience with Fluoroquinolones for Tuberculosis Treatment 8
2.1.6 Preclinical Studies of Activity of Rifapentine Against *Mycobacterium tuberculosis* 10
2.1.7 Clinical Experience with Rifapentine for Tuberculosis Treatment 12
2.1.8 Preclinical Experience with Rifapentine and Moxifloxacin Combination Therapy for the Treatment of Tuberculosis 18
2.1.9 Clinical Experience with Rifapentine and Moxifloxacin Combination Therapy for the Treatment of Tuberculosis 19
2.1.10 Rationale for Selected Rifapentine Dose/Schedule 19
2.1.11 Study Drugs 21
2.2 Rationale 29
2.3 Potential Risks and Benefits 30
2.3.1 Potential Risks 30
2.3.2 Known Potential Benefits 32
3 Objectives 33
3.1 Study Objectives 33
3.2 Study Outcome Measures 33
3.2.1 Primary Outcome Measures 33
3.2.2 Secondary Outcome Measures 34
4 Study Design 35
5 Study Enrollment and Withdrawal 38
5.1 Subject Inclusion Criteria 39
5.2 Subject Exclusion Criteria 40
5.3 Treatment Assignment Procedures 41
5.3.1 Randomization Procedures 41
5.3.2 Reasons for Withdrawal 42
5.3.3 Handling of Withdrawals 42
5.3.4 Triggers for Review 43
6 Study Intervention/Investigational Product 44

6.1 Study Product Description (Moxifloxacin) 44
6.1.1 Acquisition 44
6.2 Study Product Description (Rifapentine) 44
6.2.1 Acquisition 45
6.2.2 Product Storage and Stability 45
6.3 Dosage, Preparation and Administration of Study Intervention/Investigational Product 45
6.4 Modification of Study Intervention/Investigational Product for a Participant 47
6.5 Accountability Procedures for the Study Intervention/Investigational Product(s) 47
6.6 Assessment of Subject Compliance with Study Intervention/Investigational Product 47
6.7 Concomitant Medications/Treatments 47
7 Study Schedule 49
7.1 Screening 49
7.2 Enrollment/Baseline 50
7.3 Follow-up 50
7.4 Final Study Visit 51
7.5 Early Termination Visit 51
7.6 Unscheduled Visit 51
8 Study Procedures/Evaluations 52
8.1 Clinical Evaluations 52
8.2 Laboratory Evaluations 55
8.2.1 Clinical Laboratory Evaluations 55
8.2.2 Special Assays or Procedures 59
8.2.3 Specimen Preparation, Handling, and Shipping 59
9 Assessment of Safety 60
9.1 Methods and Timing for Assessing, Recording, and Analyzing Safety Parameters 60
9.1.1 Adverse Events 60
9.1.2 Serious Adverse Events 62
9.1.3 Procedures to be Followed in the Event of Abnormal Laboratory Test Values or Abnormal Clinical Findings 63
9.2 Reporting Procedures 64
9.2.1 Serious Adverse Events 64
9.2.2 Regulatory Reporting for Studies Conducted Under DMID-Sponsored IND 65
9.2.3 Reporting of Pregnancy 65
9.3 Type and Duration of Follow-up of Subjects after Adverse Events 65
9.4 Halting Rules 65
9.5 Safety Oversight 66
10 Clinical Monitoring 67
10.1 Site Monitoring Plan 67
11 Statistical Considerations 68
11.1 Study Hypotheses 68
11.2 Sample Size Considerations 69
11.2.1 Safety Review 70

11.2.2 Efficacy Review 70
11.3 Final Analysis Plan 70
12 Source Documents and Access to Source Data/Documents 73
13 Quality Control and Quality Assurance 74
14 Ethics/Protection of Human Subjects 75
14.1 Ethical Standard 75
14.2 Institutional Review Board 75
14.3 Informed Consent Process 75
14.3.1 Informed Consent/Assent Process (in Case of a Minor) 76
14.4 Exclusion of Women, Minorities, and Children (Special Populations) 76
14.5 Subject Confidentiality 76
14.6 Study Discontinuation 76
14.7 Future Use of Stored Specimens 77
15 Data Handling and Record Keeping 78
15.1 Data Management Responsibilities 78
15.2 Data Capture Methods 79
15.3 Types of Data 79
15.4 Timing/Reports 79
15.5 Study Records Retention 79
15.6 Protocol Deviations 79
16 Publication Policy 80
17 Literature References 81
AppendiX: Schedule of events 88

LIST OF ABBREVIATIONS

AE	Adverse Event
AFB	Acid-fast Bacilli
AIDS	Acquired Immunodeficiency Syndrome
AST	Aspartate Aminotransferase
ATS	American Thoracic Society (guidelines)
AUC	Area Under the Plasma Concentration-Time Curve
BDMC	Biostatistics and Data Management Center
CDC	Centers for Disease Control and Prevention
CFR	Code of Federal Regulations
CFU	Colony-Forming Units
CRF	Case Report Form
DHHS	Department of Health and Human Services
DMID	Division of Microbiology and Infectious Diseases, NIAID, NIH, DHHS
DOT	Directly Observed Therapy
DSMB	Data and Safety Monitoring Board
eCRF	Electronic Case Report Form
ELISA	Enzyme-linked Immunosorbent Assay
FDA	Food and Drug Administration
FWA	Federalwide Assurance
GCP	Good Clinical Practice
HIV	Human Immunodeficiency Virus
HPZE	Isoniazid, Rifapentine, Pyrazinamide, Ethambutol
HRZE	Isoniazid, Rifampin, Pyrazinamide, Ethambutol
HS, HR, and S	Isoniazid plus Streptomycin, Isoniazid plus Rifampin, Streptomycin
ICH	International Conference on Harmonisation
ICMJE	International Committee of Medical Journal Editors
IDSA	Infectious Disease Society of America (guidelines)
IEC	Independent Ethics Committee
IND	Investigational New Drug Application
IRB	Institutional Review Board
MDR	Multi-Drug Resistant
MIC	Minimum Inhibitory Concentration
MPZE	Moxifloxacin, Rifapentine, Pyrazinamide, Ethambutol
MRZE	Moxifloxacin, Rifampin, Pyrazinamide, Ethambutol
NIAID	National Institute of Allergy and Infectious Diseases, NIH, DHHS
NIH	National Institutes of Health
OHRZ	Ofloxacin, Isoniazid, Rifampin, Pyrazinamide
PD	Pharmacodynamic
PI	Principal Investigator
PK	Pharmacokinetic

QA	Quality Assurance
QC	Quality Control
RH	Rifampin plus Isoniazid
RHZ	Rifampin, Isoniazid, and Pyrazinamide
RM	Rifampin and Moxifloxacin
RMZ	Rifampin, Moxifloxacin, and Pyrazinamide
SAE	Serious Adverse Event
TB	Tuberculosis
TBTC	Tuberculosis Trials Consortium
US	United States
WHO	World Health Organization

PROTOCOL SUMMARY

**Title:**	A Phase II Randomized, Open-Label Trial of a Rifapentine Plus Moxifloxacin-Based Regimen for Intensive Phase Treatment of Smear-Positive Pulmonary Tuberculosis
**Phase:**	2
**Population:**	216 adults (males and females) ≥ 18 years of age with respiratory smear-positive, culture-confirmed pulmonary tuberculosis (TB) in Rio de Janeiro, Brazil and Baltimore. HIV-positive individuals with CD4 ≥ 350 cells/cu mm, and HIV-negative individuals will be included.
**Number of Sites:**	Four sites: Hospital Universitario Clementino Fraga Filho of the Federal University of Rio de Janeiro, , Albert Sabin Health Post, Municipal Health Department of Rio de Janeiro, Helio Fraga Reference Center, Oswaldo Cruz Foundation, Rio de Janeiro and Johns Hopkins Medical Institution, Baltimore, Maryland.
**Study Duration:**	48 months
**Subject Participation Duration:**	Subject participation duration will be up to 38 weeks, with additional time for follow-up if needed.
**Description of Agent or Intervention:**	This trial will compare the antimicrobial activity and safety of a novel oral antimicrobial regimen to that of the standard regimen for the intensive phase treatment of pulmonary TB.<br>The experimental intensive phase regimen will consist of the following:<br>Two months of isoniazid, rifapentine, pyrazinamide and moxifloxacin (HPZM) administered once daily. Pyridoxine (vitamin B6) will be given with each dose of isoniazid.<br>The standard control intensive phase regimen will consist of the following:<br>Two months of isoniazid, rifampin, pyrazinamide, and ethambutol (HRZE) administered once daily. Pyridoxine (vitamin B6) will be given with each dose of isoniazid.<br>Following intensive phase therapy (the study phase), all patients will be treated with a non-experimental continuation phase regimen.

**Objectives:**	Primary:<br>• To compare, by treatment group, the proportions of patients with a negative sputum culture at the end of intensive phase therapy.<br>• To compare the safety and tolerability of the 2 intensive phase regimens.<br><br>Secondary:<br>• To compare the time to respiratory culture conversion of the 2 intensive phase regimens, using data from weekly cultures.<br>• To compare, by treatment group, the proportions of subjects who experience treatment failure.<br>• To compare, by HIV serostatus, a) the safety of the 2 intensive phase regimens, b) the proportions of patients with negative sputum cultures at the end of intensive phase therapy, and c) the time to culture conversion using data from weekly cultures.<br>• To compare, in subjects with versus without cavitation on baseline chest x-ray, the proportions of patients with negative sputum cultures at the end of intensive phase therapy.<br>• To store serum for future assessment of hypersensitivity to study drugs, should it occur.<br>• To identify, for rifapentine and moxifloxacin, the PK/PD parameters that correlate most strongly with TB treatment response as measured by 2-month culture conversion;<br>• To identify, for rifapentine and moxifloxacin, the PK/PD parameters that correlate most strongly with adverse events;<br>• To describe the pharmacokinetics of rifapentine administered at 450 mg daily in the context of isoniazid, pyrazinamide, and moxifloxacin when used to treat pulmonary TB.
**Description of Study Design:**	This will be a multi-center randomized, open-label clinical trial of 2 oral treatment regimens given for the first 8 weeks (intensive phase) of treatment of respiratory smear-positive, culture-confirmed pulmonary TB. Subjects will be adult males and females recruited from the Hospital Universitario Clementino Fraga Filho Hospital, of the Federal University of Rio de Janeiro, Brazil, the Albert Sabin Health Post, Municipal Health Department of Rio de Janeiro, Helio Fraga Reference Center, Oswaldo Cruz Foundation, Rio de Janeiro and the Johns Hopkins Medical Institution, Baltimore, Maryland.
**Estimated Time to Complete Enrollment:**	24 months

Schematic of Study Design:

Experimental Arm	Sample Size: 108	HPZM daily† for 8 weeks *followed by* continuation phase treatment with standard therapy for 18 or 30 weeks†
Control Arm	Sample Size: 108	HRZE daily† for 8 weeks *followed by* continuation phase treatment with standard therapy for 18 or 30 weeks†

H=isoniazid 200/300mg; R=rifampin450/600mg; Z=pyrazinamide 25mg/kg rounded to nearest 250mg; E=ethambutol 20mg/kg rounded to nearest 100mg; P=rifapentine 300/450mg; M=moxifloxacin 400mg

† pyridoxine (vitamin B6) 50 mg will be administered with each dose of isoniazid.

Randomization will be stratified by the presence of cavitation at baseline (time of diagnosis), since cavitation is associated with a decreased rate of 2-month culture conversion (67% versus 85% in Tuberculosis Trials Consortium Study 22) (The Tuberculosis Trials Consortium, 2002).

Drug susceptibility testing will be performed for all baseline *M. tuberculosis* isolates.

Following guidelines of the ATS/IDSA/CDC (Blumberg et al., 2003), duration of continuation phase TB treatment may be 30 weeks for patients having both cavitation (on initial chest x-ray) plus a positive sputum culture (for *M. tuberculosis*) at completion of intensive phase therapy. Duration of continuation phase will be 18 weeks for other patients. However, for any patient, duration of continuation phase may be extended at the discretion of the investigator.

Flow diagram:

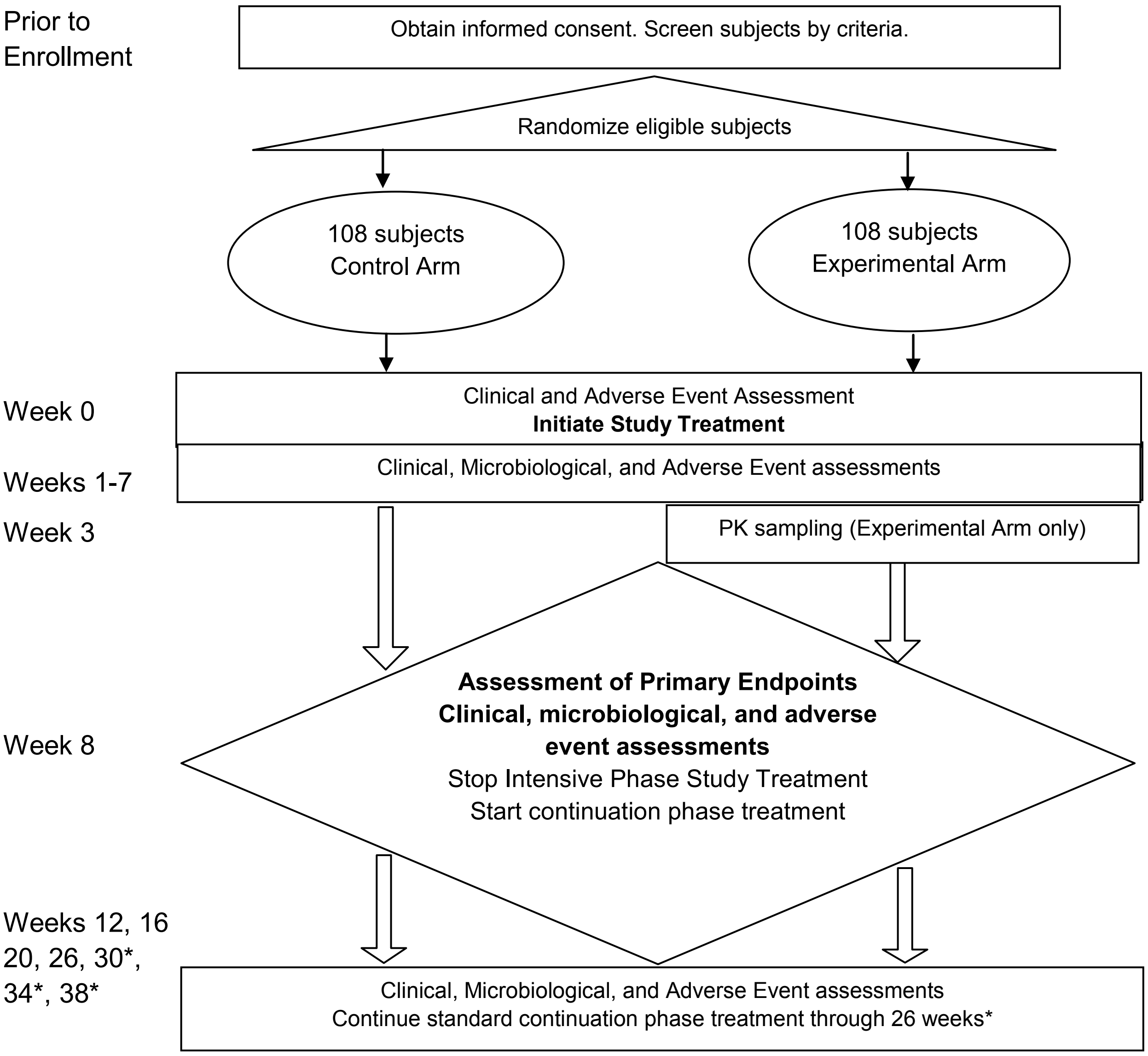

*For patients with cavitation on the baseline chest radiograph plus a positive respiratory culture (*for M. tuberculosis*) at completion of intensive phase treatment, standard continuation phase treatment will be extended through Study Week 38 (a total of 30 weeks of continuation phase treatment), with study visits (for clinical, microbiological, and adverse event assessments) at Weeks 30, 34, and 38.

1 KEY ROLES

Individuals:

Investigators:

Susan E Dorman, MD (Principal Investigator)
Assistant Professor of Medicine and International Health
Johns Hopkins University
1550 Orleans Street, Baltimore, MD 21231
Phone: 410-502-2717
Fax: 410-955-0740
Email: dsusan1@jhmi.edu

Richard E Chaisson, MD (Co-Principal Investigator)
Professor of Medicine, Epidemiology, and International Health
Johns Hopkins University
1550 Orleans Street, Baltimore, MD 21231
Phone: 410-955-1755
Fax: 410-955-0740
Email: rchaiss@jhmi.edu

Marcus B. Conde, MD, PhD (Rio de Janeiro Site Principal Investigator)
Associate Professor of Medicine
Hospital Universitario Clementino Fraga Filho
Federal University of Rio de Janeiro
Rua Professor Rodolpho Paulo Rocco, nº 255
s/n 4 andar SME da Pneumologia
Cidade Universitaria - Ilha do Fundao
Rio de Janeiro, Brazil CEP 21941.913
Phone: +55 (21) 2562 2426
Fax: +55 (21) 2562 2853
Email: marcusconde@hucff.ufrj.br

Co-Investigators:

Fernanda Mello, MD, PhD
Hospital Universitario Clementino Fraga Filho
Federal University of Rio de Janeiro
Rua Professor Rodolpho Paulo Rocco, nº 255
s/n 4 andar SME da Pneumologia
Cidade Universitaria - Ilha do Fundao
Rio de Janeiro, Brazil CEP 21941.913
Phone: +55 (21) 9624 0864
Fax: +55 (21) 2562 2853
Email: fcqmello@uninet.com.br

Solange Cesar Cavalcante, MD (Municipal Health Secretary site Prinicipal Investigator)
Municipal Health Secretary
Transmissable Diseases Division
Avenida Niemeyer 776, 14 andar
Sala 1405, Cidade Nova
Rio de Janeiro, RJ 22450-002
Phone : +55 (21) 3322 0096
Email : scavalcante@rio.rj.gov.br

Margareth Pretti Dalcolmo, MD (FioCruz site Prinicipal Investigator)
Centro de Referência Professor Hélio Fraga - ENSP - FIOCRUZ
Estrada de Curicica, Nº 2.000 - Curicica - Jacarepaguá
Rio de Janeiro - RJ
CEP: 22.780-192
Phone: +55 (21) 2448-6812
Fax: +55 (21) 2441- 0799
Email: mpdalcolmo@hotmail.com

Kelley Dooley, MD
The Johns Hopkins University School of Medicine
Department of Clinical Pharmacology
600 North Wolfe Street, Osler 527
Baltimore, MD 21287
Phone: 410-955-3100
Fax: 410-614-9978
E-mail: kdooley1@jhmi.edu

Anne Efron, MSN, MPH
Johns Hopkins University School of Medicine
1820 Lancaster Street, Suite 300
Baltimore, MD 21231
Phone: 410-502-2803
Fax: 443-287-7955
Email: aefron@jhmi.edu

Silvia Cohn, MPH
Ataulfo de Paiva 50 Bloco B1 Apto 1306
Leblon, Rio de Janeiro
RJ, Brazil CEP 22440-033
Phone: +55 (21) 3322 0096
Email : scohn5@jhmi.edu

Biostatistician
Lawrence Moulton, PhD
Professor of International Health
Johns Hopkins Bloomberg School of Public Health
615 North Wolfe Street, E5519
Baltimore, MD 21205
Phone: 410-955-6370
Email: lmoulton@jhsph.edu

Institutions:

Sponsors: National Institutes of Allergy and Infectious Diseases
National Institutes of Health
Tuberculosis Trials Consortium, Centers for Disease Control

Coordinating site: Johns Hopkins University Medical Institutions
Center for Tuberculosis Research
1550 Orleans Street
Baltimore, MD 21231
Contact Person: Susan E. Dorman, MD
Phone: 410-502-2717
Fax: 410-955-0740
Email: dsusan1@jhmi.edu

Study Sites: Hospital Universitario Clementino Fraga Filho
Federal University of Rio de Janeiro
Rua Professor Rodolpho Paulo Rocco, nº 255
Cidade Universitaria - Ilha do Fundao
Rio de Janeiro, Brazil CEP 21941.913
Contact Person: Marcus Conde, MD
Phone:+55 (21) 2562 2426
Fax:+55 (21) 2562 2853
Email:marcusconde@hucff.ufrj.br

Posto de Saude Albert Sabin
c/o Dra. Solange Cavalcante
Avenida Niemeyer 776 sala 1405
Cidade Nova
Rio de Janeiro, RJ 20211-110
Brasil
Contact Person : Solange Cavalcante, MD
Phone : +55 (21) 3322 0096
Email : scavalcante@rio.rj.gov.br

Centro de Referência Professor Hélio Fraga - ENSP - FIOCRUZ
Endereço: Estrada de Curicica, Nº 2.000 - Curicica - Jacarepaguá
Rio de Janeiro - RJ
CEP: 22.780-192
Contact Person: Margareth Pretti Dalcolmo, MD
Telefones: 2448-6816 / 6817 / 6818
FAX: 2441- 0799

Johns Hopkins University Medical Institutions
Center for Tuberculosis Research
1550 Orleans Street
Baltimore, MD 21231
Contact Person: Susan E. Dorman, MD
Phone: 410-502-2717
Fax: 410-955-0740
Email: dsusan1@jhmi.edu

2 BACKGROUND INFORMATION AND SCIENTIFIC RATIONALE

2.1 Background Information

2.1.1 Tuberculosis as a Global Health Problem

Tuberculosis (TB) is one of the most important global health problems. According to recent estimates from the World Health Organization (WHO), 9 million new cases and 2 million deaths from TB occurred in 2004, making TB the second leading cause of death from an infectious pathogen, exceeded only by human immunodeficiency virus (HIV)/acquired immunodeficiency syndrome (AIDS)(World Health Organization, 2006). The vast majority of TB cases and TB deaths are in developing countries. The spread of HIV has fueled the TB epidemic, and TB is currently the leading cause of death among patients infected with HIV (Corbett et al., 2003). Tuberculosis predominantly affects young adults in their most productive years of life and has substantial impact on economic development.

2.1.2 Tuberculosis in Rio de Janeiro, Brazil

Brazil has the highest number of TB cases in Latin America, with an overall TB incidence of 60/100,000 population in 2004, and the 16th highest absolute number of TB cases of all countries in the world in 2004. Approximately 10,000 new TB cases per year have been reported to the City of Rio de Janeiro Health Secretariat for an incidence of 175 cases per 100,000 residents. The incidence of TB in Rio has increased in the past 5 years as a result of crowding in urban slums, a growing HIV epidemic and inadequate diagnostic and treatment resources. This setting, in which the TB disease burden and the need for better TB treatments are high, provides an important opportunity to evaluate a new drug treatment for TB. Our group has extensive experience in conducting TB clinical trials and has a strong infrastructure for carrying out the proposed research in Rio de Janeiro.

2.1.3 Need for New Treatment Regimens for Tuberculosis

Although effective therapy for drug susceptible *Mycobacterium tuberculosis* is available, TB continues to cause significant morbidity and mortality worldwide, and rates of multi-drug resistant (MDR) TB cases are on the rise. A major obstacle to the control of TB is poor adherence with lengthy (usually 6 months) and complicated treatment regimens. Incomplete TB treatment can lead to serious consequences such as increased morbidity and mortality, prolonged infectiousness and transmission in the community, and the development of drug resistance. The use of directly observed therapy (DOT) can improve patient adherence and reduce the emergence of resistant microorganisms, but is logistically difficult and expensive to implement (McDonald et al. 1982). The development of novel treatment strategies with more potent antimycobacterial activity could lead to shorter and simpler regimens. A TB treatment regimen that allowed treatment duration to be meaningfully decreased would have important public health implications by facilitating DOT, increasing cure rates, potentially reducing transmission, and potentially preventing emergence of MDR TB.

Only 1 drug, rifapentine, has been approved for treatment of TB in the past 30 years. Rifapentine is a rifamycin derivative with excellent activity against *M tuberculosis* (Dickinson et al. 1987; Ji et al. 1993). The long serum half-life of rifapentine compared to rifampin (10-15 hours vs 2-3 hours) renders rifapentine a promising role in intermittent dosing regimens.

Fluoroquinolone antibiotics are active against most strains of *M tuberculosis* and have gained general acceptance for the treatment of MDR TB. Recent experimental and clinical data suggests that certain fluoroquinolones may be potent sterilizing agents that could shorten regimens for the treatment of active TB. The relatively long serum half-life of moxifloxacin (plasma half-life of approximately 12 hours), makes it particularly attractive for the treatment of TB. As described in subsequent protocol sections, combination of a long-acting fluoroquinolone and rifapentine may allow for a less frequent dosing schedule and may be more potent than the standard regimen.

Because combination therapy is mandatory for the treatment of TB, approval of new drugs requires 1 of 2 approaches: either substitution of a candidate drug for one of the current first line drugs, or addition of a new drug to an approved regimen. The experimental treatment regimen consists of isoniazid, rifapentine, pyrazinamide, and moxifloxacin (HPZM), where moxifloxacin is substituted for ethambutol, and rifapentine is substituted for rifampin. As described below, in the murine model, these regimens have much better sterilizing activity compared to standard daily therapy with isoniazid, rifampin, pyrazinamide, and ethambutol (HRZE).

The murine model of TB has been used for more than 50 years for the development and evaluation of new antituberculosis drugs and regimens (Veziris et al. 2005). Importantly, the mouse model of TB treatment has been shown to recapitulate human TB treatment with regard to treatment-shortening effects of rifampin and pyrazinamide. Mice can be infected via aerosol in an inhalation exposure system. Two to three weeks after infection, bacillary burden can reach 10^7-10^8 cfu -- similar to the estimated bacillary burden in human TB pulmonary cavities. Treatment efficacy in the mouse model of active TB is typically assessed by lung and spleen bacterial colony forming unit (cfu) counts that can be measured at various intervals during treatment; relapse can be assessed by lung and/or spleen cfu counts assayed 3-6 months after treatment is stopped. In the mouse model, the standard 6-month rifampin plus isoniazid plus pyrazinamide (RHZ)-based regimen cures mice in 6 months, but is followed by relapse rates of 0-10%. This represents the murine TB treatment “reference standard” against which new treatments are compared.

With respect to clinical trials of TB treatment, the most important clinical indicator of outcome is the proportion of subjects with TB relapse two years after completion of TB therapy. However, because relapse rates are typically 3-5% in the setting of directly observed therapy with current regimens, such studies require several thousand patients per study arm, and several years for study completion. These daunting requirements have slowed the pace of TB clinical research, and led to identification of surrogate markers for TB treatment efficacy (Wallis et al., 2005). The surrogate marker most widely accepted and best validated is the proportion of patients who convert sputum mycobacterial cultures to negative at completion of two months of TB treatment. Mitchison performed a retrospective analysis of 8 large, controlled clinical TB trials (Mitchison DA, 1993). The proportion of culture negative patients at 2 months was inversely related to two-year relapse rates. Moreover, proportion of culture negative patients at 2 months was inversely

related to duration of treatment required for stable cure without relapse. For example, the addition of pyrazinamide to regimens containing at isoniazid, rifampin, and streptomycin was associated with an average 10% increase in sputum culture conversion at two months; the addition of pyrazinamide allowed overall treatment shortening from 9 months to 6 months (Wallis et al., 2005).

2.1.4 Preclinical Studies of Activity of Moxifloxacin Against *Mycobacterium tuberculosis*

Moxifloxacin has excellent activity against *M tuberculosis* both in vitro and in animal models. In a limited study using the agar proportion method on 7H10 medium, Woodcock et al. (1997) found that the minimum inhibitory concentration (MIC) for moxifloxacin ranged from 0.12 to 0.5 µg/ml for 4 clinical isolates of *M tuberculosis*, 3 of which were drug-resistant (to isoniazid, rifampin, and/or streptomycin). Gillespie and Billington (1999) determined the MICs for 19 strains of *M tuberculosis* to moxifloxacin, ciprofloxacin, levofloxacin, and sparfloxacin using the proportion method in 7H10 agar Moxifloxacin was the most active of the drugs tested. Rodriguez and others (2001) conducted a similar evaluation against 55 clinical TB isolates from untreated patients with similar findings. In another recent study from Italy, the activity of moxifloxacin was tested against 86 *M tuberculosis* strains including 13 resistant and 4 MDR strains. All but 2 strains were susceptible to moxifloxacin at 0.5 µg/ml (Tortoli et al. 2004).

Ji and others (1998) evaluated the in vitro and in vivo activities of moxifloxacin, clinafloxacin, and sparfloxacin. For sparfloxacin and moxifloxacin, the MIC_{90} (determined on 7H11 medium by the proportion method) were similar and slightly lower than that for clinafloxacin. In the mouse study, groups of 30 female Swiss mice each were infected by the intravenous (IV) route with 6.2×10^6 cfu of *M tuberculosis* H37Rv strain and treated 6 times weekly for 4 weeks with various drug dosages beginning 1 day after infection. Moxifloxacin, at the 100 mg/kg dosage, was the most active of the 3 fluoroquinolones tested, and had activity comparable to that of isoniazid. This was the first study to demonstrate the significant activity of moxifloxacin in an animal model.

For moxifloxacin, 100mg/kg twice daily in mice provides an AUC similar to that of 400mg daily in humans (47.2 vs. 45.5 mcg*h/mL), but the C_{max} values differ (14.1 vs. 4.98 mcg/mL, respectively) (Rosenthal et al. 2005).

In mice infected with a sublethal inoculum of *M. tuberculosis* and then treated with moxifloxacin at 100mg/kg/day for 8 weeks, the lung log_{10}cfu counts at the end of treatment were significantly lower than those for the untreated control group (0.6 vs 5.6) and comparable to cfu in mice treated with isoniazid alone (Miyazaki et al. 1999).

In an important study by Nuermberger et al (2004a, 2004b), a regimen in which moxifloxacin was substituted for isoniazid and given in combination with rifampin and pyrazinamide, greatly reduced the time to culture conversion in murine TB, and lead to stable cure without relapse after only four months of therapy. Specifically, the mean log_{10}cfus of *M. tuberculosis* from lung homogenates after 2 months of therapy were 3.36 +/- 0.32 vs. 0.90 +/- 0.58, respectively, in the isoniazid- and in the moxifloxacin-based regimens (Figure 1). At 4 months, the cfu values were 0.39 +/- 0.32 for the isoniazid group compared with 0 for the moxifloxacin group. Mice were assessed for relapse after treatment with isoniazid- or moxifloxacin-based combination therapy

for 3, 4, 5, or 6 months; there were no relapses in 12 mice treated for 4 or more months with moxifloxacin-based therapy, whereas in the isoniazid treatment group, 5 of 12 mice relapsed after 4 months of treatment, 1 of 16 relapsed after 5 months of treatment, and none of 12 relapsed after 6 months of TB treatment.

Taken together, moxifloxacin studies in the mouse model of TB indicate that the sterilizing effect of standard TB therapy could be greatly improved by substitution of moxifloxacin for isoniazid.

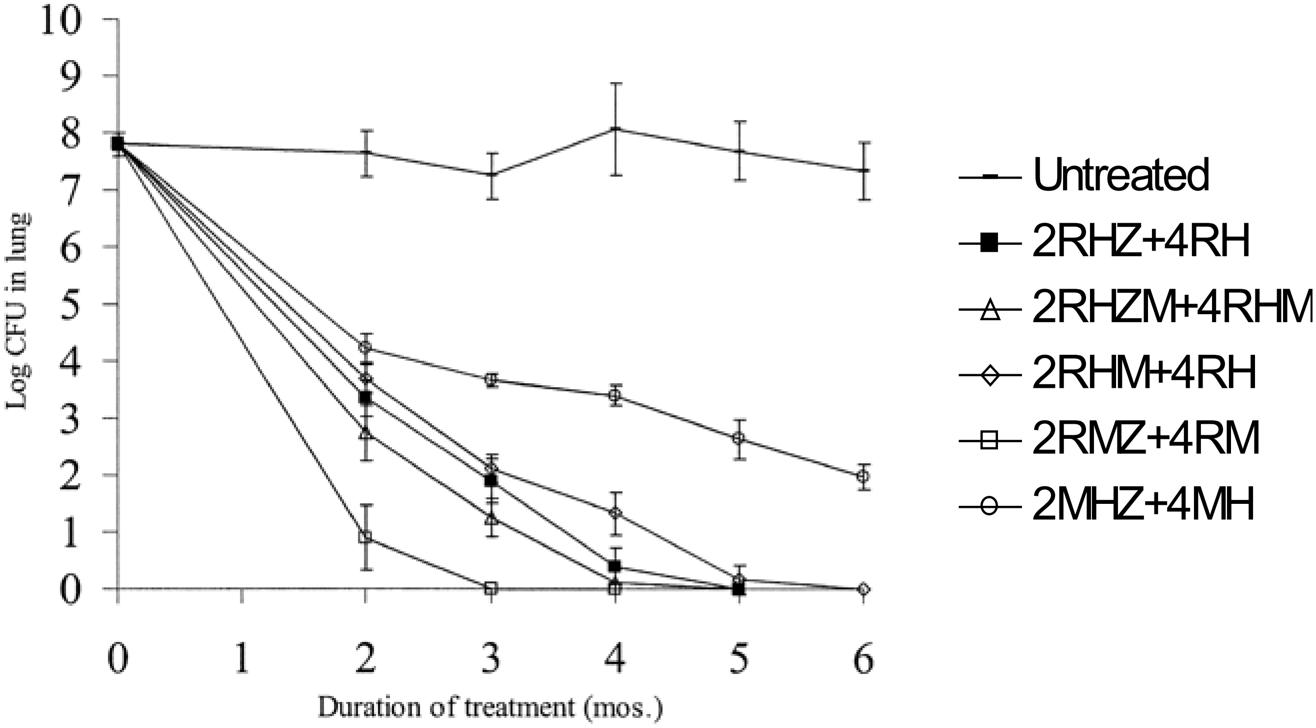

Figure 1. Lung colony forming unit counts during combination therapy with moxifloxacin-containing regimens. R=rifampin 10 mg/kg; H=isoniazid 25 mg/kg; Z=pyrazinamide 150 mg/kg; M=moxifloxacin 100 mg/kg.

2.1.5 Clinical Experience with Fluoroquinolones for Tuberculosis Treatment

The early bactericidal activity (EBA) of moxifloxacin for the treatment of pulmonary TB is excellent. In one EBA study, after 5 days of monotherapy with either moxifloxacin or isoniazid, the EBA for moxifloxacin was 0.275 $\log_{10}$ cfu/mL of sputum per day, and the EBA for isoniazid was 0.209 (Pletz et al. 2004). Johnson et al (2006) assessed the decline in bacilli during the first two days (days 0-2) and during days 2-7 of monotherapy in adults with untreated newly diagnosed pulmonary TB; 10 patients per drug arm were studied. The EBA (0-2) of isoniazid (0.67 $\log_{10}$cfu/ml/d) was greater than that of moxifloxacin (0.33 $\log_{10}$cfu/ml/d), but the extended mean EBA (2-7) for moxifloxacin (0.17 $\log_{10}$cfu/ml/d) was greater than that for isoniazid (0.08 $\log_{10}$cfu/ml/d).

Valerio et al (2003) conducted an observational study of the tolerability of moxifloxacin, given for 6 months in combination with isoniazid and rifampin, for treatment of active TB in individuals not eligible for standard TB treatment. In the moxifloxacin treated patients, results of chemical analyses, which included complete blood counts and comprehensive metabolic panels, did not

change over the 6 month treatment course. Information about other adverse events was not reported.

There have been two phase 2 clinical trials of moxifloxacin during intensive phase treatment of pulmonary TB. Chaisson and colleagues conducted an FDA Office of Orphan Products sponsored study entitled “Phase II randomized trial of a moxifloxacin-containing regimen for treatment of smear-positive pulmonary tuberculosis in adults with and without HIV infection”. This was a single center, randomized double-blind trial comparing the microbiologic and clinical effects of induction therapy of two daily regimens in Rio de Janeiro Brazil. In the experimental regimen, ethambutol was replaced by moxifloxacin during the intensive phase of TB treatment. The treatment regimens were a) “MOX”: INH 300 mg / RIF 600 mg / PZA 20 mg/kg / MOX 400 mg / EMB placebo once daily for 8 weeks, OR b) “EMB”: INH 300 mg / RIF 600 mg / PZA 20 mg/kg / EMB 15-20 mg/kg / MOX placebo once daily for 8 weeks. Following the 8 weeks of study phase treatment, all participants will continue treatment with INH 900 mg / RIF 600 mg twice weekly for an additional 16 weeks. The primary objectives were to compare the proportion of patients with sterile sputum cultures after 8 weeks of treatment with the two regimens, and to assess the proportion of patients with Grade 3 or 4 adverse reactions attributable to study medications after an 8-week induction regimen. The 8-week sputum culture conversion rate was 85% in the MOX arm vs. 68% in the EMB arm (p=0.02). Rates of conversion were higher with MOX from Week 1 onwards. Patients on MOX converted cultures to negative more rapidly (median – 36 days vs. 42 days for EMB, p=0.03). Toxicity in the two treatment arms was similar.

The CDC Tuberculosis Trials Consortium conducted “Study 27”, a placebo-controlled multicenter trial that compared the antimicrobial activity and tolerability of moxifloxacin versus ethambutol, in combination with isoniazid, rifampin and pyrazinamide, during the intensive phase of treatment for pulmonary TB (Burman et al, 2006). There was no difference in proportions of patients having negative cultures at completion of 2 months of treatment (99/139, 71% in the moxifloxacin arm versus 98/138, 71% in the ethambutol arm, p = 0.97). However, patients receiving moxifloxacin more often had negative cultures after 4 weeks of treatment. Importantly, lack of standardization in culture methods within and across sites may have contributed to wide variability in culture conversion across sites and have reduced the ability to detect a difference between regimens. Regarding safety and toxicity, similar proportions of patients randomized to moxifloxacin and ethambutol completed their assigned regimen (88% [145/165] and 89% [148/167], respectively, p = 0.83). Rates of SAEs were also similar (6% [10/167] versus 5% [8/165], p = 0.81 for moxifloxacin and ethambutol groups, respectively), and most SAEs were hospitalizations thought to be unrelated to study treatment; there was only one SAE attributed to study therapy, and this occurred in the ethambutol group. There was one death during the first two months of treatment – this was in the moxifloxacin group and was thought to be caused by pulmonary embolism, unrelated to TB therapy. Patients in the moxifloxacin group more often reported nausea (21.6% versus 9.1%, p = 0.002), but this seldom necessitated temporary or permanent discontinuation of study therapy. Joint pain was slightly more frequent in patients who received moxifloxacin (34% [57/167] versus 27% [44/165], p=0.15), but no cases of tendonopathy or arthritis were recognized. While visual changes were reported in both study groups, no cases of retinitis or optic neuritis were diagnosed.

Thus, moxifloxacin holds promise as an antituberculosis drug because of its potent early bactericidal activity, its superior sterilizing activity when combined with rifampin and pyrazinamide (i.e., substituted for isoniazid) in murine models, its tolerability, and its potential for use in both drug-susceptible and drug-resistant TB.

2.1.6 Preclinical Studies of Activity of Rifapentine Against *Mycobacterium tuberculosis*

Rifamycins are the key drugs in modern “short-course” TB chemotherapy of 6 months duration. Rifamycins, including rifampin, rifabutin, and rifapentine, have concentration-dependent activity against *M. tuberculosis*. Rifapentine is a rifamycin derivative with excellent activity against *M. tuberculosis*. For rifapentine, the minimum inhibitory concentration $(MIC)_{50}$ and MIC_{90} are one- to two-fold dilutions lower than those of rifampin (for the 7H10 agar system, rifapentine’s MIC_{50} and MIC_{90} are 0.125 and 0.25, compared with 0.5 and 1.0 for rifampin) (Bemer-Melchior et al., 2000). In addition, rifapentine’s half-life ($t_{1/2}$) is five times longer than that of rifampin (14-18 hours vs. 2-5 hours). Rifapentine’s low MIC and long half-life make it an attractive candidate for use in intermittently dosed (ie less frequently than daily) TB treatment regimens. (McDonald et al. 1982; Cohn et al. 1990) Rifapentine, at a dose of 600 mg twice-weekly, was approved by the Federal Drug Administration (FDA) in June of 1998 for the treatment of pulmonary TB (see below).

The efficacy of rifapentine for the treatment of TB was first evaluated in the mouse model, and studies have evaluated its efficacy in the intensive phase as well as the continuation phases of TB treatment.

Rifapentine’s pharmacokinetics are similar in mice and humans. A dose of 10 mg/kg in mice results in similar AUC and C_{max} as a dose of 600 mg (approximately 10 mg/kg) in humans; doses of 15 mg/kg in mice and 900 mg (approximately 15 mg/kg) in humans also have comparable pharmacokinetics (Rosenthal et al. 2005).

In murine models, intermittent rifapentine-containing regimens have been effective for the treatment of TB:

Daniel et al (2000) used a murine model of active TB to evaluate the activity of once weekly rifapentine (10 mg/kg/dose) during the continuation phase of TB treatment. Mice were treated with 2 months of RHZ, then were treated either with 4 months of daily rifampin plus isoniazid (RH) or 4 months of once-weekly rifapentine (10 mg/kg/dose) plus isoniazid (PH). The regimens were equally effective, as measured by lung and spleen cfu at 6 months – all mice in both groups were culture negative.

Importantly, in further murine experiments, Daniel et al (2000) demonstrated that increasing the dose of rifapentine from 10 to 15 mg/kg once weekly increased the anti-TB activity of the regimen, a finding consistent with concentration-dependent activity of rifapentine.

Rosenthal et al (2005) further assessed the activity of once-weekly rifapentine (15 mg/kg/dose) during continuation phase of TB treatment in mice. A continuation phase regimen of once-weekly rifapentine (15 mg/kg/dose) plus isoniazid was at least as effective as a continuation

phase regimen of twice weekly rifampin plus isoniazid, but not as effective as daily rifampin plus isoniazid when stable cure (as assessed by culture negative lungs three months after completion of treatment) was used as the endpoint. Specifically, after 6 months of therapy and 3 months of follow-up, the proportion of mice that were lung culture positive was 75% (18 of 24) in the once weekly rifapentine plus isoniazid group, 96% (23 of 24) in the twice weekly rifampin plus isoniazid group, and 25% (6 of 24) in the daily rifampin plus isoniazid group.

Subsequent murine experiments by Rosenthal et al (2006) explored the activity and pharmacokinetics of intensive phase regimens containing rifapentine administered on a twice-weekly schedule at doses of 15 or 20 mg/kg/dose. The rifamycin exposure, as defined by the weekly free-drug AUC:MIC or time above MIC, was much higher after administration of twice-weekly rifapentine (15 mg/kg/dose) as compared with daily rifampin (10 mg/kg/dose). Specifically, AUC:MIC ratios were 531 for rifapentine versus 405 for rifampin, and time per week above MIC values were 168 hours for rifapentine (100% of time) versus 80 hours for rifampin. After two months of intensive phase therapy, twice-weekly therapy with rifapentine (15 mg/kg/dose), isoniazid, and pyrazinamide (PHZ) was significantly more active than twice weekly RHZ, and had similar activity to daily RHZ, with lung cfu values of 2.37, 3.61, and 2.56, respectively. Moreover, stable cure was achieved after only 4 months of a twice weekly rifapentine (15 mg/kg/dose) plus isoniazid-based regimen, but only after 6 months of a daily rifampin plus isoniazid-based regimen.

Rosenthal has subsequently shown that in the murine model of TB treatment once-daily rifapentine administered during intensive phase has very potent antimycobacterial activity that results in durable cure after only 3 months of total treatment when combined with moxifloxacin (Rosenthal et al, 2007). After aerosol infection, mice achieved a bacillary burden of 7.45 $\log_{10}$ cfu per lung. Treatment with a standard regimen of daily rifampin (10 mg/kg) plus isoniazid and pyrazinamide resulted in a decrease in bacillary burden of ~3.5 logs at completion of 6 weeks of treatment. However, as shown in Figure 1, treatment with a regimen of daily rifapentine (10 mg/kg) plus isoniazid and pyrazinamide resulted in a decrease of ~6.5 $\log_{10}$ cfu ($p<0.05$ compared with the standard regimen). Furthermore, after 10 weeks of treatment followed by 3 months without treatment, 100% (15/15) of mice treated with the standard regimen had bacteriological relapse, compared to 13% (2/15) mice treated with the rifapentine-containing regimen ($p=0.01$, Table 1). There was a dose response relationship for rifapentine with respect to relapse rates (Table 2) and bactericidal activity as measured by cfu (not shown). Treatment 5 or 7 days of the week was more potent than intermittent treatment, and a dose of 10 mg/kg given 5 days per week was more potent than a dose of 7.5 mg/kg given 5 days per week.

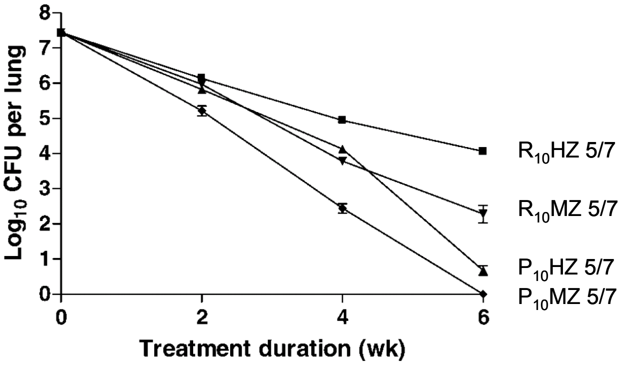

Figure 2. Bactericidal activity of intensive phase treatment regimens in *M. tuberculosis*-infected mice.

Regimen	Proportion with relapse (%)
$R_{10}HZ$ 5/7	15 / 15 (100)
$R_{10}MZ$ 5/7	8 / 15 (53)
$P_{10}HZ$ 5/7	2 / 15 (13)

Table 1. Culture-positive relapse rates after treatment for 10 weeks and then no treatment for 3 months.

Treatment Group	Treatment Period after which Relapse Rates were Determined			
	2 months	3 months	4 months	6 months
$R_{10}HZ$ 5/7	Not done	Not done	90% (18/20)	0% (0/20)
$P_{15}MZ$ 2/7	Not done	10% (2/20)	0% (0/20)	
$P_{15}MZ$ 3/7	95% (19/20)	0% (0/20)	--	--
$P_{7.5}MZ$ 5/7	60% (12/20)	5% (1/20)	--	--
$P_{10}MZ$ (5/7)	35% (7/20)	0% (0/20)	--	--
$P_{10}MZ$ (7/7)	20% (4/20)	--	--	--

Table 2. Culture-positive relapse rates after treatment for the specified duration followed by no treatment for 3 months.

Thus, murine studies indicate that rifapentine administered daily during combination intensive phase treatment has potent antimycobacterial activity that is associated with ability to achieve durable cure without relapse after approximately 3 months of total treatment.

Furthermore, in the murine model, a regimen containing daily rifapentine at 7.5 mg/kg, in combination with moxifloxacin, results in more rapid microbial sterilization than the standard daily rifampin-based regimen, and durable cure after approximately 3 months.

2.1.7 Clinical Experience with Rifapentine for Tuberculosis Treatment

Rifapentine (Priftin), at a dose of 600 mg twice-weekly, was approved by the U.S. Food and Drug Administration (FDA) in June of 1998 for the treatment of pulmonary TB after a prospective, open-label, industry-sponsored, randomized phase III trial ("Clinical Study 008") (Priftin package insert). This trial compared 2 months of daily isoniazid, rifampin, pyrazinamide, plus ethambutol (HRZE) followed by 4 months of twice weekly HR, with 2 months of daily HZE plus twice weekly rifapentine (600 mg/kg/dose) followed by 4 months of once weekly HP. The percentage of patients converting their sputum culture to negative was slightly higher in the rifapentine group (87%, 248/286) compared with the standard rifampin group (80%, 226/283). However, the proportion of patients with relapsed TB during 24 month follow-up was higher in the rifapentine group (12%, 29/248) than in the standard rifampin group (7%, 15/226). There were 11 deaths in each group, none of which were attributed to study medications. In the study, 11/361 (3.0%) of rifapentine recipients discontinued study treatment due to an adverse event, compared with 18/361 (5.0%) in the rifampin group. Hepatitis occurred in fewer than 1% of rifapentine recipients. The most common treatment-related adverse events in the rifapentine group were hyperuricemia (21.6%, and attributed to pyrazinamide), elevated liver transaminases (5.0%), and neutropenia (5.0%); these percentages were similar to those in the rifampin group.

A subsequent randomized, prospective study further evaluated the role of rifapentine in continuation phase therapy of pulmonary TB (Benator et al., 2002). After completion of two months of standard intensive phase TB treatment, patients were randomized to receive either once weekly rifapentine (600 mg, approximately 10 mg/kg/dose) plus isoniazid, or twice weekly rifampin (600 mg/dose) plus isoniazid. Crude rates of treatment failure or relapse were 46/502 (9.2%) in the rifapentine group, and 28/502 (5.6%) in the rifampin group (relative risk 1.64, 95%

CI 1.04-2.58, p=0.04). Of the 502 patients enrolled per group, similar proportions in each group died (5% in the rifapentine group versus 6% in the rifampin group) and no deaths were attributed to study medicines. There were no differences between treatment groups in frequency of grade 4 adverse events (44 events in the rifapentine group versus 72 events in the rifampin group), grade 3 or 4 adverse events attributed to study treatment (24 events in the rifapentine group versus 25 events in the rifampin group), or grade 3 or 4 hepatotoxicity (15 events in the rifapentine group versus 24 events in the rifampin group). Importantly, among HIV-positive participants in the rifapentine group, 4 of 5 relapses were with strains that had acquired rifamycin monoresistance during treatment (Vernon et al., 1999). Development of acquired rifamycin monoresistance was attributed to the low isoniazid concentrations present in the setting of once-weekly isoniazid administration, since this drug has a short half-life (Weiner et al., 2003).

Subsequent early bactericidal activity (EBA) studies of rifapentine in pulmonary TB patients indicate that 600 mg is not the optimal rifapentine dose. Sirgel et al (2000) evaluated the EBA over 5 days in pulmonary TB patients who received single rifapentine doses of 300, 600, 900 or 1200 mg. The EBA_{0-5} of rifampin dosed at 600mg daily was 0.226. In patients receiving one dose of rifapentine, the EBAs for 600, 900, and 1200mg were 0.193, 0.251, and 0.248, respectively. A dose response curve using these data showed the maximum effect for rifapentine at a dose of 900 to 1200 mg (15-20 mg/kg).
Bock et al (2002) evaluated the tolerability of higher doses of rifapentine given once weekly in combination with isoniazid during the continuation phase of pulmonary TB treatment. In this prospective, double-blind, randomized trial, 150 HIV-negative patients were randomized to one of three doses of once-weekly rifapentine: 600 mg, 900 mg, or 1200 mg. Treatment was discontinued in 3 of 52 (6%), 2 of 51 (4%), and 3 of 47 (6%) in the rifapentine 600-, 900- and 1200-mg groups, respectively. Only one discontinuation, in the rifapentine 1200 mg group, was due to an adverse event possibly related to study therapy (first trimester spontaneous abortion). There was a borderline statistically significant trend toward adverse events possibly due to study treatment across the three doses when the spontaneous abortion was included (p = 0.051), but there was no significant trend when the spontaneous abortion was not considered related. There were no significant differences in reported symptoms or routine monthly laboratory test results among the treatment groups. The authors concluded that rifapentine 900 mg given on a once weekly schedule was well-tolerated, and that a 1200 mg dose once weekly warranted further study.

The CDC-sponsored Tuberculosis Trials Consortium is currently conducting a prospective, randomized, phase III study of rifapentine for treatment of *latent* TB (“Study 26”, NCT 00023452 at http://www.clinicaltrials.gov/ct/show/NCT00023452?order=2). Subjects are randomized to receive either isoniazid daily for 9 months (standard therapy) or the combination of isoniazid plus rifapentine (900 mg) once weekly for 12 weeks. To date, over 2500 subjects have received the rifapentine regimen. While the details of tolerability are not yet available to the investigators, the data safety monitoring board has repeatedly stated that the tolerability of the rifapentine regimen is satisfactory.

Thus, rifapentine shows promise for treatment of TB. Specifically, rifamycin exposure greater than that achieved with the currently approved rifapentine dose of 600 mg twice weekly may

substantially increase a regimen's antituberculosis potency. Furthermore, development of acquired rifamycin resistance in some TB patients treated with once weekly rifapentine plus isoniazid indicates that isoniazid (with its shorter half-life of 1-4 hours) is not an optimal companion drug for rifapentine (Vernon et al., 1999; Weiner et al., 2003).

2.1.8 Safety and Tolerability of Rifapentine Administered Once-Daily or Thrice Weekly

The safety and tolerability of rifapentine used daily has been evaluated in a phase I pharmacokinetics study in healthy volunteers (Keung et al, 1999). Twenty-three healthy males were randomized to receive two of the following treatments in a two-period, four-treatment, incomplete block, cross-over design: single once-daily oral rifapentine doses of 150 mg, 300 mg, or 600 mg on study days 1 and 4 through 10, or single oral rifapentine 600 mg doses given every three days for four doses. All regimens reached steady state rifapentine concentrations, and rifapentine was well-tolerated in all four treatment periods. Urine discoloration was the most common adverse event, and occurred in all study subjects. Other treatment-related adverse events were upset stomach (n=4), lightheadedness (n=3), dry mouth (n=2), diarrhea (n=2), flatulence (n=1), and headache (n=1). No abnormalities of liver chemistries were reported.

The safety and tolerability of rifapentine used daily has also been evaluated by the manufacturer in clinical phase II studies in AIDS patients with bacteremia caused by *Mycobacterium avium* complex (MAC):

Multicenter Dose Escalation Study to Evaluate Tolerance, Safety, and Activity of Rifapentine Alone and in Combination Therapy in AIDS Patients with MAC Bacteremia (Hoechst Marion Roussel protocol number 000473PR**0005**).

The primary objective of this phase II, open-label, multicenter, dose-escalation study was to determine the tolerance and safety of escalating rifapentine doses alone and in combination therapy for disseminated MAC bacteremic patients with AIDS. This study started in 1995 and ended in 1996. Patients were treated with rifapentine monotherapy for 14 days, then randomized to combination therapy with clarithromycin or combination therapy with clarithromycin+ethambutol. The combination therapy phase lasted 28 days. Rifapentine was dosed once daily. Thirty patients were enrolled in the study; eight received rifapentine 450 mg/day, and 22 received rifapentine 300 mg/day. All patients experienced one or more nonserious adverse events; the high incidence was "indicative of the poor health status of this patient population". Seventeen (56.7%) patients had one or more treatment-related adverse events (adverse events defined by the investigator as "definitely", "probably", or "possibly" related to study medication. Among these 17 patients, 6 (20% of total) had a hematologic AE (2 patients with anemia, 3 with neutropenia, and 1 with hemoglobin/hematocrit disease), 6 patients had a hepatic/biliary AE (5 with bilirubinemia, 1 with cholestatic hepatitis), 4 patients (13%) had a body-as-a whole AE (3 with abdominal pain and 1 with ascites), and 3 (10%) had a gastrointestinal AE (2 with nausea and 1 with vomiting). There were 13 serious adverse events, seven of which were death, and six of which were nonfatal. None of the deaths were judged to be related to study medication. Among the 6 nonfatal SAEs, 1 was judged not related to study medication, one was judged unlikely to be related to study medication (blindness in a patient known to have CMV retinitis); two were judged possibly related to study medication (depression and suicide

attempt in one patient, and hepatic failure in one patient receiving 450 mg/day), and two were judged probably related to study medication (neutropenia in one patient receiving 300 mg/day), and dysarthria/confusion/AIDS in one patient). The rate of patient discontinuation due to treatment-related adverse events was higher among patients receiving 450 mg/day (4 patients among 8 who received this dose) than among patients receiving 300 mg/day (5 patients among 22 who received this dose). However, among the four patients receiving 450 mg/day who discontinued, in only one patient was the AE attributed to study medication, and this AE was listed as "dysarthria/confusion/AIDS".

Tolerance, Safety, and Activity of Rifapentine Alone and in Combination Therapy in AIDS Patients with Mycobacterium avium complex (MAC) Bacteremia. (Hoechst Marion Roussel protocol number 000473PR**0018**).
The primary objective of this phase II, open-label, multicenter trial was to determine the tolerability of rifapentine alone and in combination therapy in disseminated MAC bacteremic patients with AIDS. This study started in 1995 and ended in 1997; enrollment was closed in 1997 because of the marked decrease in the incidence of disseminated MAC infection following the widespread availability of potent combination antiretroviral therapy. Twenty-one patients were enrolled in this study. Four patients were enrolled to rifapentine monotherapy (3 received 300 mg/day and one received 450 mg/day); 3 patients completed 21 days of monotherapy and went on to complete 42 days of rifapentine-containing combination therapy. Seventeen patients were enrolled to rifapentine combination therapy only (11 received rifapentine 300 mg/day and 6 received rifapentine 450 mg/day); 13 completed 42 days of combination therapy. Twenty of 21 patients had ≥ one adverse event. As in the prior study, this was felt to be "indicative of the poor health status of this patient population." Seven patients (33.3%) had ≥ 1 treatment-related non-serious adverse event. Among these, 5 patients (23.8% of total patients) had a gastrointestinal adverse event, and three (14.3%) had a dermatologic adverse event. There were 17 serious adverse events (10 patients). Included among these serious adverse events were 3 deaths, none of which was judged by the investigator to be related to study medicines. There were 14 nonfatal serious adverse events, none of which was judged by the investigator to be related to study medicines. There were 4 permanent discontinuations due to adverse events (3 deaths, plus one additional discontinuation due to an AE not related to study medicines), and one temporary discontinuation (due to rash; study medicines were stopped, then reintroduced without return of the rash). Study conclusions were that adverse events were overwhelmingly symptoms associated with AIDS and few were attributable to study medicines, and that rifapentine was well tolerated at either dose level given as monotherapy or as combination therapy.

An Open-Label, Multicenter Extended Treatment Phase Study with Rifapentine in AIDS Patients Who Have Completed Protocol 000473PR0018 (Hoechst Marion Roussel protocol number 000473PR**0019**)
The primary objective of this study was to provide rifapentine for extended treatment to patients who, in the opinion of the investigator, responded to treatment during the preceding rifapentine protocol (000473PR0018). Eight patients were enrolled in this extended use study, and the duration of treatment for a given patient was based on individual response. Seven patients received rifapentine 300 mg/day, and one patient received rifapentine 450 mg/day. Median number of weeks of rifapentine use in this study was 18 (range 4 to 52

weeks, mean 24.5 weeks). Four nonserious adverse events were judged to be related (possibly, probably, or definitely) to study treatment – one patient each with dry mouth, conjunctival erythema, burning eyes, and erythematous ear canal. There were 14 serious adverse events including 3 deaths; none of the 14 SAEs was judged to be related to study treatment. Study conclusions were that adverse events were overwhelmingly symptoms associated with AIDS, and rifapentine was well-tolerated.

Importantly, individuals in the above studies had advanced AIDS complicated by disseminated MAC, each of which is associated with substantial morbidity and mortality. For example, Chaisson reported in 1992 that the median duration of survival after disseminated MAC diagnosis in AIDS patients was 221 days, and that the probability of one year survival from the time of disseminated MAC diagnosis was 0.29 (95% CI 0.24-0.34) (Chaisson 1992). More recently, Benson reported results of an AIDS Clinical Trials Group study of three contemporary, clarithromycin-based regimens for treatment of disseminated MAC in AIDS patients. Among the treatment groups, 8-25% died during the first four months of the study, and 28-50% died during the 48-week study period. Furthermore, 9-14% of patients stopped therapy due to protocol-defined toxicity, 9-25% had Grade 3 or higher anemia, and 6-14% had Grade 3 or higher hepatitis.

An additional phase I study conducted by S. Dorman and colleagues at Johns Hopkins evaluated the safety and tolerability of rifapentine 900 mg per dose administered thrice weekly in combination with moxifloxacin 400 mg once daily in healthy volunteers. Fifteen subjects were enrolled, including nine (60%) African-Americans, four (27%) Caucasians, one Hispanic, and one Asian; three (20%) were female and 12 (80%) were male. One subject withdrew for personal reasons after receiving two doses of moxifloxacin and prior to receiving rifapentine. Another withdrew after four doses of moxifloxacin alone plus one dose of moxifloxacin plus rifapentine, citing grade 1 side effects and job obligations. Thirteen subjects completed the study, three of whom were women. Mean age was 43.2 years (range 24-64 years). Mean mg/kg was 11.9 for rifapentine.There were no serious adverse events or grade 3 or 4 toxicities. Two subjects, both Caucasian women, experienced grade 2 adverse events after completion of study medications. One developed fever to 39.3^{o}C accompanied by nausea, anorexia, and hepatitis with ALT of 135 IU/L. The symptoms began on study day 20, 36 hrs after her final dose of study drugs; she had been asymptomatic with normal liver chemistries on day 18. Her symptoms resolved without specific intervention by day 22, and her ALT was normal on day 33. Evaluation for infectious etiologies was unrevealing. Her moxifloxacin AUC_{0-24} was 68.3 µg*h/mL on Day 4 and 55.3 µg*h/mL on Day 19, the highest among all participants. Her rifapentine AUC_{0-48} on Day 19 was 456.15 µg*h/mL (75th percentile), and her 25-desacetyl-rifapentine AUC_{0-48} was 364.5 µg*h/mL (50th percentile). The second subject developed fever to 38.6 ^{o}C with fatigue, malaise, and nausea on study day 21, two days after her last dose of study drugs. Her liver chemistries were normal, and laboratory testing failed to reveal an infectious source. She subsequently developed an urticarial rash and headache. Symptoms resolved without specific intervention by Day 35. Her rifapentine AUC_{0-48} after multiple doses was 223.3 µg*h/mL, and her 25-desacetyl-rifapentine AUC_{0-48} was 172.1 µg*h/mL, both significantly lower than the values for any other participant. Her Day 19 moxifloxacin AUC_{0-48} was 29.2 µg*h/mL (10th percentile).

2.1.9 Rifamycin Hypersensitivity Syndrome

Rifamycins have been associated with several types of immune-mediated allergic or hypersensitivity reactions, including a flu-like syndrome, acute renal failure, hemolytic anemia, thrombocytopenia, and anaphylactic events. The flu-like syndrome includes fever, chills, headache, arthralgias and or myalgias, typically arising within 1-4 hours after rifampin exposure. Available published literature indicates that it may be related to dose and frequency of administration. The table below shows information compiled by Grosset and Leventis from several international studies (Grosset 1983; Decroix 1971; Girling 1971; Eule 1974; Hong Kong Tuberculosis Treatment Services 1974a and 1974b).

	% of patients developing flu-like syndrome after treatment at indicated interval	
Rifampin dose (mg)	Twice-weekly	Once-weekly
600	4	10
900	8	22-31
1200-1800	16-22	35-57

Table 3. % of patients developing flu-like syndrome during rifampin-containing TB treatment

This syndrome has been associated with development of anti-rifampin antibodies or with complement activation (Martinez 1999). Typically, symptoms resolve within several hours. Symptoms can recur with rifamycin re-exposure, although in some instances symptoms can be controlled by switching to more frequent rifamycin dosing (Martinez 1999).

Immune-mediated acute renal failure, hemolytic anemia, thrombocytopenia, and anaphylactic reactions to rifampin are rare. These reactions can be associated with the flu-like syndrome or can occur in its absence, and they are almost exclusively seen in patients treated with intermittent rifamycin regimens (Martinez 1999). In most of the cases reported in the literature, the reactions have resolved within several days of discontinuing the rifamycin.

There are no published reports of flu-like syndrome occurring in the setting of rifapentine administration. In addition, there were no reports of flu-like syndrome in TBTC Study 22 or Study 25 (which assessed rifapentine 900 mg and rifapentine1200 mg given once-weekly in approximately 50 patients at each dose) (Benator 2002; Bock 2002). TBTC Study 26 is an open-label study of effectiveness and tolerability of once-weekly isoniazid and rifapentine 900 mg given for 3 months versus daily isoniazid for 9 months. As indicated above, to date, over 3500 subjects have received the rifapentine regimen, and while the details of tolerability are not yet available to Study 26 investigators, the data safety monitoring board has repeatedly stated that the tolerability of the isoniazid/rifapentine regimen is satisfactory.

As described above, in a phase I study conducted at Johns Hopkins of moxifloxacin co-administered with rifapentine 900 mg thrice weekly in healthy volunteers, 2 of 15 participants developed signs and symptoms compatible with rifamycin hypersensitivity and for which there was no other identifiable cause (S. Dorman, personal communication). One of these individuals had nausea, fever, elevated liver transaminases (maximum ALT of 135), and macular rash, all of which were of toxicity grade 2 or less, and all of which resolved within one week. The other

individual had fatigue, fever, and urticaria, all of which were of toxicity grade 2 or less, and all of which resolved completely within about 10 days; liver chemistries were normal during the event. **Both of these participants were female; among the 15 participants there was one additional female, and she tolerated the medications without problems.**

2.1.10 Preclinical Experience with Rifapentine and Moxifloxacin Combination Therapy for the Treatment of Tuberculosis

The similar half-lives of rifapentine (14-17 hours) and moxifloxacin (approximately 12 hours) make this pairing attractive, especially given the clinical observation that the pairing of isoniazid (with a shorter half-life, of 1-4 hours, depending on acetylator status) with rifapentine administered highly intermittently (ie once weekly) can lead to emergence of acquired rifamycin monoresistance in some patients.

In murine models, regimens that contain rifapentine plus moxifloxacin are strikingly effective for TB treatment. Since the clinical performance of once-weekly rifapentine (10 mg/kg/dose) paired with isoniazid has been slightly inferior to standard daily rifampin plus isoniazid regimens, Rosenthal and colleagues (2006) recently performed a series of studies in mice to determine the antimicrobial potency of intensive phase regimens in which rifapentine was used more frequently than once-weekly and in combination with moxifloxacin. They initially studied the combination of rifapentine (15 mg/kg/dose), moxifloxacin, and pyrazinamide ($P_{15}MZ$) administered twice weekly. They reproducibly showed that this regimen was significantly more bactericidal than daily RMZ, or, more importantly, than the standard regimen of daily RHZ (Figure 2). Furthermore, rifapentine (15 mg/kg) -containing regimens administered twice-weekly for 4 months had good sterilizing activity that was associated with very low relapse rates and was comparable to daily isoniazid plus rifampin-based regimens administered for 6 months.

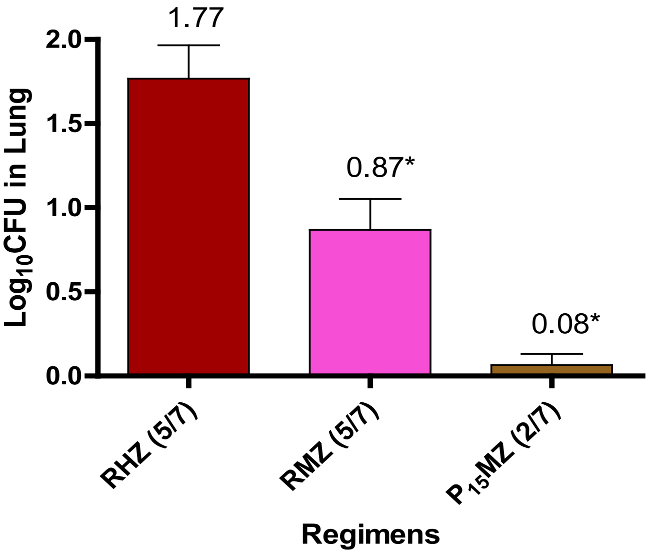

Figure 3. Decreased lung colony forming unit counts in mice treated for 2 months with twice-weekly rifapentine, moxifloxacin, and pyrazinamide ($P_{15}MZ$) compared to mice treated with daily rifampin, moxifloxacin, and pyrazinamide (RMZ) or daily rifampin, isoniazid and pyrazinamide (RHZ).

Subsequent experiments by Rosenthal and colleagues have evaluated the antimicrobial activity of MZ-containing regimens in which rifapentine was given at either higher dose or at more frequent intervals in mice. Overall, the antimicrobial activity increased with increasing dose or frequency of rifapentine. Administration of $P_{15}MZ$ thrice (3 times) weekly during the first eight

weeks of TB treatment was substantially more potent than $P_{15}MZ$ given twice weekly. Specifically, lung cfu counts at weeks of treatment were 4.5 log_{10} in the $P_{15}MZ$ twice weekly group versus 3.1 log_{10} in the $P_{15}MZ$ thrice weekly group. Lung cfu counts at 8 weeks were 0.16 log_{10} in the $P_{15}MZ$ twice weekly group, versus undetectable in the $P_{15}MZ$ thrice weekly group. Remarkably, the $P_{15}MZ$ thrice weekly regimen resulted in stable cure after 3 months of treatment, whereas the $P_{15}MZ$ twice weekly regimen required 4 months of treatment to achieve stable cure.

2.1.11 Clinical Experience with Rifapentine and Moxifloxacin Combination Therapy for the Treatment of Tuberculosis

There have been no human studies to date evaluating rifapentine-moxifloxacin combination regimens. As above, moxifloxacin (400 mg) given daily or thrice weekly in combination with rifampin, isoniazid, and pyrazinamide during the first 8 weeks of TB treatment was shown to be safe and well tolerated in TBTC Study 27 (Burman et al., 2006). Rifapentine at a dose of 600 mg (approximately 10 mg/kg) twice weekly was safe and well tolerated when administerd with daily isoniazid, pyrazinamide, and ethambutol for 8 weeks (Priftin package insert) or with isoniazid during the continuation phase of TB therapy (Benator el al. 2002; Tam et al. 1998). Higher doses of rifapentine (approximately 15 mg/kg) administered once weekly were well tolerated in combination with isoniazid during the continuation phase of TB treatment (Bock et al. 2002).

In our institution, we recently completed a multiple-dose, two-period, sequential-design pharmacokinetic study of 14 healthy volunteers in which subjects received moxifloxacin 400 mg daily for 4 days followed by moxifloxacin 400 mg daily coadministered with rifapentine 900 mg thrice-weekly. In this Phase I trial, the AUC_{0-24} for moxifloxacin decreased by 17% when moxifloxacin was coadministered with rifapentine, and the half-life ($t_{1/2}$) decreased from 11.1 to 8.9 hours, suggesting that rifapentine induces the metabolism of moxifloxacin. In studies with moxifloxacin and rifampin, a cousin drug to rifapentine, rifampin decreased moxifloxacin serum concentrations by 27-30% (Nijland et al. 2007; Weiner et al. 2007). In our Phase I study, we also found that rifapentine induced its own metabolism. Concentrations after multiple doses were 20% lower than after a single dose, and the halflife decreased from 18.5 to 14.8 hours. Since enzyme induction is unlikely to increase beyond a rifapentine dose of 900 mg (15 mg/kg) thrice weekly, the impact of daily rifapentine 7.5 mg/kg on the metabolism of moxifloxacin and rifapentine itself is anticipated to be similar

2.1.12 Rationale for Selected Rifapentine Dose/Schedule

Daily versus intermittent: Daily instead of twice- or thrice-weekly rifapentine is proposed for this study based on the marked antimycobacterial potency of daily rifapentine in the mouse model of TB treatment and the overall longer term objective of identifying TB treatment regimens shorter than the current six months duration. In the mouse model, intensive phase regimens containing rifapentine administered daily have substantially greater activity than intermittently administered regimens as assessed by cfu counts and relapse. Should daily rifapentine ultimately prove successful in shortening TB treatment duration, exploration of intermittent dosing would be warranted in the future for its potential feasibility in the setting of TB DOT programs. If daily rifapentine is unsafe or poorly tolerated, then future studies of intermittent dosing could be considered.

Rifapentine dose of 7.5 mg/kg/dose: Rifapentine daily dose of 7.5 mg/kg has greater activity than a 5 mg/kg daily dose as assessed by cfu counts and relapse in the mouse model.

Rifapentine administered 7 days/week instead of 5 days/week: In the DOT program setting, it is common for "daily" TB treatment to be administered 5 days per week on weekdays, with no treatment administered on weekends. We propose administration of TB treatment 7 days per week for two reasons. First, administration 7 days/week may minimize any risk of rifamycin hypersensitivity, should it occur with rifapentine. This is an important consideration, and we propose collection of clinical data and serum specimens in any participant with signs/symptoms potentially compatible with rifamycin hypersensitivity. Second, administration 7 days/week maximizes antimycobacterial potency.

Inclusion of moxifloxacin, isoniazid, and pyrazinamide in the experimental regimen: This strategy builds on the findings of Chaisson et al in the FDA-sponsored study in Brazil as described above. In addition, this approach is consistent with the overall goal of identifying a *regimen* that may ultimately allow treatment shortening.

2.1.13 Rationale for Population PK

For this study, the proposed rifapentine dose is 450 mg by mouth daily (7 days per week). This dose is not FDA-approved, and there is no published pharmacokinetic data using this dose. In the pharmacokinetic study described above (2.1.11), two female participants had grade 2 adverse events that had some similarities to rifamycin hypersensitivity syndrome. In addition, rifapentine induced its own metabolism and that of moxifloxacin such that concentrations of rifapentine and moxifloxacin after two weeks of co-administration were reduced by approximately 20 to 25% -- the clinical significance of this decrement is unclear. Finally, rifapentine pharmacokinetic/pharmacodynamic (PK/PD) parameters that correlate with TB treatment response and/or adverse events are unknown.

We therefore propose a population PK study in which all patients randomized to the experimental treatment arm (isoniazid+rifapentine+pyrazinamide+moxifloxacin) will undergo PK sampling at 5 time-points relative to one study dose. Concentrations of rifapentine, des-acetyl rifapentine, and moxifloxacin will be determined at each time point. Modeling will be used to derive and describe PK parameters including area under the concentration-time curve (AUC), maximal plasma concentration (C_{max}), half-life ($t_{1/2}$), and oral clearance (CL/F). For rifapentine and moxifloxacin, we will compare PK parameters including AUC, C_{max}, and $t_{1/2}$ between individuals experiencing AEs and those not experiencing AEs. Pharmacodynamic (PD) parameters including AUC/MIC and C_{max}/MIC between those with positive 2-month sputum culture results and those with negative 2-month sputum culture results.

The proposed PK study will provide new information about rifapentine PK parameters when the drug is used at a dose of 450 mg daily. Importantly, the proposed population PK design will also allow assessment of potential correlations between rifapentine and moxifloxacin PK parameters and safety, and between rifapentine and moxifloxacin PK/PD parameters and treatment response. This information is anticipated to be critical to understanding the role and dosing of rifapentine for TB treatment.

2.1.13 Study Drugs

RIFAPENTINE

Rifapentine is FDA-approved for the treatment of pulmonary TB in individuals receiving at least one other antituberculosis drug to which their *M. tuberculosis* isolate is susceptible. FDA approval was given in 1998 based on the results of “Clinical Study 008”, an open-label, prospective, randomized study of 722 patients with active pulmonary TB (Priftin package insert.

Rifapentine is a semisynthetic rifamycin derivative with a microbiologic profile similar to that of rifampin. Its structure differs from that of rifampin by the presence of a cyclopentyl ring instead of a methyl group at the piperazinyl moiety. It has a longer half-life than rifampin, and, like rifampin, rifapentine inhibits bacterial RNA synthesis by binding to the β-subunit of DNA-dependent RNA polymerase.

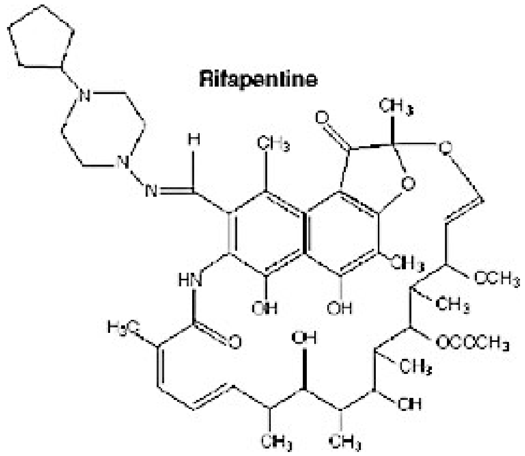

Pharmacokinetics

Rifapentine is well-absorbed from the gastrointestinal tract, with 70% bioavailability; when taken with food, its AUC and C_{max} increase by 43% and 44%, respectively (Priftin package insert). It reaches peak concentrations in the serum 5 to 6 hours after ingestion. Rifapentine and its 25-desacetyl metabolite are highly protein-bound, 97.7% and 93%, respectively, primarily to albumin. Rifapentine is metabolized by an esterase enzyme found in the liver and blood to 25-desacetylrifapentine, a microbiologically active metabolite which contributes about 40% of the drug’s overall activity. For *M. tuberculosis*, the MIC of 25-desacetyl rifapentine is 0.25 mcg/mL, while that of rifapentine is 0.05 mcg/ml. The drug and the active metabolite have half-lives of 14-17 and 13 hours, respectively. The drug is excreted in bile and eliminated in feces. Less than 10% of rifapentine is excreted in the urine as unchanged drug. Single dose rifapentine pharmacokinetics have been shown to be similar in healthy females and healthy males (Keung et al., 1998). Table 4 shows measured PK parameters in healthy volunteers at doses of 900 mg po thrice weekly (S. Dorman et al. unpublished) and doses of 600 mg po once daily and 300 mg po once daily (Keung et al. 1999).

	AUC (ug*h/mL)	Cmax (ug/mL)
Rifapentine 900 mg po thrice weekly	410	21.2
Rifapentine 600 mg po once daily	367	24.3
Rifapentine 300 mg po once daily	159	11.2

Table 4. AUC and Cmax for rifapentine administered to healthy volunteers

Drug-Drug Interactions
Rifapentine, like other rifamycins, induces CYP3A4, 2C8, and 2C9, which can lead to more rapid metabolism and clearance of many drugs. Rifapentine is a less potent inducer of CYP3A than is rifampin (Burman et al., 2001). Rifamycins are also known to induce the activity of phase II enzymes such as glucuronosyltransferase and sulphotransferase and may reduce levels of drugs metabolized by those pathways. Due to drug-drug interactions, rifampin is contraindicated for use with HIV protease inhibitors (with the exception of ritonavir), delavirdine, cyclosporine, tacrolimus, itraconazole, and ketokonazole. When administered concomitantly with rifamycins, many other drugs including warfarin require dose adjustment.

Dosage
For treatment of pulmonary TB, the FDA-approved dose of rifapentine is 600 mg twice weekly for two months (intensive phase of TB treatment), with an interval of no less than 3 days (72 hours) between doses, as part of a regimen that includes appropriate daily companion antituberculosis drugs that may include isoniazid, pyrazinamide, and ethambutol or streptomycin. According to the package insert, following completion of intensive phase, continuation phase treatment should be given for 4 months using rifapentine 600 mg administered once weekly in combination with appropriate companion antituberculosis drugs to which the isolate is susceptible.

However, as detailed above, preclinical and clinical studies indicate that maximal antituberculosis activity is achieved at doses higher than 600 mg and intervals shorter than twice weekly. In addition, a phase II clinical trial established the safety and tolerability of rifapentine doses as high as 1200 mg administered once-weekly (Bock et al, 2002).

Rifapentine is available as 150 mg tablets.

Tolerability
Rifapentine, like other rifamycins, causes red-orange discoloration of body fluids and can stain contact lenses. In clinical trials in which rifapentine was combined with isoniazid and other antituberculosis drugs, rates of adverse reactions were similar with rifampin and rifapentine, with increased liver aminotransferase activity in about 5% of patients. The only adverse effect that has occurred more often with rifapentine than with rifampin has been hyperuricemia when the drug was given twice- weekly; of note, hyperuricemia was attributed to pyrazinamide that was administered concommitantly. Other adverse reactions which occurred in 1-10% of patients included the following: hypertension, dizziness, headaches, rash, gastrointestinal upset,

pyuriaor proteinuria, cytopenias, arthralgias, and hemoptysis (Priftin package Insert). Rifapentine may be associated with a hypersensitivity syndrome – this is described in section 2.1.9.

MOXIFLOXACIN

Moxifloxacin is a fluoroquinolone antibacterial, which is distinguished by a methoxy group at the C-8 position and an S,S-configured diazabicyclononyl ring moiety at the C-7 position. It works by inhibiting topoisomerase II (DNA gyrase) and topoisomerase IV which are required for bacterial DNA replication, transcription, repair, and recombination.

Moxifloxacin is FDA-approved for the treatment of adults (≥ 18 years of age) with infections caused by susceptible strains of the designated microorganisms in the following conditions: acute bacterial sinusitis (*Streptococcus pneumoniae, Haemophilus influenzae, Moraxella catarrhalis*), acute bacterial exacerbation of chronic bronchitis (*Streptococcus pneumoniae, Haemophilus influenzae, Haemophilus parainfluenzae, Klebsiella pneumoniae, Staphylococcus aureus, Moraxella catarrhalis*), and community acquired pneumonia of mild to moderate severity (*Streptococcus pneumoniae, Haemophilus influenzae, Mycoplasma pneumoniae, Chlamydia pneumoniae, Moraxella catarrhalis*).

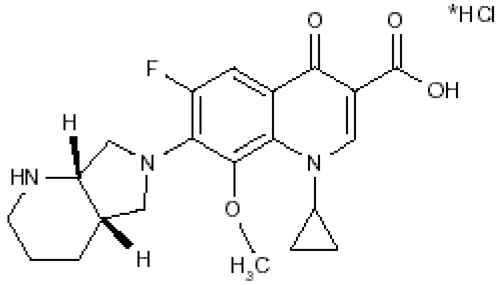

Moxifloxacin structure [Diagram obtained from package insert]

Pharmacokinetics
Moxifloxacin has 90% bioavailability, and high fat meals do not change its absorption (Ballow et al., 1999; Lettieri et al., 2001). Its pharmacokinetics are linear in the range of 50-800mg with single doses and up to 600mg with daily dosing over 10 days.(Stass et al. 2001a) It is 50% bound to serum proteins and is widely distributed with tissue concentrations often higher than plasma concentrations (Avelox package insert). Fifty-two percent of a dose is metabolized via glucuronide and sulfate conjugation. The cytochrome p450 system is not involved in moxifloxacin metabolism, and moxifloxacin does not affect the cytochrome p450 enzyme system. The sulfate conjugate, M1, accounts for 38% of a dose and is eliminated in the feces (Stass et al., 1999). The glucuronide conjugate, M2, accounts for 14% of a dose and is excreted in urine. Approximately 45% of a dose is excreted unchanged. The metabolites M1 and M2 are not active. Maximum concentration is achieved at approximately 1-3 hours after a dose, and the mean half-life is approximately 12 hours.

Drug-drug interactions

Interaction studies have been performed with moxifloxacin and theophylline, warfarin, digoxin, probenecid, ranitidine, glyburide, iron, calcium supplements, sucralfate, and antacids (Stass et al. 2001 b-h). Only a few clinically significant interactions have been described. Divalent or trivalent cations (with the exception of calcium), iron, antacids, and sucralfate can lower the bioavailability of moxifloxacin and should not be given concurrently. In addition, moxifloxacin should not be given with class IA or III antiarrhythmics, as quinolones have been shown to prolong the QTc interval in dog models.

Dose
The FDA-approved oral dose of moxifloxacin is one 400 mg tablet taken by mouth once every 24 hours. The durations of therapy recommended in the package insert are 10 days for acute bacterial sinusitis, 5 days for acute bacterial exacerbation of chronic bronchitis, and 10 days for community acquired pneumonia.

Tolerability
In the usual dose of 400mg once daily, moxifloxacin is safe and is associated with relatively few serious adverse reactions (Avelox package insert). Adverse reactions that occur in greater than 2% of patients include nausea, diarrhea, and dizziness. In >0.1 and <2% of patients, QT prolongation, insomnia, tremor, anxiety, peripheral neuropathy, increased hepatic enzymes, cytopenias, arthralgias, tendon rupture, ventricular tachycardia, cholestasis, and seizures can occur. Although fluoroquinolones are usually used short-term for acute conditions, they have been found to be safe and effective in long-term use for chronic infections such as osteomyelitis, prostatitis, chronic urinary tract infection, and skin and skin structure infections (Ball 1989; Segev et al. 1999). A six-month treatment regimen for TB that included moxifloxacin was well-tolerated (Valerio et al., 2003). In a recent report from Italy, 38 patients with drug-resistant TB or comorbidities interfering with standard chemotherapy were treated with combination therapy including daily moxifloxacin at a dose of 400mg once daily for six months in combination with other anti-TB drugs -- no serious side effects were observed, and all patients completed the prescribed course of therapy (Codecasa et al., 2006).

Cardiotoxicity
The QT interval is the electrocardiographic measurement that describes the period between onset of ventricular depolarization and the end of the repolarization process. Prolongation of this interval is thought to be associated with an increased risk of ventricular tachyarrhythmias. Moxifloxacin causes a mild prolongation of the corrected QT interval (Demolis et al. 2000; Ball P 2000)) The mean effect on QT interval in 787 patients in Phase III clinical trials was 6 $\pm$ 26 milliseconds, but no morbidity or mortality was attributable to QT prolongation (Ball et al., 2004). Among over 7000 patients who received oral moxifloxacin 400 mg/d in Phase II/III clinical trials, no case of ventricular arrhythmia was reported (Iannini et al., 2002). Two large postmarketing observational studies have been completed, which included almost 35,000 patients treated with moxifloxacin in routine clinical practice. Those postmarketing studies examined all known quinolone class effects, and reported no unusual adverse reactions (Iannini et al., 2001). In over 2.2 million patients treated with moxifloxacin there has been no evidence for an increased incidence of ventricular arrhythmia when compared to the overall population (Iannini et al., 2001). For these reasons, electrocardiographic monitoring is no longer recommended for clinical trials involving moxifloxacin. Pharmacokinetic studies between moxifloxacin and other

drugs that prolong the QT interval such as cisapride, erythromycin, antipsychotics and tricyclic antidepressants have not been performed. An additive effect of moxifloxacin and these drugs cannot be excluded.

Phototoxicity/Photosensitivity

Photosensitivity is defined as a non-immunological, light-activated irritation that occurs following exposure to a photoactive chemical. This is an infrequent AE of most fluoroquinolones. Moxifloxacin has been found to have no negative phototoxic effects and in some patients was shown to be photoprotective at specific wavelengths.

Tendinopathy/Tendonitis

The estimated incidence of fluoroquinolone-induced tendonopathy is 15 to 20 per 100,000 (Royer et al. 1994). Fluoroquinolone-induced tendonopathy is characterized by sudden onset of swelling and tenderness concurrent with or shortly after discontinuation of fluoroquinolone therapy, and is accompanied by tendon rupture in about 33% of all cases (Zabraniecki et al. 1996) The main site affected is the Achilles tendon, though tendonitis has been reported to involve the shoulder, knee, hand, and plantar aponeuroeses. (Hayem et al. 1995) Rarely, Achilles tendon rupture has been noted months after fluoroquinolone discontinuation (Pierfieet et al. 1996). Concomitant use of corticosteroids is considered to be a risk factor for developing tendinopathy while taking fluoroquinolones. (Harrell 1999) Treatment involves discontinuation of the fluoroquinolone and resting the tendon. In over 10 million patients treated with moxifloxacin only 3 patients with tendon rupture have been reported -- all had received concomitant corticosteroid treatment.

Retinal toxicity

Ocular toxicity was not observed in 6-month moxifloxacin repeat dose studies in rats and monkeys. In beagles, electroretinographic changes were observed in a 2-week study at doses of 60 and 90 mg/kg. Histopathological changes were observed in the retina from 1 of 4 dogs at 90 mg/kg, a dose associated with mortality in this study (von Keutz et al. 1999). In summary, ocular toxicity in animals has only been observed in one species (dogs) at a dose that was associated with severe systemic toxicity. Ocular toxicity has not been reported from clinical trials or post-marketing surveys involving moxifloxacin.

Monitoring for retinal toxicity will be performed among all patients in this clinical trial because of the inclusion of ethambutol, a drug with known retinal toxicity (see below). The visual testing done to detect ethambutol retinal toxicity should be adequate to screen for possible retinal toxicity from moxifloxacin.

Glucose Homeostasis

Recently, clinical data has emerged indicating that the fluoroquinolone gatifloxacin can affect glucose homeostasis, resulting in uncommon but clinically significant hypo- or hyperglycemic events. In a population-based case-control study in which case patients (n=788) were individuals treated in the hospital for hypoglycemia after outpatient antibiotic therapy, compared with macrolide antibiotics, gatifloxacin was associated with an increased risk of hypoglycemia (adjusted odds ratio [AOR] 4.3, 95% CI 2.9-6.3), levofloxacin was associated with slightly increased risk (AOR 1.5), but no risk was seen with moxifloxacin (Park-Wyllie et al., 2006). In a separate analysis, pooled data from all completed moxifloxacin phase II/III trials and post-

marketing studies has been analyzed (Gavin III et al., 2004). The phase II/III database was comprised of 14,731 patients (8474 who received moxifloxacin, and 6257 who received a comparator antimicrobial). There was no drug-related hypoglycemic adverse events reported for moxifloxacin, but 3 drug-related adverse events were reported for other comparator fluoroquinolones (2 for levofloxacin and one for trovafloxacin). Data from five moxifloxacin post-marketing studies (46,130 persons) yielded no episodes of hypoglycemia.

ETHAMBUTOL

Ethambutol is an ethylene derivative of butane that interferes with cell wall synthesis in mycobacteria; other bacteria are uniformly resistant to ethambutol. In the treatment of human TB, ethambutol is effective in preventing the emergence of drug resistant strains, although it has no sterilizing activity at clinically-tolerated doses (Kohno et al. 1992).

Pharmacokinetics
Ethambutol is well absorbed from the gastrointestinal tract, reaching peak serum concentrations of 3-5 mcg/ml in normal volunteers 2-4 hours after a dose. Food slows absorption and decreases the peak serum concentration by 10-20%, but has no effect on the total systemic exposure (AUC). Antacids decrease both the peak serum concentration and AUC, and so should not be administered at the same time. Ethambutol is primarily eliminated by the kidneys as unchanged drug; the serum half-life averages 4 hours. Patients with renal insufficiency are prone to accumulation of the drug and the resultant toxicity.

Drug-drug interactions
There are no known drug–drug interactions involving ethambutol.

Toxicity
Ethambutol is usually well-tolerated with low rates of skin rash, nausea, vomiting, or diarrhea. Fever, allergic reactions, abdominal pain, mental status changes, peripheral neuropathy, and increased liver function tests have rarely been associated with ethambutol. Adverse events occur in less than 2% of patients receiving ethambutol at the 15 mg/kg dose and include decreased visual acuity (0.8%), rash (0.5%) and asymptomatic hyperuricemia (Patel et al. 1995). The most common serious side effect of ethambutol is retinal toxicity, often first perceived as a decrease in color perception. Patients receiving ethambutol should be instructed about symptoms of ocular toxicity. If stopped promptly, permanent visual loss is rare among patients with ethambutol-related retinal toxicity. Rates of retinal toxicity are very low when the drug is given for relatively short periods of time, as is the case in this study.

ISONIAZID

Isoniazid is the hydrazide of isonicotinic acid and is one of the primary drugs for TB treatment. The activity of isoniazid is limited to the mycobacteria of the *M tuberculosis* complex; it is bactericidal for rapidly dividing organisms and bacteriostatic for “resting” bacilli. The probable mechanism of action is the inhibition of the biosynthesis of mycolic acids, a component of the mycobacterial cell wall.

Pharmacokinetics

Isoniazid is generally well absorbed; food and antacids decrease the rate, but not the extent of absorption. The peak blood levels of isoniazid, 3 to 5 mcg/ml, are obtained 30 minutes to 2 hours after ingestion of routine doses (Peloquin et al. 1999). It diffuses into all body fluids and cells and penetrates into the caseous material of a tuberculoma or pulmonary cavity. In the liver, it is acetylated to inactive metabolites, and 75% to 95% of the dose is excreted as inactive metabolites in the urine within 24 hours. Isoniazid clearance rates depend on 2 metabolic phenotypes, slow and fast acetylation, which are associated with race, but not gender (Ellard. 1984). The isoniazid AUC among persons who have fast acetylation is 30% to 50% of that among persons who have slow acetylation. Because isoniazid is well tolerated over a wide range of therapeutic doses, a single dose per body mass is recommended. Persons who have rapid acetylation achieve effective concentrations, while persons who have slow acetylation do not experience increased toxicity. Half-life ($t_{1/2}$) may vary from 1 hour in fast acetylators ($t_{1/2}$ < 90min) to 3 hours in slow acetylators ($t_{1/2}$ >90min).

Drug-drug interactions
Isoniazid decreases the clearance of some medications that are metabolized in the liver (particularly carbamazepine, phenytoin, diazepam) (Baciewicz et al. 1985). However in the context of multidrug therapy including rifampin, these potential drug-drug interaction are of little significance because the effect of isoniazid is counteracted by the more potent opposing effect of rifampin (Kay et al. 1985).

Toxicity
The total incidence of all adverse effects from isoniazid is approximately 5%, many of which do not require discontinuation of the drug. Peripheral neurotoxicity is dose dependent and it is uncommon (<0.2%) at conventional doses. The risk of peripheral neuritis increases for persons who are malnourished or predisposed to neuritis by other illnesses. Concomitant administration of pyridoxine (vitamin B_6) is recommended for these persons, and will be given to all patients receiving concomitant isoniazid in this trial. Other nervous system reactions are rare at normal doses, and they include convulsions, encephalopathy, optic neuritis, memory impairment, and psychosis. Gastrointestinal adverse effects include nausea, vomiting, and epigastric distress. Asymptomatic elevation of aminotransferases is common and occurs in 10-20% of persons receiving isoniazid. However, idiosyncratic severe hepatic reactions are uncommon but are more likely in older persons (up to 2.3% hepatitis incidence in persons more than 50 years old), and may be life threatening. Daily consumption of alcohol increases the risk of isoniazid-associated hepatotoxicity by approximately 4-fold. The risk of isoniazid-induced hepatotoxicity may also be increased in the postpartum period. The prodromal symptoms of hepatotoxicity are anorexia, nausea, vomiting, fatigue, malaise, and weakness; persons who take isoniazid and have these symptoms should stop therapy and be evaluated immediately.

RIFAMPIN

Rifampin is a semi-synthetic rifamycin derivative that is highly active against mycobacteria, most gram-positive bacteria, and some gram-negative bacteria. It is bactericidal for both intracellular and extracellular microorganisms. By inhibiting prokaryotic DNA-dependent RNA polymerase, it suppresses the early elongation of the nucleotide chain in RNA synthesis.

Pharmacokinetics
Rifampin is normally absorbed completely when taken orally, but food delays absorption. After 1.5 to 2 hours, a 600 mg dose yields a peak blood level of 8-20 mcg/ml. The half-life of rifampin varies from 2 to 5 hours, and it is shortened by approximately 20-40% after the first week of daily treatment because of the induction of hepatic microsomal enzymes. The half-life is unaffected by renal impairment but is increased by liver disease or biliary obstruction. Rifampin is deacetylated to an enterohepatically-recirculated active metabolite, and 50% to 60% is excreted in the feces. Up to 30% of a dose is excreted in the urine. Approximately 85% of circulating rifampin is bound to plasma proteins, and is widely distributed throughout the body.

Drug-drug interactions
Rifampin is a potent inducer of a number of hepatic enzymes involved in the metabolism of drugs and some hormones (Venkatesan 1992). This enzyme induction causes more rapid elimination (and potential loss of efficacy) of many drugs. For some medications, this loss in pharmacological activity effect is dramatic, and rifampin cannot be used if one of these medications is thought to be essential. Medications for which concomitant rifampin is contraindicated include: HIV-1 protease inhibitors (other than ritonavir), delavirdine, cyclosporine, tacrolimus, itraconazole, and ketoconazole. For many other medications, the dose can be increased to compensate for the effect of rifampin.

Toxicity
In the usual daily doses of 10 mg/kg (maximum 600 mg), rifampin is well tolerated. It often causes harmless but disconcerting red-orange discoloration of tears, sweat, saliva, feces, and urine. Less than 4% of TB patients experience significant adverse reactions to rifampin. Gastrointestinal adverse effects are the most common, and they include epigastric distress, anorexia, nausea, vomiting, cramps, and diarrhea. Hepatitis rarely occurs in persons who have normal baseline hepatic function. The incidence of hepatitis may be increased for older persons and those who have chronic liver disease or alcoholism, but remains substantially lower than that for pyrazinamide or isoniazid. Rifampin can cause a flu-like syndrome of fever, chills, and myalgia, although this is uncommon using the 600 mg dose given daily or thrice-weekly. In a very small proportion of patients the flu-like syndrome is associated with interstitial nephritis, acute tubular necrosis, thrombocytopenia, hemolytic anemia, and shock; this is described in detail in Section 2.1.9. There may be changes in menstruation.

<u>PYRAZINAMIDE</u>

Pyrazinamide is an analog of nicotinamide and has unique activity against *M tuberculosis*, allowing the duration of treatment to be decreased from 9 months to 6 months (assuming rifampin is used throughout). The mechanism of action of pyrazinamide remains unknown.

Pharmacokinetics
Pyrazinamide is well-absorbed from the gastrointestinal tract and widely distributed into all tissues, including the cerebrospinal fluid (Ellard et al. 1987). Usual doses are 15-30 mg/kg/d, up to 2 gm/d. Peak serum concentrations of about 45 mcg/ml are achieved approximately 2-3 hours after a dose. Food and antacids do not significantly affect the absorption of pyrazinamide. The half-life of pyrazinamide is approximately 9-10 hours Acocella et al. 1985), and is prolonged in the presence of hepatic insufficiency (Lacroix et al. 1990). Pyrazinamide is metabolized to

pyrazinoic acid by the hepatic microsomal enzyme pyrazinamide deamidase. Approximately 40% of a dose is recovered in the urine as pyrazinoic acid and an additional 4% is excreted in the urine as the unchanged parent drug (Ellard. 1969). The remaining drug is thought to be excreted in the bile.

Drug-drug interactions
There are no known clinically significant drug-drug interactions involving pyrazinamide.

Toxicity
The most frequent side effects are skin rash, gastrointestinal intolerance, hepatotoxicity (1.3%), arthralgias (1-7%), hyperuricemia due to blockade of urate excretion (up to 66%), and rarely acute gouty arthritis (Patel et al. 1995; Ormerod et al. 1996). These side effects are seldom dose-limiting. Asymptomatic elevations in serum uric acid are frequent, usually occur during the first or second month of treatment, and are self-limited and require no specific treatment (Zierski et al. 1980). Minor arthralgias also may occur during pyrazinamide treatment and can usually be treated with salicylates or non-steroidal inflammatory agents such as indomethacin while continuing the drug. The most common serious side effect of pyrazinamide is hepatotoxicity. In 2 randomized clinical trials the addition of pyrazinamide to RH did not increase the rates of hepatotoxicity above that seen with the latter 2 drugs alone (Zierski et al. 1980; Combs et al. 1990). However, 3 recent retrospective cohort studies suggest that the incidence of pyrazinamide-induced hepatitis during active TB treatment is higher than that for other first-line TB drugs, and higher than previously recognized (Yee et al. 2003; Schaberg et al. 1996; Dossing et al. 1996). Results from these latter studies underscore the importance of identification of new, less toxic yet effective, TB treatment regimens.

2.2 Rationale

Study design and hypothesis
This will be a two-center, randomized, open-label trial of 2 oral antimicrobial regimens for the first 8 weeks (intensive phase) treatment of sputum AFB smear-positive pulmonary TB. The experimental regimen will substitute rifapentine for rifampin, and moxifloxacin for ethambutol. Our hypothesis is that, for the treatment of smear positive, culture-confirmed pulmonary TB, the experimental regimen will have more potent antimycobacterial activity than, and have comparable rates of toxicity as, the standard control regimen.

Intervention
Experimental Arm: Isoniazid plus Rifapentine 300mg/450mg based on weight plus pyrazinamide plus Moxifloxacin 400mg administered 7 days per week for 8 weeks, followed by a standard continuation phase TB treatment regimen.

Control Arm: Isoniazid plus rifampin plus pyrazinamide plus ethambutol, administered 7 days per week for 8 weeks, followed by a standard continuation phase TB treatment regimen.

Doses of study medications during intensive phase are listed in Table 4. All medications will be administered orally and in standard doses except for rifapentine which will be administered at a shorter dosing interval (7 instead of 2 days per week). The rationale for the shorter interval of

rifapentine administration is superior anti-mycobacterial activity (compared with twice-weekly), and safety in a Phase I study (Keung et al. 1998).

Population
Subjects will be adults (HIV positive with CD4 ≥ 350 cells/cu mm, or HIV negative) with respiratory smear positive, culture-confirmed pulmonary TB at Hospital Universitario Clementino Fraga Filho in Rio de Janeiro, Brazil, the Núcleo de Doenças Infecciosas of the Federal University of Espitio Santo, Brazil and the Johns Hopkins Medical Institution, Baltimore, Maryland. Given the magnitude of TB co-infection in patients with HIV, it is important to evaluate new treatment regimens in the HIV-infected population.

Significance
New drugs are urgently needed to shorten the duration of TB treatment and to facilitate the delivery of DOT. This study will evaluate the potency and safety of a novel intensive phase regimen. Superior bacteriologic outcomes (with similar safety outcomes) in the experimental arm compared to the control arm would provide strong evidence that a short (e.g. 4 months), effective regimen for pulmonary TB may be feasible using a moxifloxacin plus rifapentine-based regimen.

Prompt evaluation of the proposed experimental regimen is warranted for several reasons. Rosenthal's recent findings (2006) of the enhanced potency of rifapentine-based regimens have important implications for simplification and shortening of standard TB therapy. In addition, results from the mouse model reproducibly indicate that moxifloxacin plus rifapentine intensive phase treatment regimens have exceptional sterilizing activity and are associated with very low relapse rates after 4 months of therapy (Nuermberger et al. 2004a; Nuermberger et al. 2004b). These results are important since the mouse model of TB treatment has been shown to recapitulate human TB treatment with regard to treatment-shortening effects of rifampin and pyrazinamide.

2.3 Potential Risks and Benefits

2.3.1 Potential Risks

The potential risks of study participation are medical and confidentiality risks. The principal medical risks are those of hepatoxicity, cardiotoxicity, and TB disease treatment failure or relapse.

<u>HEPATOTOXICITY</u>

Isoniazid, rifampin, rifapentine, and pyrazinamide each are associated with asymptomatic elevations of aminotransferases (relatively common), and hepatitis (rare). When used in combination during standard TB treatment, there is approximately a 3% risk of hepatitis. During this study, hepatotoxicity risk will be minimized by excluding persons with laboratory evidence of pre-existing significant hepatic dysfunction, and by frequent monitoring of liver chemistries during intensive phase treatment.

CARDIOTOXICITY

As detailed above, moxifloxacin causes a mild prolongation of the QT interval, but there has been no evidence for an increased incidence of ventricular arrhythmia, sudden death, or syncope in patients treated with moxifloxacin compared to the overall population. For the latter reason, electrocardiographic monitoring is no longer recommended for clinical trials involving moxifloxacin.

In this study, moxifloxacin will not be used in patients receiving either class Ia or class III antiarrhythmic agents, due to lack of clinical experience with moxifloxacin in these patient populations. Pharmacokinetic studies between moxifloxacin and other drugs that prolong the QT interval have not been performed, and an additive effect of moxifloxacin and these drugs cannot be excluded. Therefore, moxifloxacin will not used in patients receiving terfenadine, cisapride, erythromycin, clarithromycin, phenothiazines, haloperidol, olanzapine, ziprasidone, fluconazole, or tricyclic antidepressants (see Exclusion criteria). In this study, monitoring for cardiotoxicity will be done by assessment, at study visits, for palpitations, dizziness, or syncope.

TB DISEASE TREATMENT FAILURE

TB treatment failure is defined as a positive culture for *M. tuberculosis* after completion of 4 months of treatment. Study subjects in both treatment arms will have frequent assessments for treatment failure while on treatment.

OTHER MEDICAL RISKS

Each of the study medications has potential additional side effects, but these are uncommon and/or mild. Rifapentine can cause red-orange discoloration of body fluids. Moxifloxacin can cause gastrointestinal effects (including nausea, diarrhea, dizziness, abdominal pain, vomiting, dyspepsia, and taste perversion), skin rash, phototoxicity/photosensitivity, tendonopathy/ tendonitis, seizures, and retinal toxicity; these data are from clinical efficacy trials in over 8600 patients who received moxifloxacin 400 mg daily (intravenous or oral) for up to 2 weeks (Avelox package insert, 2005). Ethambutol can cause skin rash, nausea, vomiting, diarrhea, fever, allergic reactions, abdominal pain, mental status changes, peripheral neuropathy, and retinal toxicity. Isoniazid can cause gastrointestinal effects (including nausea, vomiting, epigastric discomfort), peripheral neuropathy, seizures, encephalopathy, optic neuritis, memory impairment, and psychosis. Rifampin can cause gastrointestinal effects (including nausea, vomiting, epigastric discomfort, anorexia, cramps, and diarrhea), flu-like syndrome (including myalgias, fatigue, interstitial nephritis, acute tubular necrosis, thrombocytopenia, hemolytic anemia, and shock), and red-orange discoloration of body fluids. Pyrazinamide can cause skin rash, pruritis, gastrointestinal effects (nausea, vomiting, anorexia), arthralgias, hyperuricemia, and acute gouty arthritis.

Other medical risks are small, and include those associated with venipuncture, chest X-ray, and sputum induction. The risks of drawing blood from a vein include discomfort at the site of puncture; possible bruising and swelling around the puncture site; rarely an infection; and, uncommonly, faintness from the procedure. The risks of chest X-ray include a slight risk of damage to cells, tissue, or a fetus from being exposed to radiation; these risks will be minimized

by use of established procedures and a certified technologist. Risks to a fetus will be minimized by not enrolling pregnant women into the study; women of child-bearing potential who are being screened for the study will be screened for pregnancy prior to performing a chest X-ray. The risks of sputum induction include wheezing and shortness of breath, but these are very uncommon.

CONFIDENTIALITY

Risks to confidentiality will be minimized as detailed in the sections, "Ethics/Protection of Human Subjects" and "Data Handling and Record Keeping".

2.3.2 Known Potential Benefits

Participation in this study may lead to a better understanding of the antimicrobial potency and safety of the study regimen, and may improve understanding of TB treatment overall, thereby potentially helping other TB patients in the future.

Participants in this study will be followed closely by a team of trained personnel who will provide directly observed TB treatment (DOT), and close monitoring for drug toxicity. While DOT is recommended by the WHO, and is the standard of care in the United States, it is not yet available to all TB patients in Rio de Janeiro. Therefore study participants in Brazil will be provided with a level of care that exceeds that which may be otherwise available to them.

3 OBJECTIVES

3.1 Study Objectives

HYPOTHESIS:
The hypothesis to be tested is that the experimental regimen will have more potent antimycobacterial activity than the standard regimen when administered during the first 8 weeks of therapy (intensive phase) for smear positive, culture-confirmed pulmonary TB. In addition, we hypothesize that the experimental and control regimens will have comparable rates of toxicity.

PRIMARY OBJECTIVES:

To compare, by treatment group, the proportions of patients with negative sputum cultures at the end of intensive phase therapy.

To compare the safety and tolerability of the 2 intensive phase regimens.

SECONDARY OBJECTIVES:

To compare the time to respiratory culture conversion of the 2 intensive phase regimens, using data from weekly cultures.

To compare, by treatment group, the proportions of subjects who experience treatment failure.

To compare, by HIV serostatus, a) the safety of the 2 intensive phase regimens, b) the proportions of patients with negative sputum cultures at the end of intensive phase therapy, and c) the time to culture conversion using data from weekly cultures.

To compare, in subjects with versus without cavitation on baseline chest x-ray, the proportions of patients with negative sputum cultures at the end of intensive phase therapy.

To store serum for future assessment of hypersensitivity to study drugs, should it occur.

To identify, for rifapentine and moxifloxacin, the PK/PD parameters that correlate most strongly with TB treatment response as measured by 2-month culture conversion;

To identify, for rifapentine and moxifloxacin, the PK/PD parameters that correlate most strongly with adverse events;

To describe the pharmacokinetics of rifapentine administered at 450 mg daily in the context of isoniazid, pyrazinamide, and moxifloxacin when used to treat pulmonary TB.

3.2 Study Outcome Measures

3.2.1 Primary Outcome Measures

Primary Endpoint – proportion of patients in each arm with negative sputum cultures, by Lowenstein Jensen culture, at the time of completion of 8 weeks of intensive phase therapy. The end of the intensive phase of therapy will be defined by completion of the required number of directly-observed doses. In order to maximize the likelihood that each subject can be

evaluated for the primary endpoint, 2 sputum cultures will be obtained at the end of intensive phase. These 2 cultures must have been obtained (a) 0 to 7 days after the completion of the intensive phase of therapy, and (b) prior to receiving more than 1 dose of continuation phase therapy. Sputum cultures that are overgrown by bacteria and/or yeast will be considered unevaluable. Sputum culture conversion will be based on the results of both sputum cultures performed at the end of the intensive phase of therapy. In order to conclude that sputum culture conversion was achieved, neither culture should be positive for *M. tuberculosis*, and at least one culture should be evaluable. Subjects who are unable to produce a specimen for sputum culture despite an attempt with sputum induction will be considered to have had a negative culture on that date.

Safety and tolerability. The main outcome will be the proportion of patients who discontinue the assigned study treatment regimen for any reason. Other aspects of safety and tolerability that will be assessed as secondary endpoints include proportions of patients who discontinue the assigned treatment regimen due to toxicity related to study drug(s), all-cause mortality, mortality related to the study drug(s), the occurrence of Grade 3 and 4 toxicities, and the rate and types of toxicity thought related to study drug by the investigator.

3.2.2 Secondary Outcome Measures

Time to sputum culture conversion. The date of sputum culture conversion will be defined as the day on which sputum for the first negative culture was obtained, with all subsequent sputum cultures negative for *M. tuberculosis*.

A population PK model. See Section 11.4 for model details and PK analysis.

Treatment failure. The proportions of patients in each arm experiencing treatment failure will be measured and compared. Treatment failure will be defined as a positive *M tuberculosis* culture of expectorated or induced sputum obtained after completion of 4 months of TB treatment, but prior to completion of TB therapy. For subjects experiencing treatment failure, drug susceptibility testing will be performed on the first *M. tuberculosis* isolate defining treatment failure. Acquired drug resistance (for 1 or more of isoniazid, rifampin, pyrazinamide, ethambutol, or fluoroquinolones) will be considered to have occurred when the initial study *M. tuberculosis* isolate is susceptible to a given drug, but the treatment failure isolate is resistant to that drug. For study purposes, “treatment success” will be defined as absence of treatment failure.

Safety and antimicrobial activity between HIV-seropositive and HIV-seronegative individuals in each treatment arm. In a subset analysis, all the endpoints (including safety and sputum culture conversion) will be evaluated to compare results in HIV-infected versus uninfected individuals.

4 STUDY DESIGN

Study Design
This will be a two-center, randomized, open-label, phase II clinical trial of an experimental versus a standard intensive phase regimen for treatment of respiratory smear-positive, culture-confirmed pulmonary TB.

Study Population
Subjects will be adult (≥ 18 years of age) males and females recruited from the Hospital Universitario Clementino Fraga Filhoof the Federal University of Rio de Janeiro, Brazil, the Núcleo de Doenças Infecciosas of the Federal University of Espitio Santo, Brazil and the Johns Hopkins Medical Institution, Baltimore, Maryland. Subjects will be either HIV-positive with CD4 ≥ 350 cells/mm^3, or HIV-negative.

This study will enroll patients with active pulmonary TB. Hospitalized and non-hospitalized persons are eligible for this study. All enrolled study subjects must have a Karnofsky score of at least 60 (requires occasional assistance but is able to care for most of his/her needs; see Inclusion Criteria). It is anticipated that the majority of enrolled subjects will be outpatients (not hospitalized).

Rationale for Study Design
Randomized controlled trials are the gold standard for assessing new therapeutic interventions. However, due to the high pill burden, it is not feasible to conduct a double-blinded study. A blinded study would increase further the number of pills required for multiple medications and placebos during the entire intensive phase for both arms – this is likely to be exceedingly difficult for patients and study personnel, and would likely compromise treatment completion (a potential problem from individual and public health perspectives). Including placebos in the study would approximately double the weekly pill burden for each participant and likely negatively impact compliance with therapy. Furthermore, Rifapentine must be stored in nitrogen blisterpacks until the time of administration, which does not allow for over-encapsulation as would be required for the use of a placebo. A rifapentine placebo in nitrogen blisterpacks is not available from the manufactuer. Lastly, the primary study endpoint is an objective laboratory endpoint (culture-conversion at 8 weeks) that would not be anticipated to be compromised by absence of patient and clinician blinding. Mycobacteriology laboratory personnel will be blinded as to the study regimen received by subjects from whom respiratory specimens are obtained.

Test Agents
The test agents are moxifloxacin and rifapentine, to be given in combination with pyrazinamide and isoniazid which are standard medications used for TB treatment. Moxifloxacin will be given orally at a dosage of 400 mg once daily, and rifapentine will be given orally at a dosage of approximately 7.5 mg/kg/dose once daily (see Table 4 for for weight-based daily dosages).

Study Arms
There will be 2 study arms:

Experimental Arm	Sample Size: 108	HPZM daily for 8 weeks† *followed by* continuation phase treatment with standard therapy for 18 or 30 weeks†
Control Arm	Sample Size: 108	HRZE daily for 8 weeks† *followed by* continuation phase treatment with standard therapy for 18 or 30 weeks†

H= isoniazid; R=rifampin; Z=pyrazinamide; E=ethambutol; P= rifapentine; M= moxifloxacin
†Pyridoxine (Vitamin B6), 50 mg daily, will be administered orally with each dose of isoniazid.

Randomization will be stratified by the presence of cavitation at baseline (time of diagnosis), since cavitation is associated with a decreased rate of 2-month culture conversion (67% vs. 85% in Tuberculosis Trials Consortium Study 22).

Following guidelines of the ATS/IDSA/CDC (Blumberg et al., 2003), duration of continuation phase TB treatment may be 30 weeks for patients having both cavitation (on initial chest x-ray) plus a positive sputum culture (for *M. tuberculosis*) at completion of intensive phase therapy. Duration of continuation phase will be 18 weeks for other patients. However, for any patient, duration of continuation phase may be extended at the discretion of the investigator.

Study enrollment
The approximate time to complete study enrollment is estimated to be 24 months.

Subject participation
The anticipated duration of participation for a typical subject is 6 months (26 weeks). This includes 8 weeks of intensive phase TB treatment, followed by 4 months (18 weeks) of standard continuation phase TB treatment. As above, following ATS/IDSA/CDC guidelines, the duration of continuation phase TB treatment may be 30 weeks (7 months, for total treatment duration of 9 months) for subjects having both cavitation on baseline chest x-ray plus a positive sputum culture for *M. tuberculosis* at completion of 8 weeks of intensive phase treatment.

Randomization strategy
Eligible patients will be randomized in a 1:1 ratio to the 2 study arms. Randomization will be stratified by lung cavitation on the chest x-ray used for screening purposes, since lung cavitation is a risk factor for treatment failure and relapse. Cavitation is defined as a gas-containing lucent space at least 1 centimeter in diameter within the lung parenchyma, surrounded by an infiltrate or fibrotic wall greater than 1 mm thick seen on a standard chest x-ray. Cavitation seen only on chest CT, if done, does not satisfy this definition. Cavities must be distinguished from pulmonary cysts, which are usually thin-walled, well-marginated lesions.

Stratified blocked randomization will be performed according to procedures set forth in the Manual of Operations. Since this is a non-blinded study, the size of the blocks will be varied randomly according to a schedule that is not known to the investigators, in order to minimize any possibility of prediction/manipulation of treatment assignment.

Outcomes
The primary outcomes of the study will be the proportion of patients in each arm with negative sputum cultures, using Lowenstein Jensen media, at completion of 8 weeks of intensive phase therapy, and safety and tolerability of the study regimens. Secondary outcomes include: time to sputum conversion, treatment failure, safety and anti-microbial activity in HIV-seropositive individuals, and PK parameters.

Data collection
Standardized data collection forms will be used to document enrollment, follow-up visits, specimen collections, laboratory results, AEs, and termination from study. Microbiologic evaluations will be performed at the Hospital Universitario Clementino Fraga Filho. All data collection forms will be completed and data entered according to instructions that will be developed in a detailed Data Management Operations Manual.

Interim Analyses
No interim efficacy analyses are planned.

Safety Oversight
An early safety evaluation will be performed when the first 40 patients (approximately 20 per arm) have completed 8 weeks of study treatment; safety data will be reviewed by the DSMB. Subsequently, the DSMB will review the study protocol and oversee progress after enrollment of every 75 participants. Special meetings of the DSMB may be called if unusual AEs are noted. The DSMB will be comprised of at least 1 expert in clinical aspects of TB, one or more biostatisticians, and one or more experts in clinical trials conduct/methodology. An independent safety monitor (a member of the DSMB) will review all SAEs immediately after they occur. The ISM may also review unanticipated or unusual AEs, at the discretion of the site PI. The DSMB will be given data grouped by study group (A or B).

5 STUDY ENROLLMENT AND WITHDRAWAL

GENERAL CONSIDERATIONS

Study subjects will be adult females and males with a presumptive diagnosis of pulmonary TB based on presence of acid-fast bacilli (AFB) (as detected by smear microscopy) in expectorated or induced sputum. Because culture confirmation is rarely available when TB treatment is initiated, patients will be recruited on the basis of having a sputum smear that is positive for AFB. In Brazil, the positive predictive value of an AFB smear for TB is > 95%.

It is anticipated that the study population will be comprised of approximately 60% males and 40% females. This reflects the slight male predominance of pulmonary TB patients at the study sites.

HIV-positive individuals with CD4 ≥ 350 cells/cu mm will be included in this study. TB is a major cause of morbidity and mortality in HIV-infected individuals, and therefore it is important to assess the antimicrobial activity, efficacy and safety of new TB treatment regimens in HIV-positive persons. Prospective studies of standard six month regimens for treatment of pulmonary TB in HIV-positive persons have shown similar treatment efficacy (as measured by time required for sputum culture conversion, treatment failure rates, and relapse rates) compared with HIV-negative persons (Perriens JH et al., 1995; El-Sadr WM et al., 1998; Vernon A et al., 1999; Kassim S et al., 1995; Chaisson RE et al., 1996).

HIV-positive individuals with CD4 < 350 cells/mm^3 will not be included in this study. Compared to HIV-negative individuals, HIV-positive persons with advanced AIDS have been shown to have a higher risk of TB relapse, including relapse with acquired rifamycin resistance when once weekly rifapentine (10 mg/kg/dose) was used during the continuation phase of isoniazid-based TB treatment (Vernon A et al., 1999). Although this problem would be anticipated to be much less likely with the use of moxifloxacin and daily rifapentine as in the current study, the potential individual and public health risks are substantial should rifamycin-resistant treatment failure occur. If the proposed experimental regimen is demonstrated to be effective and safe in this study, then subsequent future studies including individuals with advanced AIDS may be warranted. In addition, anti-retroviral therapy is an important component of care for HIV-positive individuals with CD4 < 200 cells/mm^3. Since drug-drug interactions between the study rifamycins and HIV protease inhibitors (PI's) and non-nucleoside reverse transcriptase inhibitors (NNRTI's) warrant prohibition of concomitant PI's or NNRT's while on intensive phase study therapy, individuals having CD4 < 350 cells/mm^3 may be better served by individualized (non-study) TB and HIV therapy.

Patients who, based on study laboratory testing, are newly identified to be HIV positive will receive individualized counseling about the risks and benefits of study therapy and delay of antiretroviral therapy. Individuals in whom prompt initiation of antiretroviral therapy is clinically indicated and appropriate will either be not enrolled in the study, or (if already enrolled) taken off of study medicines and treated with individualized (non-study) TB and HIV therapy.

This study will be carried out in populations characterized by very high proportions of mixed racial and ethnic groups. There will be no exclusion of racial or ethnic groups.

Children and women who are pregnant or nursing will be excluded from this study. Fluoroquinolones are relatively contraindicated in these groups. Fluoroquinolone-induced toxic cartilage reactions in the weight-bearing joints of juvenile animals have led to concern that similar AEs might result from their use in children or pregnant or nursing women. Also, Brazilian law prohibits enrollment of children in drug trials unless the drug(s) under study is for a specific pediatric indication.

This study will not enroll prisoners. However, it is possible that a participant will be incarcerated after enrollment. If this occurs during intensive phase, then study medications will be stopped and the participant will be treated for active TB according to the standards of the institution in which they are incarcerated.

There will be 108 subjects in each of the 2 treatment groups, for a total of 216 subjects. See Section 11, “Statistical Considerations” for sample size justification. This is anticipated to yield 86 evaluable subjects per treatment arm, allowing for 20% loss due to baseline drug resistance or growth of only nontuberculous mycobacteria from sputum culture.

Subject recruitment will occur through the outpatient TB clinics and inpatient ward at Hospital Universitario Clementino Fraga Filho in Rio de Janeiro, Brazil, and the Núcleo de Doenças Infecciosas of the Federal University of Espitio Santo, Brazil. Retention will be maximized through use of nurses and other study personnel. Procedures to maximize retention will include a) at enrollment (and with permission of the study participant), collection of names and contact information of friends or relatives who typically know the whereabouts of the participant, b) written appointment cards for participants, c) participants who miss a scheduled study visit may be contacted by study staff at the place of residence or through friends/relatives whose names were supplied by the participant at enrollment (the nature of the study will not be divulged to the friends/relatives), and d) reimbursement to cover travel expenses to and from the study visit, plus a light meal.

5.1 Subject Inclusion Criteria

Subjects must meet all of the following inclusion criteria in order to participate in this study:

1. Presumptive diagnosis of sputum smear-positive pulmonary TB. The sputum on which study inclusion is based must be spontaneously expectorated or induced after inhalation of nebulized hypertonic sterile saline; smear results from respiratory secretions obtained by bronchoalveolar lavage or bronchial wash may not be used for assessment of study eligibility. Sputums must be obtained no more than 14 days prior to enrollment. Patients without subsequent culture confirmation of *M tuberculosis* or patients with a *M tuberculosis* isolate resistant to 1 or more of isoniazid or rifampin or pyrazinamide or fluoroquinolones will be discontinued from the study, but followed for 14 days to detect late toxicities from study therapy. Patients having any extrapulmonary manifestations of TB except for central nervous system TB, in addition to smear-positive pulmonary disease, are eligible for enrollment.

2. Age: ≥18 years
3. Seven (7) or fewer days of multidrug therapy for TB disease in the preceding 6 months.
4. Seven (7) or fewer days of fluoroquinolone therapy in the preceding 3 months.
5. Documentation of HIV infection status. The individual must meet one of the following three criteria: a positive enzyme-linked immunosorbent assay (ELISA) and western blot at any time prior to randomization; or written documentation of a negative ELISA or western blot within the preceding 3 months or less; or the individual must consent to HIV testing.
6. For HIV seropositive individuals, a CD4 T lymphocyte count of greater than or equal to 350 cells/mm^3. Documentation of a CD4 result done at, or within the preceding 3 months or less, prior to screening is acceptable for study purposes.
7. Documentation of study baseline laboratory parameters done at, or ≤ 14 days prior to screening:
    a. Serum aspartate aminotransferase (AST) less than or equal to 2.5 times upper limit of normal.
    b. Total bilirubin level less than 2.5 times upper limit of normal.
    c. Creatinine level less than 2 times upper limit of normal.
    d. Hemoglobin level of at least 8.0 g/dl.
    e. Platelet count of at least 75,000 mm^3.
    f. Potassium level of at least 3.5.
    g. Negative pregnancy test (women of childbearing potential). If study therapy is not initiated within 72 hours of the negative pregnancy test, a urine test will be done to confirm the subject is not preganant.
8. Karnofsky score of at least 60 (requires occasional assistance but is able to care for most of his/her needs).
9. Male or nonpregnant, nonnursing female. Women with childbearing potential must agree to practice, during intensive phase, one or more of the following methods of birth control: intrauterine device; barrier methods including the diaphragm or cervical cap (each used with spermicidal foam or jelly), condom, or sponge; or abstinence from heterosexual intercourse.
10. Provision of informed consent.

5.2 Subject Exclusion Criteria

All subjects meeting any of the following exclusion criteria at baseline will be excluded from study participation:

1. CD4 count < 350 cells/cu mm.

2. Presence of active AIDS-related opportunistic infection (other than TB) or active AIDS-related malignancy.
3. Known intolerance to any of the study drugs.
4. Concomitant disorders or conditions for which any of the study drugs is contraindicated. These include severe hepatic damage, acute liver disease of any cause, and acute uncontrolled gouty arthritis.
5. Inability to take oral medication.
6. Central nervous system TB.
7. Pulmonary silicosis.
8. Current or planned therapy, during study phase (intensive phase of TB treatment), with any one or more of the following drugs: quinidine, procainamide, amiodarone, sotalol, disopyramide, terfenadine, cisapride, erythromycin, clarithromycin, phenothiazines, haloperidol, olanzapine, ziprasidone, tricyclic antidepressants, corticosteroids administered either orally or intravenously, fluconazole, itraconazole, ketoconazole, oral or intravenous tacrolimus, oral or intravenous cyclosporine, HIV protease inhibitor, HIV non-nucleoside reverse transcriptase inhibitor, carbamazepine, phenytoin, theophylline.
9. Concurrent severe and/or uncontrolled medical or psychiatric condition that, in the opinion of the investigator, could cause unacceptable safety risks or compromise compliance with the protocol.
10. Unable or unwilling to receive directly observed therapy and/or adhere with follow-up (e.g. due to residence remote from the study site).
11. Refusal of consent.

5.3 Treatment Assignment Procedures

5.3.1 Randomization Procedures

This will be a randomized trial. Randomization will be stratified by the presence of cavitation at baseline (time of diagnosis), since cavitation is associated with a decreased rate of 2-month culture conversion (67% vs. 85% in Tuberculosis Trials Consortium Study 22). Randomization will be computer generated.

This will be an open-label trial in which neither the subjects nor the study staff will be blinded as to treatment assignment after randomization.

Informed consent will be obtained by trained study personnel prior to screening for inclusion and exclusion criteria. Eligible patients (who meet all of the inclusion criteria and none of the exclusion criteria) will be randomized in a 1:1 ratio to the 2 study arms.

5.3.2 Reasons for Withdrawal

Subjects may withdraw voluntarily from participation in the study at any time upon request. Subjects may also withdraw voluntarily from receiving the study intervention for any reason.

Criteria for temporary discontinuation of study drugs:

- Any clinical AE or laboratory abnormality that, depending on its nature and severity, requires temporary discontinuation of the study drugs until the toxicity resolves as indicated in the subsequent toxicity management section (Section 9.1.3).
- An intercurrent illness, other medical condition or situation occurs such that continued administration of study drugs and/or participation in the study would not be in the best interest of the subject.
- Development of any exclusion criteria may be cause for discontinuation.

The subject will continue to be followed with the subject's permission if study intervention/product is discontinued.

As described above in "Inclusion Criteria" patients without subsequent culture confirmation of *M. tuberculosis* or patients with a *M. tuberculosis* isolate resistant to 1 or more of isoniazid or rifampin or pyrazinamide or fluoroquinolones will be discontinued from the study, but followed for 14 days to detect late toxicities from study therapy.

As described above in Section 5, this study will not enroll prisoners. However, it is possible that a participant will be incarcerated after enrollment. If this occurs during intensive phase, then study medications will be stopped and the participant will be treated for active TB according to the standards of the institution in which they are incarcerated.

In the event of study closure by the DSMB, study subjects on intensive phase TB treatment will be discontinued from their assigned study regimen. Subjects will be treated with a standard regimen for TB according to Brazilian guidelines.

5.3.3 Handling of Withdrawals

Attempts to contact participants who fail to follow-up in the study will include at least 1 telephone call and 1 home visit, if appropriate. Interviews with patients who withdraw from the study will focus on the reason for discontinuation and the development of adverse reactions. If voluntary withdrawal occurs, the subject will be asked to continue scheduled evaluations.

Early termination during the intensive phase of TB treatment: if the subject is willing, a termination visit will be performed. Assessments will be performed according to the Schedule of Procedures/Evaluations, Appendix A.

Early termination during the continuation phase of TB treatment: if the subject is willing, a termination visit will be performed. Assessments will be performed according to the Schedule of Procedures/Evaluations, Appendix A.

If voluntary withdrawal occurs, subjects will be given appropriate care under medical supervision until the symptoms of any AE resolve. Subjects receiving TB treatment will be referred to the municipal TB clinic or hospital outpatient TB program for care.

Replacement of individual participants who discontinue early is not allowed. The planned enrollment target allows for 20% of participants to be unevaluable for the primary endpoint.

5.3.4 Triggers for Review

Enrollment will be temporarily halted (see Section 9.4 “Halting Rules”) and a formal DSMB safety review will be undertaken if either or both of the following occur:

a) 3 or more SAEs attributed to study therapy in the experimental HPZM arm

b) 6 or more participants with grade 3 or higher hepatotoxicity in the experimental HPZM arm

6 STUDY INTERVENTION/INVESTIGATIONAL PRODUCT

Moxifloxacin and rifapentine are the investigational products and are described in detail below.

Isoniazid, rifampin, pyrazinamide, and ethambutol will also be used in accordance with published guidelines (Blumberg 2003) and FDA approvals. Pyridoxine (vitamin B6) will be used in accordance with published guidelines (Blumberg 2003) and FDA recommendations. Isoniazid, rifampin, pyrazinamide, ethambutol, and pyridoxine, manufactured under Good Manufacturing Practice Regulations, will be provided to the Brazil site by the TB program of the Hospital Universitario Clementino Fraga Filho or purchased from legal distributors. Manufacturer package inserts for moxifloxacin, isoniazid, rifampin, rifapentine, pyrazinamide, ethambutol, and pyridoxine will be distributed to study investigators.

6.1 Study Product Description (Moxifloxacin)

Moxifloxacin is approved for use by the US Food and Drug Administration (FDA) for the following indications: a) acute bacterial exacerbation of chronic bronchitis (caused by *Streptococcus pneumoniae, Haemophilus influenzae, Haemophilus parainfluenzae, Klebsiella pneumoniae, Staphylococcus aureus,* or *Moraxella catarrhalis*); b) acute bacterial sinusitis (caused by *Streptococcus pneumoniae, Haemophilus influenzae,* or *Moraxella catarrhalis*); c) community-acquired pneumonia (caused by *Streptococcus pneumoniae, Haemophilus influenzae, Moraxella catarrhalis, Staphylococcus aureus, Klebsiella pneumoniae, Mycoplasma pneumoniae,* or *Chlamydia pneumoniae*); and d) uncomplicated skin and skin structure infections (caused by *Staphylococcus aureus* or *Streptococcus pyogenes*).

In this study, moxifloxacin will be used for an off-label indication, in combination with other medications FDA-approved for use for treatment of pulmonary TB.

6.1.1 Acquisition

Moxifloxacin, manufactured as AVELOX (moxifloxacin hydrochloride) by Bayer Pharmaceuticals, will be obtained from Bayer Pharmaceuticals. Film-coated tablets are oblong and dull red color. Tablets are coded with the word “BAYER” on 1 side and “M400” on the reverse.

6.2 Study Product Description (Rifapentine)

Rifapentine is approved for use by the US FDA for the treatment of TB. The recommended dose of rifapentine is 600 mg twice per week during intensive phase of TB treatment, and 600 mg once per week during continuation phase of TB treatment.

In this study, rifapentine will be used to treat pulmonary TB during the intensive phase of therapy at a lower dose but more frequent dosing interval (i.e. approximately 7.5 mg/kg dose given once daily; see Table 4 for weight-based daily dosages) than recommended by the FDA.

In this study, rifapentine will be used in combination with moxifloxacin, pyrazinamide, and ethambutol.

6.2.1 Acquisition

Rifapentine, manufactured as PRIFTIN by Sanofi-Aventis, will be obtained from Sanofi-Aventis. Tablets are film coated, pink, round-shaped, and are stamped with PRIFTIN 150 on one side.

6.2.2 Product Storage and Stability

All study drugs will be stored according to procedures set forth in a detailed Manual of Operating Procedures. Storage temperature will be room temperature, about 25ºC, with excursions from 15ºC to 30º permitted. Temperature will be checked and recorded per study SOPs. Only designated study personnel will have access to study drugs. Study drugs will not be used beyond the manufacturer's recommended expiration date.

6.3 Dosage, Preparation and Administration of Study Intervention/Investigational Product

All study drugs will be administered orally (by mouth), and all are formulated as tablets or capsules that are ready for administration without special preparation.

During intensive phase, therapy (control arm and experimental arm) will be given as directly observed therapy administered by study personnel on weekdays, and will be self-administered on weekends using pre-packaged study medicines. On government holidays, intensive phase therapy may be directly observed or self-administered. Directly observed therapy may be given at the TB clinic or other health care facility, or, with the participant's permission, at the participant's residence, workplace, or other mutually agreed upon location convenient for the participant. Subjects will have from 56 to 71 days to complete the intensive phase of therapy.

The standard control intensive phase regimen will consist of the following: isoniazid, rifampin, pyrazinamide, and ethambutol (HRZE) administered daily for 8 weeks. Pyridoxine (vitamin B6) will be given with each dose of isoniazid. For the control arm, the end of intensive phase is defined as completion of 56 doses.

The experimental intensive phase regimen will consist of the following: isoniazid, rifapentine, pyrazinamide, and moxifloxacin (HPZM) administered daily, for 8 weeks. Pyridoxine (vitamin B6) will be given with each dose of isoniazid. The end of intensive phase is defined as completion of 56 doses.

Table 4. Doses of Study Medications during Intensive Phase

DRUG	Dose
Moxifloxacin	400 mg
Rifampin	
≤ 45 kg	450 mg
> 45 kg	600 mg
Rifapentine	
≤ 45 kg	300 mg
> 45 kg	450 mg
Isoniazid	
≤ 40 kg	200 mg
> 40 kg	300 mg
Pyrazinamide[1]	
< 40 kg	25 mg/kg rounded to nearest 250 mg
40 – 55 kg	1000 mg
56 – 75 kg	1500 mg
> 75 kg	2000 mg
Ethambutol[1]	
< 40 kg	20 mg/kg rounded to nearest 100 mg
40-55 kg	800 mg
56-75 kg	1200 mg
> 75 kg	1600 mg
Vitamin B6 (pyridoxine)	50 mg

[1] Per ATS/CDC/IDSA guidelines, 2003

6.4 Modification of Study Intervention/Investigational Product for a Participant

Dose/Schedule Modifications for a Subject
Dose modifications are anticipated to be infrequent.

In the event of study subject weight change during intensive phase, the doses of isoniazid, rifampin, pyrazinamide, and ethambutol will be modified, as needed, based on Table 4. Dose modifications for instances other than weight change are not anticipated.

6.5 Accountability Procedures for the Study Intervention/Investigational Product(s)

The study physician attending the patient will write a prescription for the study medications, which is sent to the study pharmacy. All medications received by study participants will be distributed by the study pharmacist to the study nurses. Each patient is assigned a pack of medications which are distributed to study staff per study SOPs and are administered to the patient as described in section 6.3.

Moxifloxacin will be distributed to the study pharmacy by Bayer Brazil. Rifapentine will be shipped to the study pharmacy by Johns Hopkins at the beginning of the study. Enough medication for the duration of the study will be provided at the beginning if possible, based on expiration dates of the drugs. Standard therapy used during the continuation phase will be obtained from the TB clinic at the hospital and will be distributed from the study pharmacy. Unused or expired medications will be destroyed per local guidelines. Documentation of all drugs received, distributed and destroyed will be kept by the study pharmacist. Investigational products will be tracked on a product accountability form.

6.6 Assessment of Subject Compliance with Study Intervention/Investigational Product

During study phase of therapy, all medications will be administered by DOT by study personnel on weekdays and will be self-administered on weekends. A record of medication administration will be maintained for assessment of study subject compliance.

6.7 Concomitant Medications/Treatments

The use of all non-study drugs (prescription and over-the-counter medications) during the study will be monitored and recorded.

Quinidine, procainamide, amiodarone, sotalol, disopyramide, terfenadine, cisapride, erythromycin, clarithromycin, phenothiazines, haloperidol, olanzapine, ziprasidone, or tricyclic antidepressants: these medications, like moxifloxacin, can prolong the electrocardiographic QT and QTc intervals and should not be administered during intensive phase of study treatment. If, during the intensive phase, a participant in either treatment arm requires ongoing treatment with, or receives more than 3 days of any of these medications, the participant will be taken off

of study medication, and treated with an individualized TB regimen. Subjects treated outside of the study will continue to be followed and treated by the study team, per the study schedule, unless the patient requests otherwise or withdraws consent.

Corticosteroids (oral or intravenous), fluconazole, itraconazole, ketoconazole, oral or intravenous tacrolimus, oral or intravenous cyclosporine, HIV protease inhibitor, HIV non-nucleoside reverse transcriptase inhibitor: rifampin and rifapentine induce the metabolism of hepatic enzyme systems that are active in drug metabolism. Rifampin and rifapentine increase the clearance of corticosteroids, azole antibiotics, tacrolimus, cyclosporine, HIV protease inhibitors, and HIV non-nucleoside reverse transcriptase inhibitors. Concommitant use of rifampin or rifapentine with these medications may therefore diminish the therapeutic efficacy of corticosteroids, azole antibiotics, tacrolimus, cyclosporine, HIV protease inhibitors, and HIV non-nucleoside reverse transcriptase inhibitors. If, during the intensive phase, a participant in either treatment arm requires ongoing treatment with, or receives more than 14 days of any of these medications, the participant will be taken off of study medication, and treated with an individualized TB regimen.

Isoniazid decreases the clearance of some medications that are metabolized in the liver. Participants receiving isoniazid who require carbamazepine, phenytoin, or theophylline will have serum drug levels measured and monitoring for clinical side effects at intervals deemed appropriate by the investigator.

Rifampin and rifapentine induce a number of hepatic enzyme systems that are active in drug metabolism. While on study phase, women of childbearing potential (ie, not surgically sterilized or not postmenopausal for > 1 year) will be advised to use 1 or more of the following contraceptive methods: intrauterine device; barrier methods including the diaphragm or cervical cap (each used with spermicidal foam or jelly), condom, or sponge; or abstinence from heterosexual intercourse. Due to drug-drug interactions between rifamycins and hormonal contraceptives, the use of a hormonal contraceptive as a sole means of contraception is not adequate; hormonal contraceptives may be continued but must be supplemented by one of the contraceptive methods listed above. Due to the multiple interactions of rifampin and rifapentine with antiretroviral therapy, HIV-infected patients receiving or anticipated to receive treatment with an HIV protease inhibitor and/or an HIV non-nucleoside reverse transcriptase inhibitor during the intensive phase of TB treatment will not be eligible for the study. Patients who initiate HIV therapy with a protease inhibitor and/or non-nucleoside reverse transcriptase inhibitor during intensive phase therapy will be taken off the study, and treated with an individualized TB regimen.

Antacids that contain aluminum and/or magnesium, and antidiarrheals that contain kaolin can decrease absorption of isoniazid, ethambutol, and moxifloxacin. Participants who require the use of antacids containing aluminum and/or magnesium, sucralfate, antidiarrheals that contain kaolin, iron-containing medications or supplements, or zinc-containing medications or supplements will need to take their study medications at least 4 hours before or 8 hours after ingesting these products to avoid impaired absorption of study drugs.

Ethanol can exacerbate the potential hepatotoxicity of isoniazid, rifampin, rifapentine, and pyrazinamide. Participants will be urged to abstain from alcohol while on TB treatment.

7 STUDY SCHEDULE

7.1 Screening

Recruitment will occur through the outpatient TB clinic and inpatient ward at Hospital Universitario Clementino Fraga Filho, the Núcleo de Doenças Infecciosas , and the Johns Hopkins Medical Institution. All sites have existing pathways for the referral of TB patients from outlying TB clinics. Subjects with a presumptive diagnosis of sputum smear positive pulmonary TB will be invited to participate. Because culture confirmation of TB is rarely available when TB treatment is initiated, patients will be recruited on the basis of a positive sputum smear. In Brazil, the positive predictive value of an AFB smear for TB is > 95%. Interested individuals who are invited to participate will undergo screening to determine eligibility for enrollment.

The screening process will include the informed consent process. Informed consent is a process that will be initiated prior to the individual's agreeing to participate in the study and will continue throughout the individual's study participation. Extensive discussion of risks and possible benefits of this therapy will be provided by designated study staff to potential subjects (and, with permission from potential subjects, their families). Potential subjects will receive counseling about study objectives and procedures, potential toxicities, and the informed consent process. Consent forms describing in detail the study interventions/products, study procedures and risks will be given to the subject and written documentation of informed consent will be required prior to starting intervention/administering study product. Consent forms will be IRB approved, and written in Portuguese (an English version of the Portuguese consent form will be available for IRB and sponsor review). The subject will be asked to read and review the document. Upon reviewing the document, the person obtaining consent will explain the research study to the subject and answer any questions that may arise. For subjects who are unable to read or write, all of the information in the consent form will be communicated verbally, in the presence of an adult witness who is not a member of the study team; informed consent requires the signature or mark of the subject. The subject will sign or mark the informed consent document prior to any procedures being done specifically for the study. The subjects will have the opportunity to discuss the study with their surrogates or think about it prior to agreeing to participate. A copy of the signed and dated informed consent document will be given to the subjects for their records. The rights and welfare of the subjects will be protected by emphasizing, to subjects, that the quality of their medical care will not be adversely affected if they decline to participate in this study. For individuals who choose not to participate in the study, treatment with the Brazilian Ministry of Health standard TB treatment regimen will be offered as an alternative, non-study regimen in Brazil; these individuals will be referred to the appropriate municipal health department TB clinic or the hospital TB program. The subjects may withdraw consent at any time throughout the course of the trial.

Screening evaluations to be done are listed in the Schedule of Procedures/Evaluations (Appendix A), and detailed in Section 8 (Study Procedures/Evaluations).

Screening should be done as soon as possible after identification of an interested individual having a presumptive diagnosis of sputum smear positive pulmonary TB, since no more than 7

days of multidrug therapy for TB in the preceding 6 months are allowed prior to initiation of the first dose of study medications.

7.2 Enrollment/Baseline

When results of blood tests, pregnancy test (if applicable), and chest radiograph are available, and if the patient meets enrollment criteria based on screening evaluations and has provided informed consent, then information about current medications will again be collected to confirm that the subject still meets eligibility criteria with respect to concomitant medications.

If the subject still meets eligibility criteria, then he/she will be randomized as described above in Section 5.3.1 “Randomization Procedures”.

Participants will be started on their assigned study therapy as soon as possible, but within 7 days or fewer, after randomization.

7.3 Follow-up

The evaluations to be done during study visits during Intensive Phase and Continuation Phase are listed in the Schedule of Procedures/Evaluations (Appendix A), and detailed in Section 8 (Study Procedures/Evaluations).

Intensive phase of TB therapy:
Study visits will occur at weekly after enrollment.

Continuation phase of TB therapy
After completing the intensive phase of therapy, patients in both study arms will then be treated with a continuation-phase regimen according to guidelines of the ATS/IDSA/CDC (Blumberg et al., 2003). Recommended continuation-phase regimens include:

isoniazid + rifampin given daily, thrice-weekly, or twice-weekly

rifampin + pyrazinamide + ethambutol for patients who are intolerant of isoniazid (note: patients with known isoniazid intolerance cannot be enrolled in the protocol, but patients may develop isoniazid intolerance during study therapy).

For patients intolerant of above regimens, treatment may be with an individualized regimen at the discretion of the investigator.

The total duration of therapy will be 26 weeks (8 weeks of intensive phase plus 18 weeks of continuation phase), except that patients who have the combination of cavitation (on baseline chest radiograph) plus a positive sputum culture at the end of the intensive phase of therapy may receive a total of 38 weeks (8 weeks of intensive phase plus 30 weeks of continuation phase) of therapy. In addition, duration of intensive phase may be increased for any patient at the discretion of the investigator.

During continuation phase, scheduled study visits will occur at 12, 16, 20, and 26 weeks after enrollment. For study subjects whose treatment is extended to 38 weeks of total therapy, additional scheduled study visits will occur at 30, 34, and 38 weeks. Assessments to be performed at continuation phase visits are listed in the Schedule of Procedures/Evaluations (Appendix A) and described in detail in Section 8.

7.4 Final Study Visit

The final study visit should occur at the time of completion of continuation phase TB therapy, or within 14 days of completion of continuation phase TB therapy. Assessments to be performed at the final study visit are listed in the Schedule of Procedures/Evaluations (Appendix A) and described in detail in Section 8. When a person completes study follow-up successfully, the Study Completion Form will be completed.

7.5 Early Termination Visit

Subjects may withdraw voluntarily from participation in the study at any time. Subjects may also withdraw voluntarily and for any reason from receiving the study intervention. If voluntary withdrawal occurs, the subject will be asked to continue scheduled evaluations.

Early termination during the intensive phase of TB treatment: if the subject is willing, a termination visit will be performed. Assessments to be performed at the early termination visit (intensive phase) are listed in the Schedule of Procedures/Evaluations (Appendix A) and described in detail in Section 8.

Early termination during the continuation phase of TB treatment: if the subject is willing, a termination visit will be performed. Assessments to be performed at the early termination visit (continuation phase) are listed in the Schedule of Procedures/Evaluations (Appendix A) and described in detail in Section 8.

If voluntary withdrawal occurs, subjects will be given appropriate care under medical supervision until the symptoms of any AE or SAE resolve or the subject's condition becomes stable. Subjects on TB treatment will be referred to the municipal TB clinic or the hospital TB program for care.

When a person discontinues study follow-up for a reason other than study completion, the Study Termination Form will be completed.

7.6 Unscheduled Visit

A targeted evaluation addressing a participant's chief complaint will be performed during unscheduled visits. Participants will be asked about the use of concomitant medications, AEs, compliance, and symptoms of TB. Laboratory evaluation to address drug toxicity or worsening symptoms of TB will be performed based on the nature and grading of any AE. The evaluating clinician will be asked to complete a data collection form to document the details of the encounter.

8 STUDY PROCEDURES/EVALUATIONS

8.1 Clinical Evaluations

Screening Clinical Evaluations

- Informed consent process (see details in Section 7.1).
- Medical history obtained from medical records and interview: Symptoms of TB, time of current TB diagnosis, co-morbidities, past medical history including prior treatment for latent or active TB.
- Demographics including place of current residence and race/ethnicity.
- Physical examination: vital signs (blood pressure, temperature and respiratory rate per routine) including height, weight, and heart rate.
- Medications: currently used medications (prescription and over-the-counter), and medications used during the preceding 14 days.
- Symptom assessments: Participant will be queried about the following: fevers, sweats, cough, rash, itching, jaundice, abdominal discomfort or pain, nausea, vomiting, diarrhea, loss of appetite, bad taste in mouth, vision problems, numbness/tingling of extremities, headache, dizziness, insomnia, joint pain, palpitations, syncope.
- Visual testing (Snellen test for acuity, and Ishihara testing for color vision).
- Counseling about study objectives and drug toxicity.

Baseline Clinical Evaluations

- Medications: currently used medications (prescription and over-the-counter), and medications used since the screening evalution.
- Counseling about drug toxicity.
- Pregnancy: women of child-bearing potential should be asked if they may be pregnant, date of last menstrual period, and offered a pregnancy test (urine or serum) if they think they could be pregnant. If a repeat pregnancy test is performed, initiation of study drugs should be postponed until results of this pregnancy test are available; if results are positive (for pregnancy), then that individual no longer meets study eligibility criteria.

Clinical Evaluations during Intensive Phase of TB therapy (timing of evaluations is indicated in the Schedule of Procedures/Evaluations, Appendix A)

- Medications: all medications taken since the preceding visit (prescription and over-the-counter).
- Physical examination: vital signs including weight and heart rate.
- Symptom assessments: Participant will be queried about the following: fevers, sweats, cough, rash, itching, jaundice, abdominal discomfort or pain, nausea, vomiting, diarrhea, loss of appetite, bad taste in mouth, vision problems, numbness/tingling of extremities, headache, dizziness, insomnia, joint pain, palpitations, syncope.

- Visual testing (Snellen test for acuity, and Ishihara testing for color vision) at weeks 4 and 8.
- Counseling about drug toxicity.
- Adverse Event assessment
- Pregnancy: At week 1, 2, 3, 5, 6, 7 and 8 study visits, women of child-bearing potential should be asked if they may be pregnant, date of last menstrual period, and offered a pregnancy test (urine or serum) if they think they could be pregnant. At week 4, women of child-bearing potential should be asked if they may be pregnant, date of last menstrual period, and a serum pregnancy test will be performed.
- Interval medical history.

Clinical Evaluations during Continuation Phase of TB therapy (timing of evaluations is indicated in the Schedule of Procedures/Evaluations, Appendix A)

- Medications: all medications taken since the preceding study visit (prescription and over-the-counter).
- Physical examination: vital signs including weight and heart rate.
- Symptom assessments: Participant will be queried about the following: fevers, sweats, cough, rash, itching, jaundice, abdominal discomfort or pain, nausea, vomiting, diarrhea, loss of appetite, bad taste in mouth, vision problems, numbness/tingling of extremities, headache, dizziness, insomnia, joint pain, palpitations, syncope.
- Visual testing (Snellen test for acuity, and Ishihara testing for color vision) (Week 12 only).
- Counseling about drug toxicity.
- Adverse Event assessment
- Interval medical history.

Clinical Evaluations at Final Study Visit

The final study visit should occur at the time of completion of continuation phase TB therapy (see Section 7.4). The following assessments will be performed:

- Medications: all medications taken since the preceding study visit (prescription and over-the-counter)
- Physical examination: vital signs including weight and heart rate.
- Symptom assessments: Participant will be queried about the following: fevers, sweats, cough, rash, itching, jaundice, abdominal discomfort or pain, nausea, vomiting, diarrhea, loss of appetite, bad taste in mouth, vision problems, numbness/tingling of extremities, headache, dizziness, insomnia, joint pain, palpitations, syncope.
- Counseling about drug toxicity
- Adverse event assessment
- Interval medical history.

Clinical Evaluations at Early Termination Visit

- If early termination occurs during intensive phase of TB treatment, and if the subject is willing, then the following clinical evaluations should be performed:
- Medications: all medications taken since the preceding visit (prescription and over-the-counter).
- Physical examination: vital signs including weight and heart rate.
- Symptom assessments: Participant will be queried about the following: fevers, sweats, cough, rash, itching, jaundice, abdominal discomfort or pain, nausea, vomiting, diarrhea, loss of appetite, bad taste in mouth, vision problems, numbness/tingling of extremities, headache, dizziness, insomnia, joint pain, palpitations, syncope.
- Visual testing (Snellen test for acuity, and Ishihara testing for color vision), to be done if early termination occurs prior to the week 12 study visit.
- Counseling about drug toxicity.
- Adverse Event assessment
- Pregnancy: if early termination occurs prior to the week 12 study visit, then women of child-bearing potential should be asked if they may be pregnant, date of last menstrual period, and offered a pregnancy test (urine or serum) if they think they could be pregnant.
- Interval medical history.

Other clinical management issues

HIV management: HIV testing will be done by trained personnel who use approved procedures for pre-test and post-test counseling. HIV testing will be performed using a method approved by regulatory agencies for use in each respective country. HIV test results will be given only to the patient (and to those to whom it may be required by law) and kept with other study records in a secure place. CD4 tests will be performed in accordance with section 8.2.1, and results will be given to patients as soon as they are available. HIV-positive patients will be referred to local sources of HIV care, as appropriate.

The protocol does not allow the use of antiretroviral therapy during the intensive phase of therapy, because of overlapping side effect profiles of anti-TB and antiretroviral drugs, the adherence challenge of starting so many drugs in a short period of time, the complex drug-drug interactions between rifamycins and many antiretroviral drugs (most HIV-1 protease inhibitors and non-nucleoside reverse-transcriptase inhibitors) (Burman et al. 1999), and the possible occurrence of severe immune reconstitution syndromes when antiretroviral therapy is started early in the course of TB treatment (Burman et al. 2001). Patients whose clinical condition is thought to require antiretroviral therapy during the intensive phase of TB treatment will not be enrolled (see Exclusion criteria).

Management of patients with TB treatment failure: TB treatment failure is defined as a positive *M tuberculosis* culture obtained after completion of 4 months of therapy. Patients with treatment failure will be withdrawn from the study and treated with an individualized TB regimen. For each patient with treatment failure, drug susceptibility testing will be performed for 1 *M.*

tuberculosis isolate from sputum obtained after completion of 4 months of therapy. This drug susceptibility test result will be compared with that of the patient's initial isolate.

Rifamycin Hypersensitivity: Case definition (potential hypersensitivity syndrome): any participant who, after starting study drug treatment but before or at the week 12 study visit, develops a grade 3 or higher adverse event that is considered by the local investigator to be attributed to study drugs and for which there is no other known explanation. For participants meeting the case definition the following tests should be obtained at the first opportunity after the patient develops symptoms (acute phase) AND then again at 10-35 days after recovery from the event (convalescent phase): serum alanine aminotransferase (ALT), serum total bilirubin, serum creatinine, complete blood count (CBC), 10 cc of blood collected in a red top tube, clotted 30-60 minutes, centrifuged and stored frozen at -50˚C or colder in a plastic tube.

8.2 Laboratory Evaluations

8.2.1 Clinical Laboratory Evaluations

<u>Screening Laboratory Evaluations</u>

- Hematology: hemoglobin, platelet count, absolute neutrophil count (5 mL EDTA anticoagulated blood). These tests should be done at the time of screening unless results from evaluations done within the preceding 14 days or less are available.
- Biochemistry: AST, total bilirubin, creatinine, potassium (5 mL whole blood for serum). These tests should be done at the time of screening unless results from evaluations done within the preceding 14 days or less are available.
- HIV test: any methodology approved by Brazilian regulatory authorities for use in Brazil, including blood ELISA (5 mL blood). An HIV test must be performed unless written results of a positive HIV test (done at any time) are available, or written results of a negative test performed within the preceeding 3 months or less are available.
- CD4 count: FLOW cytometry (5 mL blood). CD4 enumeration will be performed only for HIV-positive subjects, and not for HIV-negative subjects. For HIV-positive subjects, enumeration of CD4 T cells will be performed unless written results are available from a test done within the preceding 3 months or less.
- Pregnancy test (5 mL whole blood for serum), to be performed on women of childbearing potential, and to be done at the time of initial screening. If female subjects are not started on study medications within 72 hours of their screening pregnancy test, a urine pregnancy test will be done to confirm they are not pregnant.
- Chest x-ray: a standard posterior-anterior and lateral chest x-ray will be performed by a trained technologist using equipment that meets local certification requirements. A chest x-ray will be performed unless a radiograph done within the preceding 14 days or less is available for review by study personnel (a written report is not acceptable). Prior to performing a chest x-ray, women of childbearing potential should be questioned as to the possibility of pregnancy. If, based on questions, pregnancy cannot be reasonably excluded, a negative pregnancy test should be confirmed prior to performance of a chest x-ray.

- Sputum obtainment and examinations: one sputum specimen will be obtained unless one has been obtained and sent to the study laboratory within the preceding 3 days or less. Sputum may be obtained either by spontaneous expectoration or by induction (through inhalation of nebulized hypertonic sterile saline). For purposes of determining study eligibility, sputum that is spontaneously expectorated or induced is acceptable; respiratory secretions obtained during bronchoalveolar lavage or bronchial wash are not acceptable. AFB smear microscopy and culture will be performed on the sputum specimen. For AFB smear microscopy, Ziehl-Neelsen, Kinyoun, or auramine/rhodamine staining will be used to stain concentrated or non-concentrated sputum specimens. For culture, the sputum specimen will be processed using conventional methodology, and cultured on Lowenstein Jensen medium. For each participant, drug susceptibility testing will be performed on the baseline isolate, as well as the first treatment failure isolate (if applicable). Drug susceptibility testing will include susceptibility to isoniazid, rifampin, and ethambutol.
- 10 cc of blood should be collected in a red top tube, clotted 30-60 minutes, centrifuged and serum frozen at -50˚C or colder in a plastic tube.

Laboratory Evaluations during Intensive Phase (timing of evaluations is indicated in the Schedule of Procedures/Evaluations, Appendix A)

- One sputum sample will be obtained (for AFB smear and culture) at each study visit at weeks 1 - 7. If the participant is unable to spontaneously expectorate a specimen at any of these times, an attempt will be made to induce sputum production by aerosol inhalation of nebulized hypertonic sterile saline. A sputum specimen will be considered unobtainable (and negative for analytic purposes) if no sputum can be obtained after one attempt at sputum induction.
- Two sputum specimens will be obtained for AFB smear and culture at completion of intensive phase treatment (week 8). These specimens must be obtained on the day of completing intensive phase therapy or up to 7 days after completion of intensive phase therapy, but before more than one dose of continuation phase therapy has been given. The two sputum specimens can be obtained on the same day. Expectorated and/or induced sputums are acceptable. If the participant is unable to spontaneously expectorate a specimen, then an attempt will be made to induce sputum production by aerosol inhalation of nebulized hypertonic sterile saline. A sputum specimen will be considered unobtainable (and negative for analytic purposes) if no sputum can be obtained after one attempt at sputum induction.
- Hematology: determinations of hemoglobin, platelet count, and absolute neutrophil count will be performed at study visits at weeks 2, 4, 6, and 8.
- Biochemistry: serum creatinine, bilirubin, and AST assessments will be performed at study visits at weeks 2, 4, 6, and 8.
- Pregnancy test (5 ml whole blood for serum) for women of child-bearing potential at week 4.
- At week 8: 10 cc of blood should be collected in a red top tube, clotted 30-60 minutes, centrifuged and serum frozen at -50˚C or colder in a plastic tube.

Laboratory Process for Population PK sampling

- Population PK sampling will be performed only for participants allocated to the experimental (rifapentine plus moxifloxacin-containing) arm.
- PK blood sampling will be done at approximately study day 21, by which time steady state and maximal enzyme induction have been achieved. As weekday doses are taken by DOT, the PK sampling will be collected Tuesday – Friday
- For each participant, immediately prior to administering study medicines, 10 cc of blood will be collected in a green top (sodium plus heparin) vacutainer, and plasma will be prepared and stored immediately according to a procedures manual.
- The patient study ID number, date and exact time of the blood draw, and the date and time of the preceding medication dose will be recorded.
- The study medications will be administered according to protocol and the exact time recorded.
- 10cc of blood will then be collected in green top tubes at 45 minutes, 1.5 hours, 4 hours, and 24 hours after study medicines have been given.
- Plasma will be stored frozen at -50C or colder on-site in Brazil, and then shipped in batches to the Infectious Disease Pharmacokinetics Laboratory under the direction of Dr. Charles Peloquin.

Laboratory Evaluations during Continuation Phase (timing of evaluations is indicated in the Schedule of Procedures/Evaluations, Appendix A)

- One sputum sample will be obtained (for AFB smear and culture) at the week 12 study visit. If the participant is unable to spontaneously expectorate a specimen at week 12, then for study purposes the sputum specimen will be considered unobtainable (and negative for analytic purposes).
- After week 12, one spontaneously expectorated sputum sample will be obtained (for AFB smear and culture) at each subsequent scheduled study visit until two previously collected study sputums are culture negative for *M. tuberculosis*.
- Hematology: determinations of hemoglobin, platelet count, and absolute neutrophil count will be performed at the week 12 study visit.
- Biochemistry: serum creatinine, bilirubin, and ASTassessments will be performed at the week 12 study visit.

Laboratory Evaluations at Final Study Visit

- One spontaneously expectorated sputum sample should be obtained (for AFB smear and culture) unless two previously collected study sputums are culture negative for *M. tuberculosis*. If the participant is unable to spontaneously expectorate a specimen, then for study purposes the sputum specimen will be considered unobtainable (and negative for analytic purposes).

- Chest x-ray: a standard posterior-anterior and lateral chest x-ray will be performed by a trained technologist. One chest x-ray should be obtained for study purposes; this x-ray is intended to be an end-of-treatment x-ray. Therefore, for individuals in whom TB treatment IS NOT extended, the chest x-ray should be performed at week 26. For individuals in whom TB treatment IS extended, the chest x-ray should be performed at week 38.

Laboratory Evaluations at Early Termination

If early termination occurs during intensive phase of TB treatment, and if the subject is willing, then the following laboratory evaluations should be performed:

- Two sputum specimens will be obtained for AFB smear and culture. These specimens must be obtained on the day of early termination or up to 7 days after early termination, but before more than one dose of non-study TB therapy has been given. The two sputum specimens can be obtained on the same day. Expectorated and/or induced sputums are acceptable. If the participant is unable to spontaneously expectorate a specimen, then an attempt will be made to induce sputum production by aerosol inhalation of nebulized hypertonic sterile saline. A sputum specimen will be considered unobtainable (and negative for analytic purposes) if no sputum can be obtained after an attempt at sputum induction.
- Hematology: determinations of hemoglobin, platelet count, and absolute neutrophil count will be performed.
- Biochemistry: serum creatinine, bilirubin, and AST assessments will be performed.

If early termination occurs during continuation phase of TB treatment and if the subject is willing, then the following laboratory evaluations should be performed:

- One spontaneously expectorated sputum sample should be obtained (for AFB smear and culture) unless two previously collected study sputums are culture negative for *M. tuberculosis*.
- Chest x-ray: a standard posterior-anterior and lateral chest x-ray will be performed by a trained technologist using equipment that meets local certification requirements.
- Determinations of hemoglobin, platelet count, absolute neutrophil count creatinine, bilirubin, and AST should be performed if early termination occurred after the week 8 study visit but prior to the week 12 study visit.

Laboratory Evaluation for Participants who Meet Case Definition of Potential Rifamycin Hypersensitivity Syndrome

For participants meeting the case definition the following tests should be obtained at the first opportunity after the patient develops symptoms (acute phase) AND then again at 10-35 days after recovery from the event (convalescent phase): serum alanine aminotransferase (ALT), serum total bilirubin, serum creatinine, complete blood count (CBC), 10 cc of blood collected in a red top tube, clotted 30-60 minutes, centrifuged and stored frozen at -50˚C or colder in a plastic tube.

8.2.2 Special Assays or Procedures

At baseline, week 8, and at the time of suspected rifamycin hypersensitivity should it occur, serum will be collected and stored frozen for future evaluation of soluble factors (such as anti-rifamycin antibodies) potentially associated with hypersensitivity. These assays will be performed at Johns Hopkins; shipping issues are described below. Results of these research assays will not be used for clinical decision-making.

8.2.3 Specimen Preparation, Handling, and Shipping

8.2.3.1 Instructions for Specimen Preparation, Handling, and Storage

Standard (universal) precautions will be used during handling of biological specimens.

Sputum specimens and cultures thereof will be handled in accordance with local infection control guidelines. Sputum itself will not be stored.

Sputum AFB smears will be prepared, handled, and stored according to usual routine laboratory practices.

For each participant, the baseline culture that is positive for *M tuberculosis*, as well as well as the first treatment failure isolate (if applicable), will be stored frozen in a -20º freezer per local SOPs in a secure location on-site until completion of the study, at which time they will be destroyed. These frozen cultures will be labeled with participant study identifier and date that the sputum specimen was obtained.

Blinding of mycobacteriology laboratory personnel: mycobacteriology laboratory personnel will be blinded as to the treatment assignment and treatment status of study subjects. No information about study subject treatment assignment or treatment status will be transmitted to mycobacteriology laboratory personnel; study identifiers will not contain information that can be used to independently deduce treatment assignment.

Serum and plasma will be collected and stored according to the schedule indicated in Section 8.2 and the Schedule of Events Table (Appendix), and according to procedures in the procedures manual. Stored specimens will be labeled with the participant study identifier and date and time of collection.

8.2.3.2 Specimen Shipment

Stored frozen serum will be shipped to Johns Hopkins and stored frozen plasma will be shipped to the Infectious Disease Pharmacokinetics Laboratory under the direction of Dr. Charles Peloquin in accordance with international shipping regulations.

9 ASSESSMENT OF SAFETY

9.1 Methods and Timing for Assessing, Recording, and Analyzing Safety Parameters

9.1.1 Adverse Events

The package inserts are the primary sources of risk information for moxifloxacin, isoniazid, rifampin, rifapentine, pyrazinamide, and ethambutol. Information including tolerability, toxicity, and drug-drug interactions for each of the study medications is included in Section 2.

Safety monitoring will focus on assessments for the most frequent toxicities associated with each of the study medications, yet also be sufficiently open-ended to capture unexpected AEs and toxicities. The most frequent toxicities associated with study medications are as follows: hepatotoxicity (isoniazid, pyrazinamide, rifampin, rifapentine); ocular toxicity (ethambutol); cardiotoxicity (moxifloxacin), hyperuricemia (pyrazinamide, rifapentine). Concomitant medications will be solicited at all study visits and will focus on medications that have drug-drug interactions with rifampin and/or rifapentine, and medications that have potential for cardiotoxicity. The timing for assessment and recording of safety parameters is indicated in the Schedule of Procedures/Evaluations (Appendix A). Information about AEs and SAEs will be obtained at each study visit of the intensive phase and the continuation phase.

SOLICITED EVENTS

The following AEs will be collected as solicited events:

- New medical diagnosis
- Worsening of pre-existing symptoms or medical condition to a grade 3 or higher
- Grade 3 or higher toxicity or event (potentially medication related OR other cause)

Starting from the first dose of study medication, at each study visit, each participant will be queried about the following signs/symptoms: fevers, sweats, cough, rash, itching, jaundice, nausea, vomiting, diarrhea, loss of appetite, bad taste in mouth, vision problems, numbness/tingling of extremities, headache, dizziness, insomnia, joint pain, palpitations, syncope.

At each study visit during intensive phase and at the week 12 visit, laboratory testing will include: AST, total bilirubin, creatinine, hemoglobin, platelet count, and absolute neutrophil count.

Pregnancy will not be considered an AE or SAE. Pregnancy testing will be repeated at week 4 for all women of childbearing potential. At weekly visits during intensive phase and at the week 12 visit, women of child-bearing potential will be asked if they are pregnant, the date of last menstrual period, and will be offered a pregnancy test. Handling and reporting of pregnancy is described below under section 9.2.3.

UNSOLICITED EVENTS

Starting from first dose of study medication, at each study visit, unsolicited events will be captured by asking each study participant about other symptoms that occurred since the prior study visit.

REPORTING AEs AND SAEs

In accordance with the FDA's Code of Federal Regulations and with the Brazilian Regulations (resolution CNS 251/97), the sponsor of this trial and the participating investigators are responsible for reviewing all information relevant to the safety of the study drugs.

Information about AEs and SAEs will be obtained at each study visit, according to the study timetable.

9.1.1.1 Definition of Adverse Event

An AE is any untoward medical occurrence in a study subject administered the study intervention and does not necessarily have a causal relationship with the study intervention. An AE can therefore be any unfavorable and unintended sign (including an abnormal laboratory finding), symptom, or disease temporally associated with the use of the study intervention, whether or not related to the study intervention.

Severity of Event: All AEs will be assessed by the clinician using a protocol defined grading system (see Appendix C). For events not included in the protocol defined grading system, the following guidelines will be used to quantify intensity.

Mild: events require minimal or no treatment and do not interfere with the patient's daily activities.

Moderate: events result in a low level of inconvenience or concern with the therapeutic measures. Moderate events may cause some interference with functioning.

Severe: events interrupt a patient's usual daily activity and may require systemic drug therapy or other treatment. Severe events are usually incapacitating.

Life threatening: any adverse drug experience that places the patient or subject, in the view of the investigator, at immediate risk of death from the reaction as it occurred, ie, it does not include a reaction that had it occurred in a more severe form, might have caused death.

Changes in the severity of an AE will be documented to allow an assessment of the duration of the event at each level of intensity to be performed. Adverse events characterized as intermittent require documentation of onset and duration of each episode.

Relationship to Study Products: The clinician's assessment of an AE's relationship to the study drugs is part of the documentation process, but it is not a factor in determining what is or is not reported in the study. If there is any doubt as to whether a clinical observation is an AE, the event should be reported. All AEs must have their relationship to study product assessed using the terms: associated or not associated. In a clinical trial, the study product must always be suspect. To help assess, the following guidelines will be used.

Associated – The event is temporally related to the administration of the study product and no other etiology explains the event. The event ablates with discontinuation of the study product and reappears with re-challenge. The event is known to occur in association with study product or with similar class of study product.

Not Associated – The event is temporally independent of study product and/or the event appears to be explained by another etiology.

9.1.2 Serious Adverse Events

Serious Adverse Event (SAE): An SAE is defined as an AE that meets one of the following conditions:

- Death during the period of protocol defined surveillance
- Life-threatening event (defined as a subject at immediate risk of death at the time of the event)
- An event requiring inpatient hospitalization or prolongation of existing hospitalization during the period of protocol defined surveillance
- Results in congenital anomaly or birth defect
- Results in a persistent or significant disability/incapacity
- Any grade 4 toxicity or event, including worsening of pre-existing symptoms or medical condition to grade 4.
- Grade 3 hepatoxicity will be administratively handled as an SAE (and will trigger ISM evaluation, as well as prompt reporting according to Section 9.2.1.) The rationale is that grade 3 hepatotoxicity is a component of the halting rules.
- Any other important medical event that may not result in death, be life threatening, or require hospitalization, may be considered a serious adverse experience when, based upon appropriate medical judgment, the event may jeopardize the subject and may require medical or surgical intervention to prevent one of the outcomes listed above. Examples of such medical events include allergic bronchospasm requiring intensive treatment in an emergency room or at home, blood dyscrasias or convulsions that do not result in inpatient hospitalization, or the development of drug dependency or drug abuse.

All SAEs will be:

- recorded on the appropriate SAE case report form (CRF)
- followed through resolution by a study clinician, or followed until the PI or sub-investigator deems the event to be chronic or the patient to be stable.
- reviewed and evaluated by a study clinician and the ISM

9.1.3 Procedures to be followed in the Event of Abnormal Laboratory Test Values or Abnormal Clinical Findings

Toxicities will be defined according to the National Cancer Institute Common Toxicity Criteria, version 2.0.

Collection of laboratory data to monitor toxicity will be limited to parameters that are relevant to safety, study outcome measures, and clinical outcome, and will include:

- Hematology: hemoglobin, platelet count, absolute neutrophil count
- Biochemistry: AST, total bilirubin, creatinine
- Sputum smear for AFB
- Sputum culture for *M. tuberculosis*

In general, for grade 1 toxicities, the participant will be followed carefully and the study drugs will be continued.

For grade 2 toxicities, the participant will be followed more carefully, with additional laboratory and/or clinic visits as necessary, and the study drugs temporarily held at the investigator's discretion.

For any grade 3 toxicity that, in the investigator's judgment is due to study drug(s), the causative study drug(s) should be held. The clinician should exclude other possible causes of the event before discontinuing study drug(s). When possible, concomitant medications should be held first at the discretion of the investigator if he/she suspects they are contributing to toxicity. Depending on the nature and severity of the toxicity, the degree to which it resolves, and/or the emergence of alternative explanations for the toxicity or the subject's deterioration, the study drug(s) may be restarted at the discretion of the investigator. For any recurring grade 3 or grade 4 toxicity the study drugs should be temporarily held and may be permanently stopped at the discretion of the investigator.

Any participant with grade 4 renal, hepatic, cardiac, or hematological toxicity will be immediately discontinued from study therapy. The laboratory test or clinical finding in question will be reassessed as soon as possible. Discontinuation may be temporary or permanent, and the repeat test will guide management of the event as follows:

If the repeat assessment shows toxicity of grade 3 or lower, then the participant will be managed at the discretion of the investigator with regard to the re-administration of study drugs, and otherwise according to the toxicity level of the repeat assessment.

If the repeat assessment shows grade 4 toxicity, then the participant will be permanently discontinued from study medications. Further treatment of TB will be directed by the investigator on an individualized basis. The participant will continue to be followed for study monitoring purposes.

For grade 4 toxicities other than renal, hepatic, cardiac, or hematological toxicities, study drugs will be temporarily held and may be restarted or permanently stopped at the discretion of the investigator.

9.2 Reporting Procedures

Adverse Events

All AEs including local and systemic reactions not meeting the criteria for SAEs should be captured on the appropriate CRF. Information to be collected includes event description, time of onset, investigator assessment of severity, relationship to study product, and time of resolution/stabilization of the event. All AEs occurring while on study must be documented appropriately regardless of relationship.

Any medical condition that is present at the time that the patient is screened should be considered as baseline and not reported as an AE. However, if that condition deteriorates at any time during the study it should be recorded as an AE.

9.2.1 Serious Adverse Events

All SAEs will be:

- recorded on the appropriate AE CRF
- followed through resolution by a study clinician, or until the PI or sub-investigator deems the event to be chronic or the patient to be stable.
- reviewed by a study physician and the ISM

Any AE considered serious by the Principal Investigator (PI) or Subinvestigator or which meets the aforementioned criteria must be submitted to the Johns Hopkins and Brazilian IRBs per their guidelins

Comitê de Ética em Pesquisa do HUCFF/UFRJ<br>Rua Rodolfo Rocco, n 255, 1 andar,<br>Cidade Universitária, Rio de Janeiro, Brasil.<br>Cep 21941-913	Johns Hopkins Medicine<br>Office of Human Subjects Research<br>Institutional Review Boards<br>1620 McElderry Street, Suite B-1300<br>Baltimore, MD 21205-1911
Comite de Etica em Pesquisa da Secretaria Municipal de Saude e Defesa Civil,<br>Rua Afonso Cavalcanti, 455 bloco01 sala 715,<br>Cidade Nova, Rio de Janeiro RJ, Brasil CEP 20211-901	The Ethics Committee for Research (CEP) of the Sergio Arouca National School of Public Health – ENSP,<br>Rua Leopoldo Bulhões, 1.480 – Térreo. Manguinhos - Rio de Janeiro - RJ

The study clinician will complete an Adverse Event Form within the following timelines:

All deaths and immediately life threatening events, whether associated or not associated, will be recorded on the Adverse Event Form and sent within 24 working hours of site awareness.

Serious adverse events other than death and immediately life threatening events, regardless of relationship, will be reported by the site within 72 working hours of becoming aware of the event.

Solicited Events that are not serious will be reported to the ISM at intervals provided in the Adverse Event SOP.

All SAEs will be followed until satisfactory resolution or until the PI or Subinvestigator deems the event to be chronic or the patient to be stable.

The IND sponsor is responsible for submitting IND safety reports to the FDA, as necessary per 21 CFR 312.32.

9.2.2 Regulatory Reporting

This study will be conducted under an investigator-initiated IND. Accordingly, the principal investigator will report events that are both serious and unexpected and that are associated with study product(s) to the FDA within the required timelines as specified in Title 21 of the Code of Federal Regulations (CFR) Part 312.32: fatal and life-threatening events within 7 calendar days (by phone or fax) and all other SAEs in writing within 15 calendar days. All serious events designed as “not associated” to study product(s), will be reported to the FDA at least annually in a summary format. These will also be reported to the Brazilian IRB per their requirements.

9.2.3 Reporting of Pregnancy

Pregnancy will not be considered an AE or SAE. Women who become pregnant during the intensive phase of TB therapy will be taken off of their assigned treatment regimen, and treated for TB according to national guidelines. With the subject’s permission, she will continue to be followed, and pregnancy outcome will be recorded and reported. Women who become pregnant during the continuation phase will remain in the study provided other exclusion events do not occur.

9.3 Type and Duration of Follow-up of Subjects after Adverse Events

Adverse event follow-up will be reported on a designated form as soon as possible after the resolution or stabilization of the AE, but no later than 45 days after the AE was reported. AEs and SAEs will be followed, until resolved or considered stable, per the study schedule of evaluations, or more often at the discretion of the investigator.

9.4 Halting Rules

Enrollment will be temporarily halted and a formal DSMB safety review will be undertaken if either or both of the following occur:

a) 3 or more SAEs attributed to study therapy in the experimental HPZM arm
b) 6 or more participants with grade 3 or higher hepatotoxicity in the experimental HPZM arm

No early stopping rules will be formally adopted.

9.5 Safety Oversight

An Independent Safety Monitor (ISM) and a Data Safety and Monitoring Board (DSMB) will provide safety oversight.

The ISM is a physican at the enrollment site with relevant expertise whose primary responsibility is to provide independent safety monitoring in a timely fashion. Participation is for the duration of the study. This is accomplished by review of SAEs, immediately after they occur, with follow-up through resolution. The ISM should be able to readily access participant records in real time. The primary focus of the ISM is to independently review all SAEs and thoroughly investigate those events considered unexpected. Clinical and laboratory data, clinical records, and other study-related records should be made available for ISM review. The ISM may be a faculty member at the Federal University of Rio de Janeiro, but will not be under the direct supervision of the PI. It is the responsibility of the PI to ensure that the ISM is appraised of all new safety information relevant to the study product and the study. This includes providing the ISM with a copy of the study product package inserts in advance and all safety reports issued by the sponsor. The ISM will review all protocol revisions and may receive other documents related to the study. The ISM will review all SAEs which occur during the course of the study. The ISM may transiently halt enrollment. The ISM will contact the PI at the enrollment site and the DSMB chair for any event that needs further evaluation. The ISM will be a member of the DSMB.

The DSMB will be composed of at least 1 expert in clinical aspects of TB, one or more biostatisticians, and one or more experts in clinical trials conduct/methodology. An early safety evaluation will be performed when the first 40 patients (approximately 20 per arm) have completed 8 weeks of study treatment; safety data will be reviewed by the DSMB. Subsequently, the DSMB will meet after enrollment of every 75 patients to assess safety for each arm of the study. If halting rules are initiated, more frequent meetings may be held. The DMSB will operate under the rules of an approved charter that will be written at the organizational meeting of the DSMB. At this time, each data element that the DSMB needs to assess will be clearly defined. The DSMB will assess interim/cumulative data for evidence of study-related adverse events, data quality, completeness and timeliness, demographic information on study participants, factors that might affect the study outcome or compromise the confidentiality of the trials (such as protocol deviations) and factors external to the study such as scientific or therapeutic developments that may impact participant safety or the ethics of the study.

The DSMB should conclude each review with their recommendations to the study sponsor as to whether the study should continue without change, be modified, or terminated.

10 CLINICAL MONITORING

Site Monitoring will be conducted to ensure that human subject protection, study procedures, laboratory procedures, study intervention administration, and data collection processes are of high quality and meet sponsor, CGP/ICH, and regulatory guidelines, and that the study is conducted in accordance with the protocol and sponsor SOPs.

Site visits will be made at standard intervals and may be made more frequently as required. Monitoring visits will include, but are not limited to, review of regulatory files, accountability records, CRFs, informed consent forms, medical and laboratory reports, and protocol compliance. Study monitors will meet with investigators to discuss any problems and actions to be taken and document visit findings and discussions.

10.1 Site Monitoring Plan

A site monitoring plan will be developed and described in detail in a separate monitoring plan document.

11 STATISTICAL CONSIDERATIONS

11.1 Study Hypotheses

Overview and Study Objectives

There is a critical need for the identification of more potent intensive phase regimens in order to identify drug combinations that may shorten the duration of TB therapy. Reproducible data in the murine model demonstrate significantly enhanced potency of rifapentine-based regimens compared to standard daily therapy during the intensive phase (first 8 weeks) TB therapy. In addition, in mice, substitution of moxifloxacin for isoniazid leads to faster sterilization of lungs and may be a more compatible companion drug for rifapentine based on its similar half-life. The proposed study will be a two-center, randomized, open-label trial of an experimental regimen versus a standard regimen during the intensive phase of TB treamtnet. The experimental regimen will be comprised of isoniazid, rifapentine (7.5 mg/kg/dose), pyrazinamide, and moxifloxacin (plus Vitamin B6) administered daily. The standard regimen will be comprised of isoniazid, rifampin, pyrazinamide, and ethambutol (plus vitamin B6) administered daily. We will compare the proportions in each treatment arm having negative sputum cultures at completion of 8 weeks of intensive phase TB therapy. In addition, we will assess the safety and tolerability of each treatment regimen. We will also compare the time to culture conversion for each treatment arm, the proportions of patients (by treatment arm) who develop treatment failure, and the antimicrobial activity and safety of each regimen in HIV-seropositive and HIV-seronegative individuals. Demonstration of safety and improved sputum culture conversion during the intensive phase of therapy with the experimental regimen would provide rationale for larger Phase III clinical trials using TB relapse as a primary endpoint.

Study Hypotheses

The proposed study is a **superiority trial** with respect to the primary outcome of proportion of subjects, by treatment arm, having negative sputum cultures at the end of the intensive phase of TB treatment**.** Rationale: The overall long-term goal is to identify new TB treatments that are more potent than the existing standard treatment, and that consequently can shorten the duration of TB therapy. Therefore the lesser objective of establishing non-inferiority is not of substantial interest, since demonstration of non-inferiority (of a novel TB treatment regimen compared to the standard regimen) would not provide a scientifically valid basis for further evaluation of that novel regimen in subsequent Phase III studies of treatment shortening.

Primary null hypothesis: For the treatment of smear positive, culture-confirmed pulmonary TB, there will be no difference, between the experimental and control treatment arms, in the proportion of subjects that are sputum culture negative (for *M. tuberculosis*) at the end of 8 weeks of intensive phase therapy (treatment effect ≤ 0, non-superiority).

Primary alternative hypothesis: For the treatment of smear positive, culture-confirmed pulmonary TB, the proportion of subjects that are sputum culture negative (for *M. tuberculosis*) at the end of 8 weeks of intensive phase therapy will be higher in the experimental arm than in the control arm (treatment effect > 0; superiority).

11.2 Sample Size Considerations

Outcome measure used for calculations: proportion of subjects having negative sputum cultures at the end of intensive phase therapy in each treatment arm.

Test statistic: The primary analysis will compare proportions of patients with negative cultures (patients with negative cultures/all patients eligible) using the chi-square or Fisher's exact test, as appropriate.

Null and alternate hypothesis for primary outcome

H_0: For the treatment of smear positive, culture-confirmed pulmonary TB, there will be no difference, between the experimental and control treatment arms, in the proportion of subjects that are sputum culture negative (for *M. tuberculosis*) at the end of 8 weeks of intensive phase therapy (treatment effect ≤ 0, non-superiority).

H_a: For the treatment of smear positive, culture-confirmed pulmonary TB, the proportion of subjects that are sputum culture negative (for *M. tuberculosis*) at the end of 8 weeks of intensive phase therapy will be higher in the experimental arm than in the control arm (treatment effect > 0; superiority).

Type I error rate: Power calculations based on a Type I error rate (2-sided α level) of 0.05.

Type II error rate: Power calculations based on a Type II error rate (β) of 0.20.

We estimate that in the standard control HRZE arm, approximately 63% of subjects will have negative sputum cultures at the end of intensive phase therapy. In a recent phase II pulmonary TB treatment study performed at Hospital Universitario Clementino Fraga Filho in Rio de Janeiro, among subjects in the standard control HRZE arm, the proportion of subjects having negative sputum cultures (for *M tuberculosis*) at the end of 8 weeks of intensive phase therapy was 63% (45 of 72) (M. Conde, personal communication). We hypothesize that substitutions of rifapentine for rifampin, and moxifloxacin for ethambutol will improve the potency of the experimental regimen by 20% (from 63% to 83% culture negative at completion of 8 weeks of TB treatment). Of note, the addition of pyrazinamide to isoniazid-containing intensive phase TB treatment has been shown to increase the percentage of individuals who are culture negative at the end of 8 weeks by approximately 13%; this degree of improvement has subsequently been shown to correlate with ability to shorten overall duration of TB treatment.

To detect an increase in culture conversion from 63 to 83% with a two-sided test at the 0.05 level with 80% power requires 86 individuals per arm (172 per study). We have further increased our calculated sample size by 20% to compensate for enrolled patients who are subsequent found to be protocol ineligible (and removed from the analysis of sputum culture conversion) due to missing baseline cultures, baseline cultures positive for a mycobacterium other than *M. tuberculosis*, or baseline drug resistance – therefore 108 individuals per arm (216 per study) will be required.

Sample size: Approximately 108 subjects per each of 2 treatment groups, for a total of 216 subjects.

This sample size is not anticipated to be sufficient to address the secondary endpoint of treatment failure, since treatment failure is anticipated to be rare. However, treatment failure is included as a secondary endpoint since any treatment failure is clinically relevant. The proposed sample size is not anticipated to be sufficient to address secondary analyses of culture conversion and toxicity in HIV-positive versus HIV-negative persons, since fewer than 10% of study subjects are expected to be HIV-positive. However, the proposed sample size may provide a general estimate of microbiological activity and toxicity of the experimental regimen in HIV-positive subjects, and this information will be critical for the design of future Phase III trials.

11.2.1 Safety Review

Safety review will be performed by an independent DSMB after enrollment of every 75 patients. In addition, an early safety evaluation will be performed when the first 40 patients (approximately 20 per arm) have completed 8 weeks of study treatment; safety data will be reviewed by the DSMB. The DSMB will consist of at least 1 expert in clinical aspects of TB, one or more biostatisticians, and one or more experts in clinical trials conduct/methodology. The rationale for the study will be presented to the DSMB at its first meeting. Special meetings of the DSMB may be called if unusual AEs are noted. In addition, an independent safety monitor (a member of the DSMB) will review all SAEs immediately after they occur. No early stopping rules will be formally adopted.

11.2.2 Efficacy Review

No interim analyses regarding efficacy are planned for this Phase 2 trial.

11.3 Final Analysis Plan

For the analysis of proportions of subjects, per treatment arm, who are culture negative at the end of intensive phase, the primary analysis will be a modified intention-to-treat (MITT) analysis, in which missing data due to death or loss to follow-up will be regarded as microbiologic failure. An additional analysis of the MITT group will be conducted in which the last available culture result will be used (last observation carried forward). The MITT group will include all randomized patients who took at least one dose of study medicines and who remain study eligible based on baseline culture growth and *M. tuberculosis* susceptibility pattern. A secondary analysis will be performed following the analytic procedures of the British Medical Research Council studies of 2-month culture-conversion (Fox et al., 1999), in which subjects with the following characteristics will be removed from the analysis of sputum culture conversion: 1) receive non-study therapy for more than 14 days during the intensive phase of therapy, 2) require more than 70 days to complete the intensive phase, 3) have no analyzable baseline sputum culture or, 4) have a baseline isolate is resistant to isoniazid, rifampin, pyrazinamide, moxifloxacin, or any 2 study drugs.

Analysis of safety and toxicity endpoints will be by intention to treat, in which all randomized patients who took at least one dose of study medicines will be included.

Comparison of baseline characteristics of treatment and control groups using chi-square test for categorical variables and t-test for continuous variables will be undertaken to evaluate for potential patient differences. Culture conversion will be analyzed in 2 different ways. The primary analysis will compare proportions of patients with negative cultures (patients with negative cultures/all patients eligible) using the chi-square or Fisher's exact test, as appropriate. In a secondary analysis, the time to sputum culture conversion will be compared with non-parametric methods, using the results of sputum cultures at 2, 4, 6, and 8 weeks of therapy. Proportion of patients discontinuing therapy and proportion of patients developing adverse drug reactions will be compared using the chi-square or Fisher's exact test.

11.4. Pharmacokinetic and Pharmacodynamic Analyses

Optimal sampling methods

Using intensive pharmacokinetic (PK) data from 13 healthy volunteers receiving concomitant moxifloxacinifloxacin and rifapentine (Dooley, 2008) as well as known population pharmacokinetics of rifapentine in TB patients (Langdon, 2005), we first performed a compartmental analysis using WinNonLin (a one-compartment linear model with first-order kinetics and short lag time fit the data best) to determine rifapentine PK parameters. We used similar methodology to determine moxifloxacin PK parameters. Optimal sampling theory using the PK parameter distribution from the intensive PK data (modeled to adjust for the dosing regimen to be employed in the clinical trial) was used to determine the maximally informative five-sample study design using ADAPT software. This sampling design should allow for informative population PK analysis with the most precise and unbiased estimates possible, thus maximizing the probability of defining pharmacodynamic relationships.

Pharmacokinetic/pharmacodynamic analyses

We propose to determine the dose-exposure-response relationship of a moxifloxacin/rifapentine -based regimen for the treatment of TB. We will build a population pharmacokinetic – pharmacodynamic (PK/PD) model to describe the relationship between plasma drug concentrations and relevant study outcomes, in this case 2-month sputum culture conversion and drug-related toxicities, using nonlinear mixed-effects modeling. Population PK-PD analysis elucidating the drug exposure-response relationship will enable quantification of the population means of the PK and PD parameters (ie., fixed effect) and of their variability between subjects (random effect). In addition, these models incorporate the influence of demographic and clinical covariates to help explain inter-individual variability.

The final dataset will be created by pooling all PK and PD measurements. Separate PK/PD models will be built for each drug. First, PK parameters will be estimated using NONMEM software. Based on our understanding of the dispositional pathway of rifapentine, for example, a one-compartment linear PK model will first be tried for rifapentine. A basic structural population PK model with inter-individual variability will be developed. The effects of covariates (e.g. age,

weight, gender, and adherence) on the parameters (in particular AUC and C_{max}) and their variability will be examined graphically and tested for significance to determine the appropriateness of including them in the model. We will then incorporate pharmacodynamic data and develop the best-fitting logistic PK/PD nonlinear mixed effects model using probability of two-month sputum culture conversion as the outcome variable, the drug model (including PK parameters and covariates), and subject-specific random effects. Logistic probability plots (e.g. plotting likelihood of 2-month culture conversion on the Y axis vs. rifapentine AUC on the X axis) can help determine the appropriate type of model, for example an inhibitory sigmoid response model). Using these strategies, we will link exposure (drug concentration) to response (two-month culture conversion) and describe the relationship. Because the PK parameter most predictive of treatment response for rifapentine is not known, we will develop models trying C_{max} or AUC as the exposure variable. For moxifloxacin, the PK parameter most predictive of treatment response is AUC, so this will be used as the exposure variable. If drug-associated toxicity is commonly seen, models will also be built using toxicity as the outcome variable. Bootstrapping will be used to construct confidence intervals for final model parameters. Discrepancy measures will be used to qualify the final PK-PD model.

12 SOURCE DOCUMENTS AND ACCESS TO SOURCE DATA/DOCUMENTS

Source Documents and Access to Source Data/Documents

Appropriate medical and research records will be maintained for this trial, in compliance with ICH E6 GCP, Section 4.9 and regulatory and institutional requirements for the protection of confidentiality of subjects.

The following individuals and groups will have access to study records:

- Members of the study team
- IRB’s that review the study (including IRB members, staff, and legal counsel)
- The Office of Human Research Protections
- The FDA
- The DMID

Authorized representatives of the sponsor(s), , and regulatory agencies indicated above will be permitted to examine (and when required by applicable law, to copy) clinical records for the purposes of QA reviews, audits and evaluation of the study safety and progress.

13 QUALITY CONTROL AND QUALITY ASSURANCE

Standard operating procedures for quality management will be developed and detailed in a separate Quality Management Plan.

The investigational site will provide direct access to all trial related sites, source data/documents, and reports for the purpose of monitoring and auditing by the sponsor, and inspection by local and regulatory authorities.

Following written standard operating procedures, study monitors will verify that the clinical trial is conducted and data are generated, documented (recorded), and reported in compliance with the protocol, GCP, and the applicable regulatory requirements.

All key study staff will be trained and certified in good clinical practices.

14 ETHICS/PROTECTION OF HUMAN SUBJECTS

14.1 Ethical Standard

The investigator will ensure that this study is conducted in full conformity with the principles set forth in The Belmont Report: Ethical Principles and Guidelines for the Protection of Human Subjects of Research of the US National Commission for the Protection of Human Subjects of Biomedical and Behavioral Research (April 18, 1979) and codified in 45 CFR Part 46 and/or the ICH E6; 62 Federal Regulations 25691 (1997). In addition, the investigator will ensure that this study is conducted in full conformity with the current revision of the Declaration of Helsinki, or with the International Conference for Harmonisation Good Clinical Practice (ICH-GCP) regulations and guidelines, whichever affords the greater protection to the subject. In addition, this study will be conducted in accordance with Brazilian standards set by the National Concil of Health (CNS), National Ethics in Research Commission (CONEP).

14.2 Institutional Review Board

Before they are placed into use, the study protocol and informed consent documents will be reviewed and approved by IRBs of Johns Hopkins University School of Medicine (FWA 00005752), the Federal University of Rio de Janeiro/Hospital Universitario Clementino Fraga Filho (FWA 00000377), the Municipal Health Secretary of Rio de Janeiro (FWA 00010761), and the Oswaldo Cruz Foundation, Sergio Arouca School of Public Health (FWA 00000389)
Any amendments to the protocol or consent materials will be reviewed and approved by both IRBs before they are placed into use.

14.3 Informed Consent Process

Informed consent is a process that will be initiated prior to the individual's agreeing to participate in the study and will continue throughout the individual's study participation. Extensive discussion of risks and possible benefits of this therapy will be provided by study staff to potential subjects (and, with permission from potential subjects, their families). Potential subjects will receive counseling about study objectives and procedures, potential toxicities, and the informed consent process. Consent forms describing in detail the study interventions/products, study procedures and risks will be given to the subject and written documentation of informed consent will be required prior to starting intervention/administering study product. Consent forms will be IRB approved, and written in Portuguese for the Brazil sites; English versions will be available for IRB and sponsor review. The subject will be asked to read and review the document. Upon reviewing the document, the person obtaining consent will explain the research study to the subject and answer any questions that may arise. For subjects who speak and understand the language used in the consent document, but are unable to read or write, all of the information in the consent form will be communicated verbally, in the presence of an adult witness who is not a member of the study team; informed consent requires the signature or mark of the subject. The subject will sign or mark the informed consent document prior to any procedures being done specifically for the study. The subjects will sign the informed consent document prior to any procedures being done specifically for the

study. The subjects will have the opportunity to discuss the study with their surrogates or think about it prior to agreeing to participate. The subjects may withdraw consent at any time throughout the course of the trial. A copy of the signed and dated informed consent document will be given to the subjects for their records. The rights and welfare of the subjects will be protected by emphasizing, to subjects, that the quality of their medical care will not be adversely affected if they decline to participate in this study.

During the informed consent process, the subject will receive information about compensation for study participation. Specifically, subjects will not be paid for study participation. Study subjects will receive a voucher for transportion to the hospital/clinic, and food coupons for the duration of time they are at the hospital.

14.3.1 Informed Consent/Assent Process (in Case of a Minor)

Not applicable. All study subjects will be ≥ 18 years of age.

14.4 Exclusion of Women, Minorities, and Children (Special Populations)

Children will be excluded from this study, since fluoroquinolones are relatively contraindicated in children, and since Brazilian law prohibits enrollment of children in drug trials unless the drugs are for a specific pediatric indication.

Pregnant or breast-feeding women will be excluded from this study, since fluoroquinolones are relatively contraindicated in these groups.

14.5 Subject Confidentiality

This study will be performed in accordance with U.S. and Brazilian standards for protection of privacy of identifiable health information.

All study records will be managed in a secure and confidential fashion. Study records will be maintained in locked cabinets, and computer records will be password protected. Access to study records will be restricted to specified team members. Methods for secure data handling are detailed below in Section 15.

The study monitor or other authorized representatives of the sponsor may inspect all documents and records required to be maintained by the Investigator, including but not limited to, medical records (office, clinic or hospital) and pharmacy records for the subjects in this study. The clinical study site will permit access to such records.

14.6 Study Discontinuation

In the event that the study is discontinued (e.g. by the investigator, the sponsor, and/or regulatory groups), subjects on study phase treatment will continue to receive TB treatment in

accordance with national guidelines. Treatment will be offered free of charge at the Hospital Universitario Clementino Fraga Filho and TB clinics of the Municipal Health Department of Rio de Janeiro (Brazilian subjects), or at the subject's local health deparment (Johns Hopkins site subjects).

14.7 Future Use of Stored Specimens

Baseline and treatment failure *M. tuberculosis* isolates will be stored frozen at –50ºC or colder, labeled with the study identifier number and the date of sputum collection. Stored *M. tuberculosis* isolates will be used only for purposes of study-related drug susceptibility testing and will be destroyed at the end of the study.

Serum will be obtained from all participants at baseline and at Day 56, and also from individuals who meet criteria for potential rifamycin hypersensitivity. These sera will be stored at -50˚C or colder and will be tested for immunological factors potentially related to rifamycin hypersensitivity and tuberculosis and will be destroyed at the end of the study.

Plasma collected during the PK sampling will be destroyed after processing.

15 DATA HANDLING AND RECORD KEEPING

Data handling and record keeping will be performed according to procedures that will be developed in a detailed Data Management Operations Manual. The following types of forms will be used in this trial:

- Informed consent forms for screening and enrollment, in English and translated into Portuguese
- Eligibility screening forms
- Data collection forms that document enrollment, follow-up visits, and specimen collection
- Administrative forms that document participant deaths, termination from study drugs, and work up and treatment of AEs and treatment failure

A participant's name will be collected on study specific documents only one time, and this information will be kept on a form that does not contain any test results, and that is filed separately from forms that do contain test results. Each participant will be assigned a unique study ID number. This number will be recorded on each data collection form to facilitate linkage of data. The study ID number will be used on data collection forms; names and other obvious identifiers will not be used on data collection forms. Laboratory specimens and results will contain the subject's name as the results are required to be filed in the patient's hospital record. PK specimens will be labeled with only the study ID.

All data collection forms will be stored in locked files in a secure area. Access to study records and data files will be limited to study personnel, including staff from JHU, the NIH and its designees, the FDA, Johns Hopkins University and the Federal University of Rio de Janeiro.

All forms will be reviewed prior to data entry for accuracy, consistency, and completeness by designated study staff.

For banked *M. tuberculosis* isolates, the study ID number, date of collection, specimen volume, and freezer location will be recorded on the laboratory requisition forms and entered into the computer.

15.1 Data Management Responsibilities

The on-site principal investigator and the data manager will be responsible for the accuracy, completeness, and storage of source records and study data collection forms. The study team and data entry staff will review source documents and laboratory reports to ensure accuracy and completeness. The site staff will maintain logs to record dates of completed and upcoming clinic visits and specimen collections.

15.2 Data Capture Methods

All forms will be double data key entered and verified according to procedures that will be developed in a detailed Data Management Operations Manual.

15.3 Types of Data

Data for this study will include safety, laboratory (microbiology), and clinical history. Safety data will not be collected in a separate database for this study.

15.4 Timing/Reports

The timing of reports will be detailed in the Data Management Operations Manual. Briefly, safety/tolerability data will be reviewed by the DSMB after enrollment of the first 40 subjects and then after enrollment of each 75 study subjects; reports for the DSMB will be prepared for the DSMB according to a schedule determined at the first convened DSMB meeting. Data coding will occur at the time of data collection; ongoing logical data queries will be performed.

15.5 Study Records Retention

Within 2 years of completion of the study, identifiers excluding the study ID number will be deleted from computerized and paper data files. Study records will be maintained by the investigator for a minimum of 5 years following discontinuation of the study. The FDA and DMID will be notified prior to study records being destroyed.

15.6 Protocol Deviations

A protocol deviation is any noncompliance with the clinical trial protocol, Good Clinical Practice (GCP), or Manual of Procedures requirements. The noncompliance may be either on the part of the subject, the investigator, or the study site staff. As a result of deviations, corrective actions are to be developed by the site and implemented promptly.

It is the responsibility of the site to use continuous vigilance to identify and report deviations according to the guidelines of the IND sponsor. All deviations from the protocol must be addressed in study subject source documents. Protocol deviations must be sent to the local IRBs in accordance with standard procedures.

16 PUBLICATION POLICY

Following completion of the study, the investigator is expected to publish the results of this research in a scientific journal. The International Committee of Medical Journal Editors (ICMJE) member journals have adopted a trials-registration policy as a condition for publication. This policy requires that all clinical trials be registered in a public trials registry such as ClinicalTrials.gov*, which is sponsored by the National Library of Medicine. Other biomedical journals are considering adopting similar policiesAny clinical trial starting enrollment after 01 July 2005 must be registered on or before patient enrollment. It is anticipated that the results of this study will be submitted for publication in a peer-reviewed scientific journal. Authorship will be extended to the following individuals: study PI(s); site PI; and individuals having major contribution to the study design, implementation, data analysis, and preparation of the written manuscript.

Prior to submission for presentation or publication, any materials derived wholly or in part from this study must be submitted to the study PI(s), site PI, and all co-authors for review.

*Journal Citation:

De Angelis C, Drazen JM, Frizelle FA, Haug C, Hoey J, Horton R, et al. Clinical trial registration: a statement from the International Committee of Medical Journal Editors. N Engl J Med. 2004;351:1250-1.

17 LITERATURE REFERENCES

Anon. Avelox (moxifloxacin hydrochloride) package insert. Bayer, December 2005

Anon. Priftin (rifapentine) package insert. Aventis, February 2003.

Baciewicz AM, Self TH. Isoniazid interactions. South Med J. Jun 1985;78(6):714-718.

Ball P. Quinolone-induced QT interval prolongation: a not-so-unexpected class effect. J Antimicrob Chemother. May 2000;45(5):557-559.

Ball P. Long-term use of quinolones and their safety. Rev Infect Dis. Jul-Aug 1989;11 Suppl 5:S1365-1370.

Ball P, Stahlmann R, Kubin R, Choudhri S, Owens R. Safety profile of oral and intravenous moxifloxacin: cumulative data from clinical trials and postmarketing studies. Clin Ther. 2004;26:940-50.

Ballow C, Lettieri J, Agarwal V, Liu P, Stass H, Sullivan JT. Absolute bioavailability of moxifloxacin. Clin Ther. Mar 1999;21(3):513-522.

Bemer-Melchior P, Bryskier A, Drugeon HB. Comparison of the in vitro activities of rifapentine and rifampicin against Mycobacterium tuberculosis complex. J Antimicrob Chemother. 2000;46:571-6.

Benator D, Bhattacharya M, Bozeman L, et al. Rifapentine and isoniazid once a week versus rifampicin and isoniazid twice a week for treatment of drug-susceptible pulmonary tuberculosis in HIV-negative patients: a randomised clinical trial. Lancet. Aug 17 2002;360(9332):528-534.

Blumberg HM, Burman WJ, Chaisson RE, et al. American Thoracic Society/Centers for Disease Control and Prevention/Infectious Diseases Society of America: treatment of tuberculosis. Am J Respir Crit Care Med. Feb 15 2003;167(4):603-662.

Bock NN, Sterling TR, Hamilton CD, et al. A prospective, randomized, double-blind study of the tolerability of rifapentine 600, 900, and 1,200 mg plus isoniazid in the continuation phase of tuberculosis treatment. Am J Respir Crit Care Med. Jun 1 2002;165(11):1526-1530.

Burman WJ, Jones BE. Treatment of HIV-related tuberculosis in the era of effective antiretroviral therapy. Am J Respir Crit Care Med. Jul 1 2001;164(1):7-12.

Burman WJ, Gallicano K, Peloquin C. Therapeutic implications of drug interactions in the treatment of human immunodeficiency virus-related tuberculosis. Clin Infect Dis. Mar 1999;28(3):419-429; quiz 430.

Burman WJ, Gallicano K, Peloquin C. Comparative pharmacokinetics and pharmacodynamics of the rifamycin antibacterials. Clin Pharmacokinet. 2001;40(5):327-341.

Burman WJ, Goldberg S, Johnson JL, et al. Moxifloxacin versus ethambutol in the first two months of treatment for pulmonary tuberculosis. Am J Respir Crit Care Med. 2006;174:331-8.

Centers for Disease Control and Prevention. Fatal and severe hepatitis associated with rifampin and pyrazinamide for the treatment of latent tuberculosis infection. MMWR Morb Mortal Wkly Rep. 2001;50:289-291.

Chaisson RE, Clermont HC, Holt EA, Cantave M, Johnson MP, Atkinson J, Davis H, Boulos R, et al., JHU-CDS Research Team. Six-months supervised intermittent tuberculosis therapy in Haitian patients with and without HIV infection. Am J Respir Crit Care Med 1996;154:1034-38.

Codecasa LR, Ferrara G, Ferrarese M, Morandi MA, Penati V, Lacchini C, Vaccarino P, Migliori GB. Long-term moxifloxacin in complicated tuberculosis patients with adverse reactions or resistance to first line drugs. Respir Med 2006;100:1566-72.

Cohn DL, Catlin BJ, Peterson KL, Judson FN, Sbarbaro JA. A 62-dose, 6-month therapy for pulmonary and extrapulmonary tuberculosis. A twice-weekly, directly observed, and cost-effective regimen. Ann Intern Med. Mar 15 1990;112(6):407-415.

Combs DL, O'Brien RJ, Geiter LJ. USPHS Tuberculosis Short-Course Chemotherapy Trial 21: effectiveness, toxicity, and acceptability. The report of final results. Ann Intern Med. Mar 15 1990;112(6):397-406.

Corbett EL, Watt CJ, Walker N, Maher D, Williams BG, Raviglione MC, Dye C. The growing burden of tuberculosis: global trends and interactions with the HIV epidemic. Arch Intern Med. 2003;163:1009-21.

Daniel N, Lounis N, Ji B, et al. Antituberculosis activity of once-weekly rifapentine-containing regimens in mice. Long-term effectiveness with 6- and 8-month treatment regimens. Am J Respir Crit Care Med. May 2000;161(5):1572-1577.

Demolis JL, Kubitza D, Tenneze L, Funck-Brentano C. Effect of a single oral dose of moxifloxacin (400 mg and 800 mg) on ventricular repolarization in healthy subjects. Clin Pharmacol Ther. Dec 2000;68(6):658-666.

Dickinson JM, Mitchison DA. In vitro properties of rifapentine (MDL473) relevant to its use in intermittent chemotherapy of tuberculosis. Tubercle. Jun 1987;68(2):113-118.

Dooley K, Flexner C, Hackman J, Peloquin CA, Nuermberger E, Chaisson RE, Dorman SE. Repeated administration of high-dose intermittent rifapentine reduces rifapentine and moxifloxacinifloxacin plasma concentrations. Antimicrob Agents Chemother 2008;52:4037-42.

Dossing M, Wilcke JT, Askgaard DS, Nybo B. Liver injury during antituberculosis treatment: an 11-year study. Tuber Lung Dis. Aug 1996;77(4):335-340.

Ellard GA. Absorption, metabolism and excretion of pyrazinamide in man. Tubercle. Jun 1969;50(2):144-158.

Ellard GA. The potential clinical significance of the isoniazid acetylator phenotype in the treatment of pulmonary tuberculosis. Tubercle. Sep 1984;65(3):211-227.

Ellard GA, Humphries MJ, Gabriel M, Teoh R. Penetration of pyrazinamide into the cerebrospinal fluid in tuberculous meningitis. Br Med J (Clin Res Ed). Jan 31 1987;294(6567):284-285.

El-Sadr WM, Perlman DC, Matts JP, Nelson ET, Cohn DL, Salomon N, Olibrice M, Medard F, Chirgwin KD, Mildvan D et al. Evaluation of an intensive intermittent-induction regimen and duration of short-course treatment for human immunodeficiency virus-related pulmonary tuberculosis. Tery Beirn Community Programs for Clinical Research on AIDS (CPCRA) and the AIDS Clinical Trials Group (ACTG). Clin Infect Dis 1998;26:1148-58.

Fox W, Ellard GA, Mitchison DA. Studies on the treatment of tuberculosis undertaken by the British Medical Research Council tuberculosis units, 1946-1986, with relevant subsequent publications. Int J Tuberc Lung Dis. Oct 1999;3(10 Suppl 2):S231-279.

Gavin III JR, Kubin R, Choudhri S, Kubitza D, Himmel H, Gross R, Meyer JM. Moxifloxacin and glucose homeostasis. A pooled-analysis of the evidence from clinical and postmarketing studies. Drug Safety 2004;27:671-686.

Gillespie SH, Billington O. Activity of moxifloxacin against mycobacteria. J Antimicrob Chemother. Sep 1999;44(3):393-395.

Harrell RM. Fluoroquinolone-induced tendinopathy: what do we know? South Med J. Jun 1999;92(6):622-625.

Hayem G, Carbon C. A reappraisal of quinolone tolerability. The experience of their musculoskeletal adverse effects. Drug Saf. Dec 1995;13(6):338-342.

Iannini P, Kubin R, Reiter C. Over 10 million patient uses: An update on the safety profile of oral moxifloxacin. Poster presented at: 42nd Interscience Conference on Antimicrobial Agents and Chemotherapy; September 27-30, 2002; San Diego, CA. Poster L-374.

Iannini PB, Kubin R, Reiter C, Tillotson G. Reassuring safety profile of moxifloxacin (letter). Clin Infect Dis 2001;32:1112-4.

Ji B, Lounis N, Maslo C, Truffot-Pernot C, Bonnafous P, Grosset J. In vitro and in vivo activities of moxifloxacin and clinafloxacin against Mycobacterium tuberculosis. Antimicrob Agents Chemother. Aug 1998;42(8):2066-2069.

Ji B, Truffot-Pernot C, Lacroix C, et al. Effectiveness of rifampin, rifabutin, and rifapentine for preventive therapy of tuberculosis in mice. Am Rev Respir Dis. Dec 1993;148(6 Pt 1):1541-1546.

Johnson JL, Hadad DJ, Boom WH, Daley CL, Peloquin CA, Eisenach KD, Jankus DD, Debanne SM, Charlebois ED, Maciel E, Palaci M, Dietze R. Early and extended early bactericidal activity of levofloxacin, gatifloxacin, and moxifloxacin in pulmonary tuberculosis. Int J Tuberc Lung Dis 2006;10:605-12.

Kassim S, Sassan-Morokro M, Ackah A, Abouya LY, Digbeu H, Yesso G, Coulibaly IM, Coulibaly D, Whitaker PJ, Doorly R, et al. Two year follow-up of persons with HIV-1 and HIV-2 associated pulmonary tuberculosis treated with short course chemotherapy in West Africa. AIDS 1995;9:1185-91.

Kay L, Kampmann JP, Svendsen TL, et al. Influence of rifampicin and isoniazid on the kinetics of phenytoin. Br J Clin Pharmacol. Oct 1985;20(4):323-326.

Keung AC, Eller MG, Weir SJ. Single-dose pharmacokinetics of rifapentine in women. J Pharmacokinet Biopharm. Feb 1998;26(1):75-85.

Kaung AC, Eller MG, McKenzie KA, Weir SJ. Single and multiple dose pharmacokinetics of rifapentine in man: Part II. Int J Tubec Lung Dis. 1999;3(5):437-444.

Kohno S, Koga H, Kaku M, Maesaki S, Hara K. Prospective comparative study of ofloxacin or ethambutol for the treatment of pulmonary tuberculosis. Chest. Dec 1992;102(6):1815-1818.

Lacroix C, Tranvouez JL, Phan Hoang T, Duwoos H, Lafont O. Pharmacokinetics of pyrazinamide and its metabolites in patients with hepatic cirrhotic insufficiency. Arzneimittelforschung. Jan 1990;40(1):76-79.

Langdon G, Wilkins J, McFadyen L, McIlleron H, Smith P, Simonsson US. Population pharmacokinetics of rifapentine and its primary desacetyl metabolite in South African tuberculosis patients. Antimicrob Agents Chemother 2005;49:4429-36.

Lettieri J, Vargas R, Agarwal V, Liu P. Effect of food on the pharmacokinetics of a single oral dose of moxifloxacin 400mg in healthy male volunteers. Clin Pharmacokinet. 2001;40 Suppl 1:19-25.

McDonald RJ, Memon AM, Reichman LB. Successful supervised ambulatory management of tuberculosis treatment failures. Ann Intern Med. Mar 1982;96(3):297-302.

Mitchison DA. Assessment of new sterilizing drugs for treating pulmonary tuberculosis by culture at 2 months [letter]. Am Rev Respir Dis. 1993;147:1062.

Miyazaki E, Miyazaki M, Chen JM, Chaisson RE, Bishai WR. Moxifloxacin (BAY12-8039), a new 8-methoxyquinolone, is active in a mouse model of tuberculosis. Antimicrob Agents Chemother. Jan 1999;43(1):85-89.

Newcombe RG, Interval estimation for the difference between independent proportions: comparison of eleven methods. Statistics in Medicine, 1988;17:873-90.

Nijland HM, Ruslami R, Suroto AJ, et al. Rifampicin reduces plasma concentrations of moxifloxacin in patients with tuberculosis. Clin Infect Dis **2007**;45(8):1001-7.

Nuermberger EL, Yoshimatsu T, Tyagi S, et al. Moxifloxacin-containing regimen greatly reduces time to culture conversion in murine tuberculosis. Am J Respir Crit Care Med. Feb 1 2004;169(3):421-426. (2004a).

Nuermberger EL, Yoshimatsu T, Tyagi S, et al. Moxifloxacin-containing Regimens of Reduced Duration Produce a Stable Cure in Murine Tuberculosis. Am J Respir Crit Care Med. Aug 11 2004. (2004b).

Ormerod LP, Horsfield N. Frequency and type of reactions to antituberculosis drugs: observations in routine treatment. Tuber Lung Dis. Feb 1996;77(1):37-42.

Park-Wyllie LY, Juurlink DN, Kopp A, Shah BR, Stukel TA, Stumpo C, Dresser L, Low DE, Mamdani M. Outpatient gatifloxacin therapy and dysglycemia in older adults. New Engl J Med 2006;354:1352-61.

Patel AM, McKeon J. Avoidance and management of adverse reactions to antituberculosis drugs. Drug Saf. Jan 1995;12(1):1-25.

Peloquin CA, Namdar R, Dodge AA, Nix DE. Pharmacokinetics of isoniazid under fasting conditions, with food, and with antacids. Int J Tuberc Lung Dis. Aug 1999;3(8):703-710.

Perriens JH, St. Louis ME, Mukadi YB, Brown C, Prignot J, Pouthier F, Portaels F, Willame JC, Mandala JK, Kaboto M, et al. Pulmonary tuberculosis in HIV-infected patients in Zaire: a controlled trial of treatment for either 6 or 12 months. N Engl J Med 1995;332:779-84.

Pierfitte C, Royer RJ. Tendon disorders with fluoroquinolones. Therapie. Jul-Aug 1996;51(4):419-420.

Pletz MW, De Roux A, Roth A, Neumann KH, Mauch H, Lode H. Early bactericidal activity of moxifloxacin in treatment of pulmonary tuberculosis: a prospective, randomized study. Antimicrob Agents Chemother. Mar 2004;48(3):780-782.

Reith K, Keung A, Toren PC, Cheng L, Eller MG, Weir SJ. Disposition and metabolism of 14C-rifapentine in healthy volunteers. Drug Metab Dispos. Aug 1998;26(8):732-738.

Rodriguez JC, Ruiz M, Climent A, Royo G. In vitro activity of four fluoroquinolones against Mycobacterium tuberculosis. Int J Antimicrob Agents. Mar 2001;17(3):229-231.

Rosenthal IM, Williams K, Tyagi S, et al. Weekly moxifloxacin and rifapentine is more active than the denver regimen in murine tuberculosis. Am J Respir Crit Care Med. Dec 1 2005;172(11):1457-1462.

Rosenthal IM, Williams K, Tyagi S et al. Potent twice-weekly rifapentine-containing regimens in murine tuberculosis. Am J Respir Crit Care Med 2006;174:94-101.

Royer RJ, Pierfitte C, Netter P. Features of tendon disorders with fluoroquinolones. Therapie. Jan-Feb 1994;49(1):75-76.

Schaberg T, Rebhan K, Lode H. Risk factors for side-effects of isoniazid, rifampin and pyrazinamide in patients hospitalized for pulmonary tuberculosis. Eur Respir J. Oct 1996;9(10):2026-2030.

Segev S, Yaniv I, Haverstock D, Reinhart H. Safety of long-term therapy with ciprofloxacin: data analysis of controlled clinical trials and review. Clin Infect Dis. Feb 1999;28(2):299-308.

Singla R, Gupta S, Gupta R, Arora VK. Efficacy and safety of sparfloxacin in combination with kanamycin and ethionamide in multidrug-resistant pulmonary tuberculosis patients: preliminary results. Int J Tuberc Lung Dis. Jun 2001;5(6):559-563.

Sirgel FA, Donald PR, Odhiambo J, et al. A multicentre study of the early bactericidal activity of anti-tuberculosis drugs. J Antimicrob Chemother. Jun 2000;45(6):859-870.

Stass H, Kubitza D. Pharmacokinetics and elimination of moxifloxacin after oral and intravenous administration in man. J Antimicrob Chemother. May 1999;43 Suppl B:83-90.

Stass H, Kubitza D, Schuhly U. Pharmacokinetics, safety and tolerability of moxifloxacin, a novel 8-methoxyfluoroquinolone, after repeated oral administration. Clin Pharmacokinet. 2001;40 Suppl 1:1-9. (2001a).

Stass H, Wandel C, Delesen H, Moller JG. Effect of calcium supplements on the oral bioavailability of moxifloxacin in healthy male volunteers. Clin Pharmacokinet. 2001;40 Suppl 1:27-32. (2001b).

Stass H, Kubitza D. Effects of dairy products on the oral bioavailability of moxifloxacin, a novel 8-methoxyfluoroquinolone, in healthy volunteers. Clin Pharmacokinet. 2001;40 Suppl 1:33-38. (2001c).

Stass H, Bottcher MF, Ochmann K. Evaluation of the influence of antacids and H2 antagonists on the absorption of moxifloxacin after oral administration of a 400mg dose to healthy volunteers. Clin Pharmacokinet. 2001;40 Suppl 1:39-48. (2001d).

Stass H, Schuhly U, Moller JG, Delesen H. Effects of sucralfate on the oral bioavailability of moxifloxacin, a novel 8-methoxyfluoroquinolone, in healthy volunteers. Clin Pharmacokinet. 2001;40 Suppl 1:49-55. (2001e).

Stass H, Kubitza D. Effects of iron supplements on the oral bioavailability of moxifloxacin, a novel 8-methoxyfluoroquinolone, in humans. Clin Pharmacokinet. 2001;40 Suppl 1:57-62. (2001f).

Stass H, Kubitza D. Lack of pharmacokinetic interaction between moxifloxacin, a novel 8-methoxyfluoroquinolone, and theophylline. Clin Pharmacokinet. 2001;40 Suppl 1:63-70. (2001g)

Stass H, Sachse R. Effect of probenecid on the kinetics of a single oral 400mg dose of moxifloxacin in healthy male volunteers. Clin Pharmacokinet. 2001;40 Suppl 1:71-76. (2001h).

Tam CM, Chan SL, Lam CW, et al. Rifapentine and isoniazid in the continuation phase of treating pulmonary tuberculosis. Initial report. Am J Respir Crit Care Med. Jun 1998;157(6 Pt 1):1726-1733.

The Tuberculosis Trials Consortium. Rifapentine and isoniazid once a week versus rifampicin and isoniazid twice a week for treatment of drug-susceptible pulmonary tuberculosis in HIV-negative patients: a randomized clinical trial. *Lancet* 2002;360:528–534.

Tortoli E, Dionisio D, Fabbri C. Evaluation of moxifloxacin activity in vitro against Mycobacterium tuberculosis, including resistant and multidrug-resistant strains. J Chemother. Aug 2004;16(4):334-336.

Valerio G, Bracciale P, Manisco V, Quitadamo M, Legari G, Bellanova S. Long-term tolerance and effectiveness of moxifloxacin therapy for tuberculosis: preliminary results. J Chemother. Feb 2003;15(1):66-70.

Venkatesan K. Pharmacokinetic drug interactions with rifampicin. Clin Pharmacokinet. Jan 1992;22(1):47-65.

Vernon A, Burman W, Benator D, Khan A, Bozeman L. Acquired rifamycin monoresistance in patients with HIV-related tuberculosis treated with once-weekly rifapentine and isoniazid. Tuberculosis Trials Consortium. Lancet. May 29 1999;353(9167):1843-1847.

Veziris N, Lounis N, Chauffour A, Truffot-Pernot C, Jarlier V. Efficient intermittent rifapentine-moxifloxacin-containing short-course regimen for treatment of tuberculosis in mice. Antimicrob Agents Chemother. Oct 2005;49(10):4015-4019.

von Keutz E, Schluter G. Preclinical safety evaluation of moxifloxacin, a novel fluoroquinolone. J Antimicrob Chemother. May 1999;43 Suppl B:91-100.

Wallis RS and Johnson JL. The role of surrogate markers in the clinical evaluation of anti-tuberculous chemotherapy. Curr Med Chem 2005;4.

Weiner M, Burman W, Luo CC, et al. Effects of rifampin and multidrug resistance gene polymorphism on concentrations of moxifloxacin. Antimicrob Agents Chemother **2007**;51(8):2861-6.

Weiner M, Bock N, Peloquin CA, et al. Pharmacokinetics of rifapentine at 600, 900, and 1,200 mg during once-weekly tuberculosis therapy. Am J Respir Crit Care Med. Jun 1 2004;169(11):1191-1197.

Weiner M, Burman W, Vernon A, et al. Low isoniazid concentrations and outcome of tuberculosis treatment with once-weekly isoniazid and rifapentine. Am J Respir Crit Care Med. May 15 2003;167(10):1341-1347.

Woodcock JM, Andrews JM, Boswell FJ, Brenwald NP, Wise R. In vitro activity of BAY 12-8039, a new fluoroquinolone. Antimicrob Agents Chemother. Jan 1997;41(1):101-106.

World Health Organization. Global tuberculosis control: surveillance, planning, financing. WHO Report 2006. Geneva, World Health Organization (WHO/HTM/TB/2006.362).

Yee D, Valiquette C, Pelletier M, Parisien I, Rocher I, Menzies D. Incidence of serious side effects from first-line antituberculosis drugs among patients treated for active tuberculosis. Am J Respir Crit Care Med. Jun 1 2003;167(11):1472-1477.

Yew WW, Chan CK, Chau CH, et al. Outcomes of patients with multidrug-resistant pulmonary tuberculosis treated with ofloxacin/levofloxacin-containing regimens. Chest. Mar 2000;117(3):744-751.

Zabraniecki L, Negrier I, Vergne P, et al. Fluoroquinolone induced tendinopathy: report of 6 cases. J Rheumatol. Mar 1996;23(3):516-520.

Zierski M, Bek E. Side-effects of drug regimens used in short-course chemotherapy for pulmonary tuberculosis. A controlled clinical study. Tubercle. Mar 1980;61(1):41-49.

APPENDIX: SCHEDULE OF EVENTS

Event	Screening	Baseline	Wk 1	Wk 2	Wk 3	Wk 4	Wk 5	W 6	Wk 7	Wk 8	Wk 12	Wk 16	Wk 20	Wk 26[1]	Early Term: Inten.Phase	Early Term: Cont. Phase
CLINICAL																
Informed Consent	X															
Medical History	X															
Demographics	X															
Height	X															
Heart rate, Weight	X		X	X	X	X	X	X	X	X	X	X	X	X	X	X
Concomitant Medications	X	X	X	X	X	X	X	X	X	X	X	X	X	X	X	X
Adverse Events			X	X	X	X	X	X	X	X	X	X	X	X	X	X
Visual Testing	X					X				X	X				X	X[10]
Counseling: study objectives	X															
Counseling: Drug toxicity	X	X	X	X	X	X	X	X	X	X	X	X	X	X		
Interval medical history			X	X	X	X	X	X	X	X	X	X	X	X	X	X
LAB																
Sputum smear and culture	X[5]		X	X	X	X	X	X	X	XX[7]	X	X[8]	X[8]	X[8]	XX[7]	X[8]
Pregnancy evaluation[2]	X[11]	X[9]	X[9]	X[9]	X[9]	X[11]	X[9]	X[9]	X[9]	X[9]	X[9]				X[9,10]	X[10]
HIV test[3]	X															
CD4 count[4]	X															
Hematology (hemoglobin, absolute neutrophil count, platelet count)	X[6]			X		X		X		X	X				X	X[10]
Creatinine, bilirubin, AST,	X[6]			X		X		X		X	X				X	X[10]
Serum potassium	X[6]															
Chest radiograph	X[6]													X[12]		X
Serum for storage[13]	X									X						
PK Sampling[14]					X											

1. For subjects with extension of treatment to 9 months, additional study visits will be performed at weeks 30, 34, and 38, with collection of data as for the Week 26 study visit.
2. For women of child-bearing potential
3. An HIV test need not be performed if the subject has had a positive enzyme-linked immunosorbent assay (ELISA) and western blot test at any time in the past, or written documentation of a negative test within the preceding 3 months or less.
4. CD4 count is required for HIV-positive participants but not for HIV-negative participants. For HIV-positive participants, CD4 count should be performed unless written results are available from a test done within the preceding 3 months or less.
5. An expectorated or induced sputum must be sent to lab that will be used in the study (unless a sputum specimen was sent to that lab within the preceding 3 days or less). Drug susceptibility testing will be performed on all screening cultures that are positive for *M. tuberculosis*.
6. Unless results are available for tests done within the preceding 14 days or less.
7. Two sputum specimens will be obtained at the end of intensive phase therapy. These two specimens must be obtained 0 to 7 days after completion of the intensive phase therapy and prior to the subject receiving more than one dose of continuation phase therapy.
8. Sputum specimens should be obtained until there have been 2 consecutive culture-negative specimens
9. Women of child-bearing potential should be asked if they are pregnant, the date of last menstrual period, and offered a pregnancy test if they think they could be pregnant.
10. Required if early termination occurred after completion of the Week 8 study visit but prior to completion of the Week 12 study visit. Not required if early termination occurred after completion of the Week 12 study visit
11. For women of child-bearing potential, a SERUM pregnancy test must be performed at screening and at week 4.
12. One chest x-ray should be obtained for study purposes; this x-ray is intended to be an end-of-treatment x-ray. Therefore, for individuals in whom TB treatment IS NOT extended, the chest x-ray should be performed at week 26. For individuals in whom TB treatment IS extended, the chest x-ray should be performed at week 38.
13. Serum for storage should also be obtained in any instance of potential rifamycin hypersensitivity.
14. Experimental (rifapentine plus moxifloxacin-containing) arm only
